# Separable Crossover-Promoting and Crossover-Constraining Aspects of Zip1 Activity during Budding Yeast Meiosis

**DOI:** 10.1371/journal.pgen.1005335

**Published:** 2015-06-26

**Authors:** Karen Voelkel-Meiman, Cassandra Johnston, Yashna Thappeta, Vijayalakshmi V. Subramanian, Andreas Hochwagen, Amy J. MacQueen

**Affiliations:** 1 Department of Molecular Biology and Biochemistry, Wesleyan University, Middletown, Connecticut, United States of America; 2 Department of Biology, New York University, New York, New York, United States of America; The University of North Carolina at Chapel Hill, UNITED STATES

## Abstract

Accurate chromosome segregation during meiosis relies on the presence of crossover events distributed among all chromosomes. MutSγ and MutLγ homologs (Msh4/5 and Mlh1/3) facilitate the formation of a prominent group of meiotic crossovers that mature within the context of an elaborate chromosomal structure called the synaptonemal complex (SC). SC proteins are required for intermediate steps in the formation of MutSγ-MutLγ crossovers, but whether the assembled SC structure *per se* is required for MutSγ-MutLγ-dependent crossover recombination events is unknown. Here we describe an interspecies complementation experiment that reveals that the mature SC is dispensable for the formation of Mlh3-dependent crossovers in budding yeast. Zip1 forms a major structural component of the budding yeast SC, and is also required for MutSγ and MutLγ-dependent crossover formation. *Kluyveromyces lactis ZIP1* expressed in place of *Saccharomyces cerevisiae ZIP1* in *S*. *cerevisiae* cells fails to support SC assembly (synapsis) but promotes wild-type crossover levels in those nuclei that progress to form spores. While stable, full-length SC does not assemble in *S*. *cerevisiae* cells expressing *K*. *lactis ZIP1*, aggregates of *K*. *lactis* Zip1 displayed by *S*. *cerevisiae* meiotic nuclei are decorated with SC-associated proteins, and *K*. *lactis* Zip1 promotes the SUMOylation of the SC central element protein Ecm11, suggesting that *K*. *lactis* Zip1 functionally interfaces with components of the *S*. *cerevisiae* synapsis machinery. Moreover, *K*. *lactis* Zip1-mediated crossovers rely on *S*. *cerevisiae* synapsis initiation proteins Zip3, Zip4, Spo16, as well as the Mlh3 protein, as do the crossovers mediated by *S*. *cerevisiae* Zip1. Surprisingly, however, *K*. *lactis* Zip1-mediated crossovers are largely Msh4/Msh5 (MutSγ)-independent. This separation-of-function version of Zip1 thus reveals that neither assembled SC nor MutSγ is required for Mlh3-dependent crossover formation *per se* in budding yeast. Our data suggest that features of *S*. *cerevisiae* Zip1 or of the assembled SC in *S*. *cerevisiae* normally constrain MutLγ to preferentially promote resolution of MutSγ-associated recombination intermediates.

## Author Summary

At the heart of reproductive cell formation is a nuclear division process (meiosis) whereby homologous chromosomes segregate from one another. Meiotic partner chromosomes establish exclusive associations via a patterned distribution of crossover recombination events. During the maturation of recombination intermediates into crossovers, homologous axes are aligned in the context of a striking proteinaceous structure, the synaptonemal complex (SC). While genetic data link the SC with crossovers, it is unclear whether the mature SC structure facilitates crossover formation. Here we describe an interspecies complementation experiment in which we replace the *S*. *cerevisiae* version of an SC structural protein with an ancestrally related version from *K*. *lactis*. Our experiment reveals that, while SC proteins are required, mature full-length SC is dispensable for the formation of SC-associated crossovers in budding yeast. We furthermore discovered that most, but not all, members of a conserved meiotic crossover pathway are required for the crossovers that form in this interspecies context. Our findings strengthen the notion that a primary function of many SC proteins is to facilitate crossover recombination, independent of a role in building the larger SC structure. Furthermore, these data suggest that during normal meiosis in *S*. *cerevisiae* the assembled SC may act to functionally couple key crossover recombination proteins to one another.

## Introduction

The segregation of homologous chromosomes at meiosis I is essential for the formation of haploid reproductive cells. Accurate segregation is dependent on the establishment of one or more associations between homologous chromosomes [1,2]. For most organisms, crossover recombination events in conjunction with sister chromatid cohesion provide the temporary associations needed between homologous chromosomes for their proper alignment and segregation on the meiosis I spindle. Interhomolog crossovers arise via the resolution of joint molecule (JM) intermediates, such as double Holliday junctions (dHJs), that form between homologous partner chromosomes during the repair of programmed, double-stranded DNA breaks (DSBs). The formation of interhomolog crossovers during meiosis depends on meiosis-specific proteins and, in a number of organisms, is temporally and functionally linked to a conserved meiotic chromosomal structure called the synaptonemal complex (SC).

Recombination-based associations between homologs can be cytologically detected and are referred to as chiasmata [1,3–5]. During the maturation of recombination intermediates into crossovers, however, such sites are often obscured by the presence of SC, a prominent, proteinaceous structure assembled along the entire lengthwise interface of aligned homologous chromosomes. The SC has a tripartite organization. One component of the larger structure is established via the multimeric assembly of coiled-coil containing proteins that form transverse filaments [6–8]. Transverse filaments are oriented perpendicular to the long axis of an aligned homolog pair and span the width of the SC, bridging the proteinaceous axes of each chromosome. Chromosome axes are referred to as lateral elements within the context of the mature SC. Additional proteins that make up the mature SC’s “central element” substructure assemble at the midline of the SC’s central region, apparently associating with and perhaps organizing transverse filament proteins. Zip1 is a coiled-coil protein component of the transverse filaments of the budding yeast SC [9,10], while Ecm11, SUMO and Gmc2 are proteins that are incorporated into the central element substructure [11–13].

Several additional proteins that are critical for the elaboration of SC along chromosomes in budding yeast do not appear to form structural components of the complex. These so-called “Synapsis Initiation Complex” (SIC) proteins [14], which include Zip2, Zip3, Zip4 and Spo16, localize at SC assembly (synapsis initiation) sites on meiotic chromosomes, many of which are thought to correspond to sites of ongoing recombination, and remain predominantly distributed as foci on full-length SCs after synapsis is complete [11–13,15–18].

The SC structure is established downstream of initial homology recognition and mediates the close apposition of homologous chromosomes (synapsis) during mid-meiotic prophase; the SC thus forms the context in which the majority of meiotic crossovers mature. The characterization of meiotic mutants has revealed a tight correlation between the presence of SC and the establishment of a proper number and distribution of interhomolog crossover recombination events, raising the possibility that SC structure itself plays a functional role in promoting crossover formation [1,7,19,20]. However, the molecular relationship between SC proteins, SC structure and the processing of recombination intermediates remains uncertain. The SC has also been linked to meiotic checkpoint signaling during meiosis, which can delay or arrest meiotic progression [21,22].

In many species, mutants defective in SC assembly (synapsis) exhibit a deficit in a genetically defined subset of crossovers, sometimes referred to as “class I” events [7,23–29]. SC-associated crossovers rely on SC proteins (SIC proteins and SC structural proteins in budding yeast) and often also rely on specific eukaryotic homologs of the bacterial MutS and MutL mismatch repair proteins (the Msh4/Msh5 and Mlh1/Mlh3 heterodimers, which comprise MutSγ and MutLγ, respectively) to promote the formation, maturation and resolution of the majority of dHJ intermediates that arise during meiosis [23,27,30–39]. The Msh4/Msh5 heterodimer (MutSγ) is capable of forming a “clamp” on double-stranded DNA and can recognize HJ structures [36]; these observations in conjunction with other data have led to the idea that Msh4/Msh5 acts to protect a dHJ intermediate from the anti-crossover activity of helicases such as Sgs1 [40,41]. Alternatively, or in addition, Msh4/Msh5 might promote the formation of a JM structure that can be recognized by a MutLγ-associated resolvase complex (in budding yeast this resolvase complex appears to involve MutLγ and Exo1 [23]), or may directly recruit MutLγ complexes to dHJs [32]. Once targeted, the MutLγ-Exo1 complex presumably resolves dHJ intermediates through its endonuclease activity [23,33,38,42]. The MutSγ complex can be detected cytologically at chromosomal sites where SIC proteins (Zip2, Zip3, Zip4) localize, and although MutSγ is dispensable *per se* for Zip1 elaboration along chromosomes, mutants missing *MSH4* have been reported to exhibit delayed SC formation [30], suggesting the possibility of a complex interplay between the SC assembly process and discrete steps in the processing of DNA intermediates at recombination sites. Precisely how MutSγ and MutLγ complexes collaborate with one another and with SC-associated proteins to process recombination intermediates into interhomolog crossover products is not well understood.

On the other hand, MutSγ-MutLγ-independent crossovers can be detected in many organisms, including budding yeast [26]. Such so-called “class II” crossovers, in budding yeast, are genetically unlinked to SC protein activity and resolution of recombination intermediates associated with this class rely on the Mus81-Mms4, Slx1-Slx4, and/or Yen1 structure-selective endonuclease complexes [23,24,26,28,43]. While these observations suggest a conserved and perhaps functional relationship between the SC and MutSγ-MutLγ, it should be noted that SC-associated crossovers, MutSγ and MutLγ might not be strictly linked in all organisms. *C*. *elegans*, for example, relies on SC proteins and Msh4/Msh5 (MutSγ) for processing recombination intermediates toward an interhomolog crossover fate [44–47] but apparently employs predominantly MUS-81 and XPF-1 endonuclease complexes (presumably instead of MutLγ) to resolve such intermediates [48–50]. *Drosophila* also relies on SC proteins for crossover formation [51] but does not appear to have *msh4* nor *msh5* homologs, and instead uses a meiosis-specific version of mini-chromosome maintenance proteins to perform at least some of roles of MutSγ in processing recombination intermediates [52]. For any of these scenarios, how SC proteins and/or the SC structure might interface with DNA repair enzymes to facilitate a crossover event is poorly understood.

At least in budding yeast, several SC-associated proteins appear to facilitate early steps in MutSγ-MutL-associated crossover formation, prior to the elaboration of full-length SC. Mutant meiotic cells missing either *ZIP2* or *ZIP3* exhibit the same deficit as *msh5* mutants in the accumulation of single end invasion (SEI) and dHJ intermediates, early crossover recombination intermediates that occur largely prior to full-length SC formation [27,35,53]. Although at one recombination hotspot, *zip1* mutants showed a distinctly weaker defect in the accumulation of SEI and dHJ intermediates relative to *zip2*, *zip3* and *msh5* mutants [27], the altered kinetics of SEI and dHJ formation observed in *zip1* mutants has been used to argue that even the SC transverse filament protein Zip1 acts early, prior to its elaboration along chromosomes, to facilitate the formation of qualitatively normal dHJ structures [27,54,55]. Consistent with the idea that SC proteins are involved in recombination independent of their role in elaborating an SC structure along the chromosome, SIC proteins localize to chromosomal sites that are correlated with class I crossover-designated recombination events, even in the absence of full-length SC [14,15,56]. How SC proteins functionally interface with the processes mediated by MutSγ, MutLγ and/or other recombination proteins during crossover formation is unknown: Are SC proteins, particularly SC structural proteins like Zip1, merely forming a scaffold upon which recombination enzymes dock, or do these proteins have a more specialized role in the processing of recombination intermediates?

Furthermore, is there any role for the fully assembled SC structure *per se* in MutSγ-MutLγ-associated crossover formation? Later steps in the maturation of crossovers occur in the context of full-length SC, and MutLγ-mediated resolution occurs concomitant with SC disassembly in budding yeast; the latter two events are triggered by Cdc5 activity at a mid-late prophase transition marked by elevated Ndt80 activity [3,19]. As the relevant protein targets of Cdc5 with respect to these events remain unknown, it is unclear whether the process of SC disassembly is normally mechanistically linked to the resolution of recombination intermediates into crossovers.

As noted above, mutants lacking *ZIP1* appear to have a weaker defect in accumulating JM structures (presumed to be dHJs) relative to mutants missing *ZIP2*, *ZIP3* or *MSH5* [27], but *zip1* mutants nevertheless lack MutSγ-MutLγ-associated crossovers [23,25,27,30,34]. This observation is consistent with a role for the mature SC in facilitating later steps in the successful maturation of MutSγ-MutLγ interhomolog recombination events. Other studies support the possibility that SC is dispensable for generating meiotic crossovers in budding yeast. These studies describe mutant situations in which SC assembly is disrupted yet crossovers form (i.e. in the absence of normal SUMOylation [11,13] and in the absence of the meiosis-specific chromosomal axis protein Red1 [55]. However, these prior investigations did not explore whether the apparently SC-independent crossovers form through a canonical SIC protein/ MutSγ-MutLγ–dependent mechanism. Thus the question remains: Is the assembled, full-length SC structure required for MutSγ-MutLγ-dependent crossover formation in budding yeast?

Here we describe an interspecies complementation experiment that reveals intriguing features about the relationship between the full-length SC structure, the Zip1 transverse filament protein, and MutLγ-mediated crossover events in *S*. *cerevisiae*. We generated *S*. *cerevisiae* strains that express *Kluyveromyces lactis ZIP1* in place of *Saccharomyces cerevisiae ZIP1*, and observed that spores from *K*. *l*. *ZIP1*-expressing budding yeast display wild-type crossover levels despite a failure in SC formation. Our in-depth analysis of this separation-of-function version of Zip1 reveals several interesting findings. In particular, our study demonstrates that the full-length SC structure is dispensable for Zip-protein mediated and Mlh3-dependent crossover formation in budding yeast. Our data strongly suggest that crossover recombination activity, independent of SC elaboration, is sufficient to overcome a checkpoint-induced block to meiotic progression. Furthermore, we describe the surprising result that *K*. *lactis* Zip1 promotes crossovers in *S*. *cerevisiae* cells that are SC protein- and Mlh3-dependent, but largely independent of the MutSγ proteins Msh4 and Msh5. MutLγ activities are thus uncoupled from MutSγ in the context of *K*. *l*. *ZIP1*, suggesting that at least one aspect of *S*. *c*. Zip1 normally mediates a constraint that couples MutLγ-dependent resolvase activity to MutSγ-associated crossover intermediates. We discuss the idea that SC assembly itself could be involved in establishing such a constraint.

## Results

### *K*. *lactis* Zip1 rescues meiotic chromosome segregation functions of *S*. *cerevisiae* Zip1

**Fig 1:**
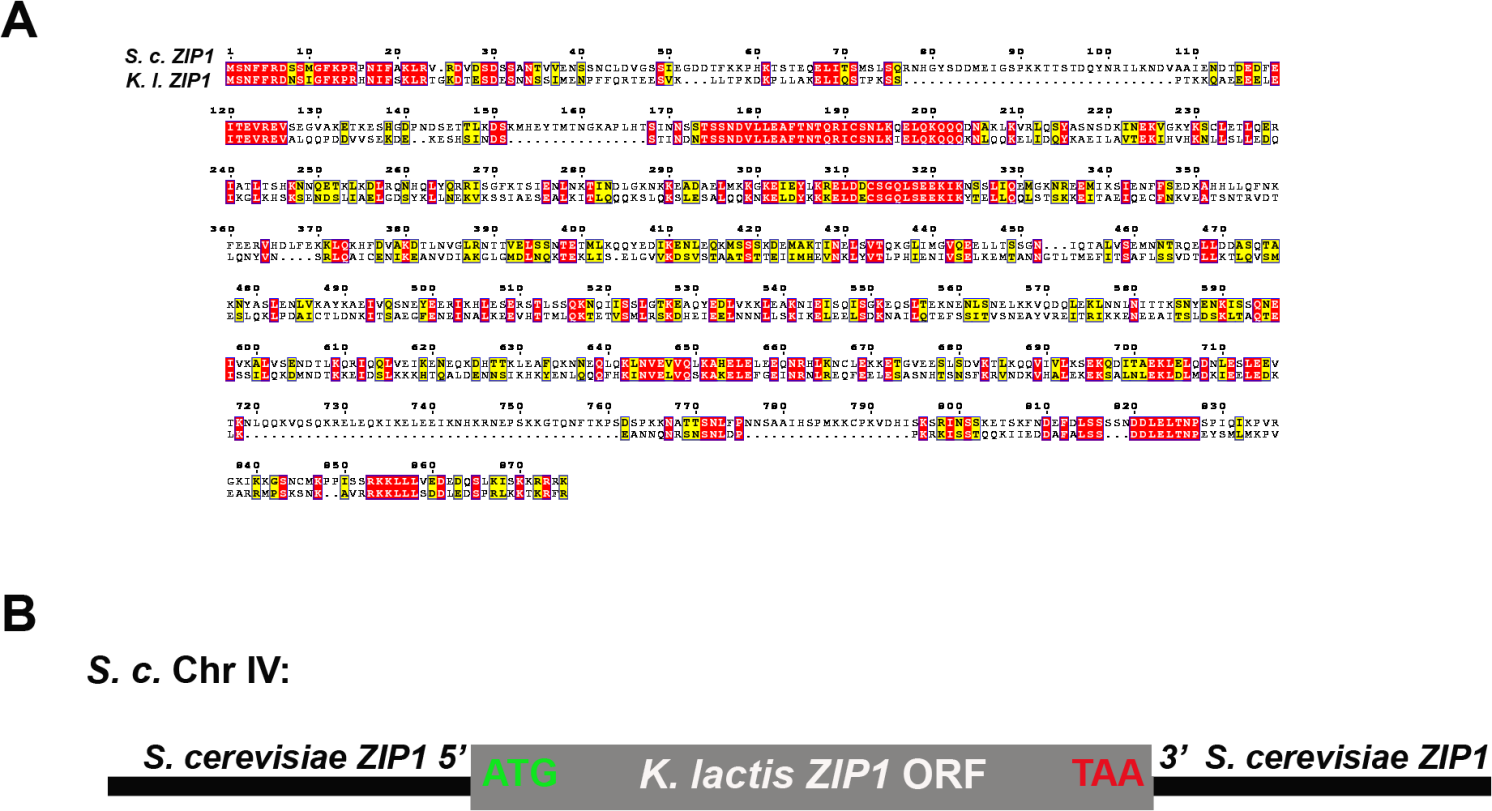
Alignment of *K. lactis* and *S. cerevisiae* Zip1 and experimental design. (A) *S*. *cerevisiae* and *K*. *lactis* Zip1 sequence alignment. ClustalW (http://www.ch.embnet.org) was used to align amino acid sequences for the translated products of *ZIP1* from the *S*. *cerevisiae* and *K*. *lactis* genomes, and the alignment was processed for presentation using MEGA tool [94]. The alignment suggests ~25% identity (red boxes), and ~16.7% homology (yellow boxes) between the two sequences. The approximate boundaries of an extended central region of the two proteins that is predicted to form coiled-coil are marked with blue arrow brackets (COILS program, [95]). (B) Cartoon depicting the chromosome IV genotype of *S*. *cerevisiae* strains expressing *K*. *l*. *ZIP1*. The diploid cells used in our study are homozygous for the locus illustrated in the cartoon. The start and stop codons of the *K*. *l*. *ZIP1* open reading frame are indicated in green and red, respectively.

*Kluyveromyces lactis ZIP1* encodes a protein that shares 25% identity and 16.7% homology with *S*. *cerevisiae*’s Zip1, and the two versions of Zip1 are predicted to share overall structural characteristics including an extended central coiled-coil domain flanked by non-coiled coil segments (Fig 1A). To determine whether *K*.*l*. Zip1 can rescue the meiotic functions of *S*. *c*. Zip1, we created an *S*. *cerevisiae* strain (CO9) in which the *S*. *c*. *ZIP1* ORF is replaced by the *K*. *l*. *ZIP1* ORF (Fig 1B).

The success of homologous chromosome segregation at meiosis I in budding yeast correlates with the viability of the haploid spore products formed. Accordingly, when we assessed spore viability among *S*. *cerevisiae* strains, greater than 90% of spores from meiotic cells carrying *S*. *c*. *ZIP1* (YAM1252) were viable while only 56% of spores were viable from diploids missing *ZIP1* (and therefore missing class I crossovers; Table 1). We found that 77% of spores from diploids expressing *K*. *l*. *ZIP1* were viable. Thus, *K*. *l*. Zip1 is able to promote successful meiotic chromosome segregation to some extent, even in an *S*. *cerevisiae* cell context.

**Table 1:** Sporulation efficiency and spore viability in *S*. *cerevisiae* cells expressing *S*. *c*. or *K. l. ZIP1*. Sporulation efficiency and viability of spores produced by strains expressing either *S*. *c*. *ZIP1* or *K*. *l*. *ZIP1*. Sporulation efficiency is the fraction of total sporulating cells that are 2, 3 or 4-spore asci. The far right column shows the overall spore viability of each strain. Displayed in each “Distribution of tetrad types” column is the frequency of tetrads containing four viable spores (4-sv), three viable spores (3-sv), two viable spores (2-sv), one viable spore (1-sv) or no viable spores (0-sv). Full strain genotypes are listed in S6 Table.

			Distribution of tetrad types (%)					
Strain	Sporulation efficiency % (n)	Tetrads dissected	4-sv	3-sv	2-sv	1-sv	0-sv	Spore viability %
***S*. *c*. *ZIP1***	43.7 (5561)	132	74	21	5	0	0	**92.4**
***zip1Δ***	5.1 (4503)	80	28	23	20	6	23	**55.9**
***K*. *l*. *ZIP1***	15.8 (7714)	239	50	23	16	5	6	**77.0**
*S*. *c*. *ZIP1/zip1Δ*	32.3 (4536)	172	69	19	11	1	0	89.1
*K*. *l*. *ZIP1/zip1Δ*	4.2 (4705)	110	30	28	21	10	11	64.1
*S*. *c*. *ZIP1/K*. *l*. *ZIP1*	42.8 (2002)	110	86	5	7	2	0	93.9
*ZIP1 pch2∆*	56.3 (3003)	110	75	18	6	0	1	91.4
*zip1∆ pch2∆*	45.6 (2034)	157	17	15	18	15	35	40.6
*K*.*l*. *ZIP1 pch2∆*	48.0 (4017)	160	28	18	21	13.1	20	54.8

*S*. *cerevisiae* cells expressing *K*. *l*. *ZIP1* as the sole source of Zip1 also display an intermediate sporulation efficiency. About half of sporulating diploid cells from wild-type *S*. *cerevisiae* of the BR1919-8B background progress to form spores (44% in the experiment shown in Table 1). Due to a Pch2-mediated checkpoint [57], only ~5% of sporulating diploids from *zip1* null *S*. *cerevisiae* strains form spores (Table 1). We found that *K*.*l*. *ZIP1*-expressing cells exhibit ~16% sporulation efficiency. *PCH2* removal from *K*. *l*. *ZIP1*-expressing *S*. *cerevisiae* cells resulted in a nearly wild-type (45%, n = 3002) sporulation efficiency, indicating that the Pch2-mediated prophase checkpoint is responsible for the diminished spore formation by *K*. *l*. *ZIP1*-expressing *S*. *cerevisiae* cells.

### *K*. *lactis* Zip1 localizes to meiotic chromosomes but fails to assemble synaptonemal complex in *S*. *cerevisiae*

We investigated the localization of *K*. *l*. Zip1 on *S*. *cerevisiae* meiotic chromosomes using antisera raised against *K*. *l*. Zip1 (kindly provided by Abby Dernburg) as well as antisera raised against *S*. *c*. Zip1 [10,58]. Both sets of antisera gave similar results, but because anti—*S*. *c*. Zip1 antisera gave a more robust and consistent signal, this latter antibody was used for the analyses presented here.

To assess the distribution of *S*. *c*. or *K*. *l*. Zip1 on meiotic prophase chromosomes, meiotic nuclei from *S*. *c*. cells expressing either *S*. *c*. *ZIP1* (control) or *K*. *l*. *ZIP1* were harvested at two-hour time points between 12 and 24 hours after transfer to sporulation medium, and surface-spread on glass slides for examination by immunofluorescence. Each strain expressed a single copy of *ECM11-MYC*; Ecm11 localizes uniformly along the length of the budding yeast SC central element substructure, and a fraction of Ecm11 protein in the SC is SUMOylated [12,13]. Strains were additionally missing *NDT80* activity, which is required for meiotic nuclei to progress beyond the pachytene stage of meiotic prophase [59]. Chromosome spreads from control and experimental nuclei were stained with anti-Zip1, anti-SUMO and anti-MYC antisera in order to assess whether SC structure is properly established.

**Fig 2:**
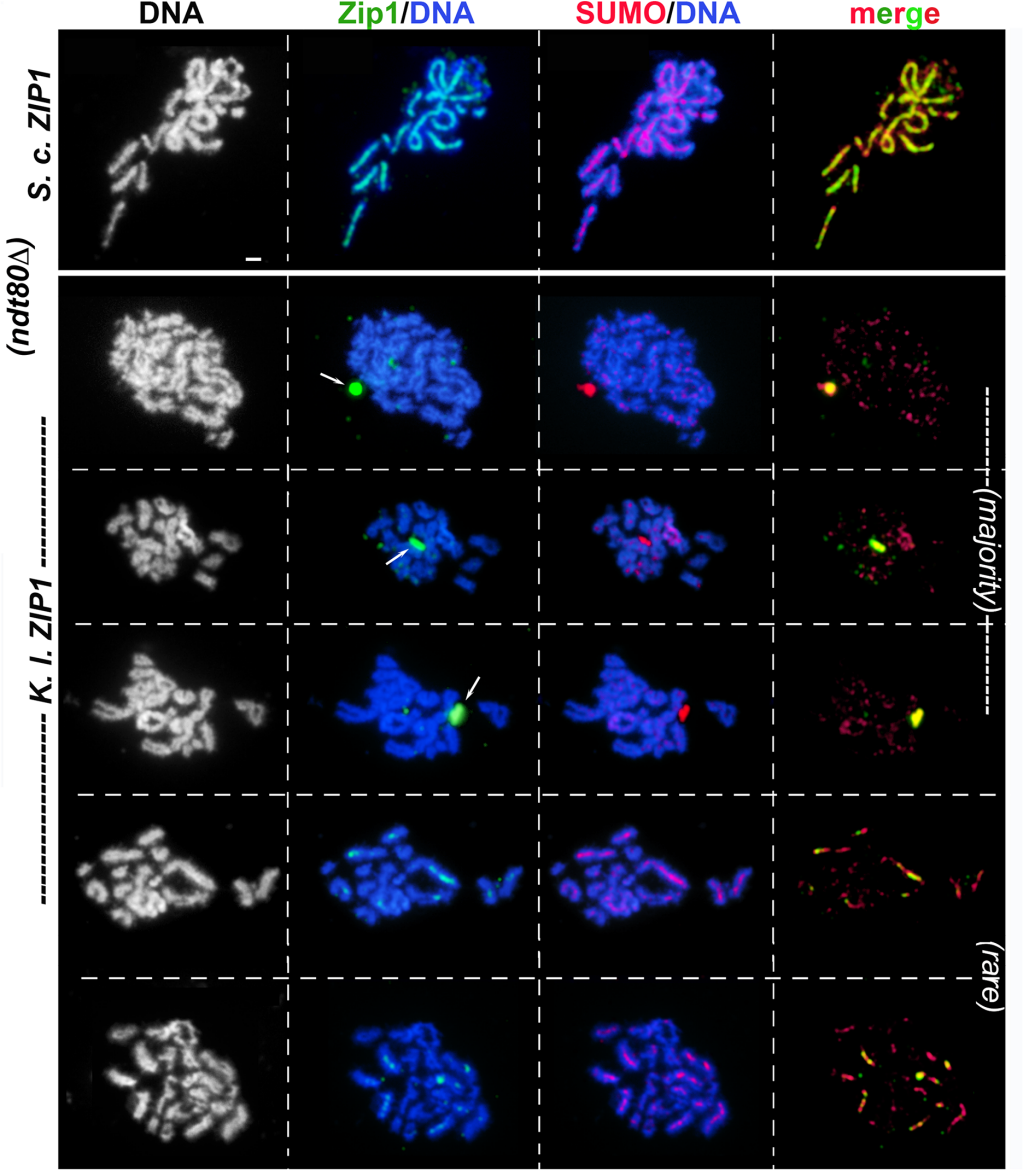
*K*. *lactis* Zip1 fails to assemble mature SC on *S*. *cerevisiae* meiotic chromosomes. *S*. *cerevisiae* meiotic cells expressing *S*. *c*. *ZIP1* (K375; top row) or *K*. *l*. *ZIP1* (YT12) were surface-spread at 2 hour intervals during sporulation, beginning at 12 hours after entry into sporulation medium and ending at 24 hours. These strains are homozygous for an *ndt80* null allele, and thus will not progress beyond the pachytene stage of meiotic prophase. Immunolocalization was used to label *S*. *c*. or *K*. *l*. Zip1 (green) and SUMO (red) on meiotic chromosomes (which are labeled with DAPI, white in first column and blue in second and third columns). Arrows point to polycomplex aggregates of Zip1. Sparse *K*. *l*. Zip1 foci and dotty SUMO staining was typically observed in *S*. *cerevisiae* cells expressing *K*. *l*. *ZIP1* (rows 2–4). Rarely (less than 6% of the total spreads, n = 560) SUMO assembled short linear structures, often overlapping a short linear stretch or several foci of K. l. Zip1 (rows 5 and 6). Scale, 1 micron.

**Fig 3:**
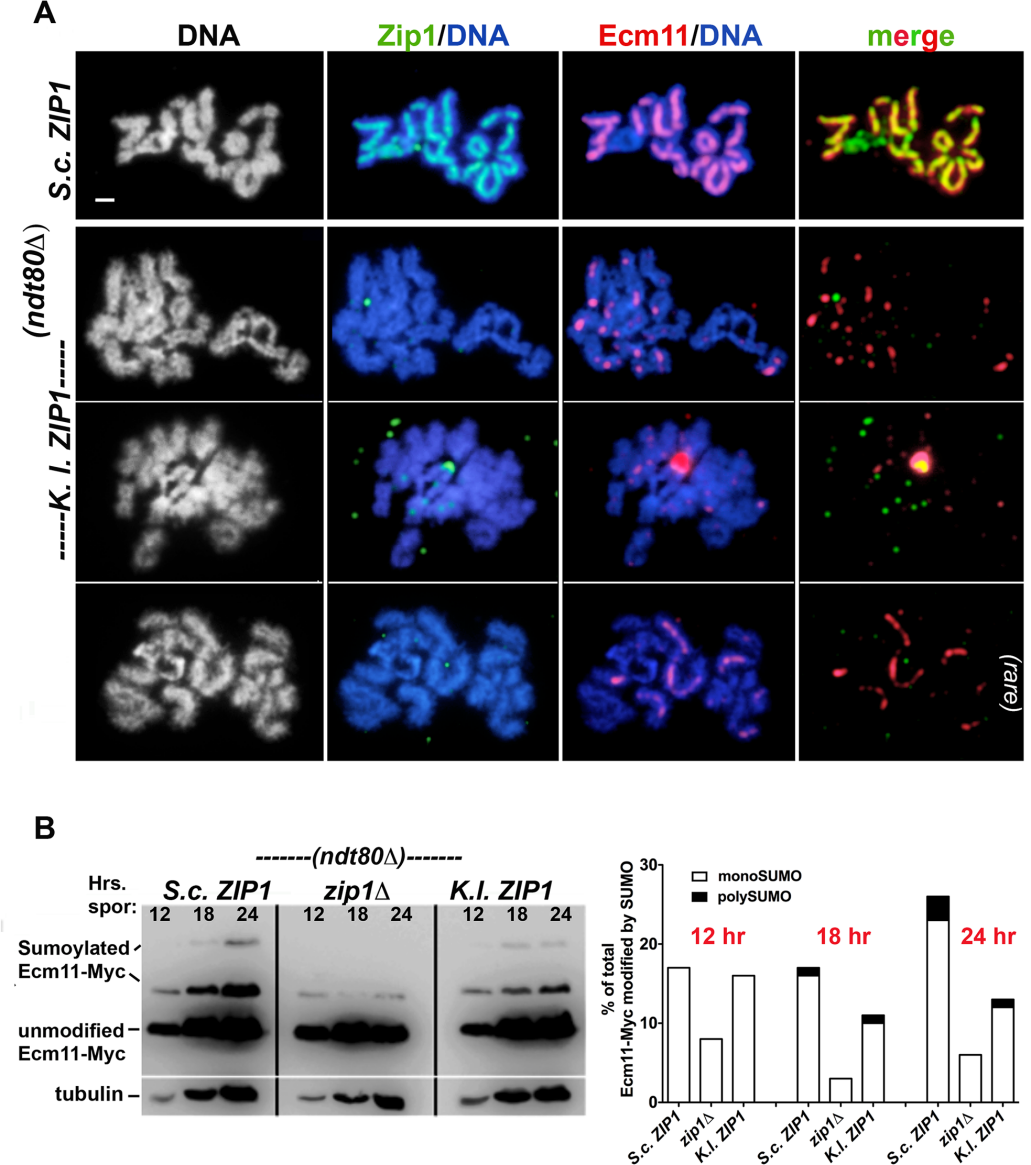
Ecm11-MYC predominantly assembles as foci on meiotic chromosomes and is partially SUMOylated in *S*. *cerevisiae* cells expressing *K*. *lactis ZIP1*. A) *S*. *cerevisiae* meiotic cells carrying one copy of *ECM11-MYC* and expressing *S*. *c*. *ZIP1* (K292; top row) or *K*. *l*. *ZIP1* (K268) were surface-spread at 2 hour intervals during sporulation, beginning at 12 hours after entry into sporulation medium and ending at 24 hours. These strains are homozygous for an *ndt80* null allele, and thus will not progress beyond the pachytene stage of meiotic prophase. Immunolocalization was used to label *S*. *c*. or *K*. *l*. Zip1 (green) and Ecm11-MYC (red) on meiotic chromosomes (labeled with DAPI, white in first column and blue in second and third columns). Sparse *K*. *l*. Zip1 foci and dotty Ecm11-MYC staining was typically observed in *S*. *cerevisiae* cells expressing *K*. *l*. *ZIP1* (rows 2–3). Similar to our observations of SUMO localization on meiotic chromosomes in *S*. *cerevisiae* cells expressing *K*. *l*. *ZIP1*, Ecm11-MYC occasionally (less than 6% of the total spreads) assembled short linear structures. Scale, 1 micron. (B) Trichloroacetic acid (TCA) extracts from sporulating cultures of *S*. *cerevisiae* homozygous for *ECM11-MYC*, and carrying *S*. *c*. *ZIP1* (AM2712), a *zip1* null allele (AM2784), or *K*. *l*. *ZIP1* (AM2711) were run on a 10% polyacrylamide gel and western blotting was used to detect unSUMOylated, monoSUMOylated and polySUMOylated forms of Ecm11-MYC, as described in [12,13]. These strains are homozygous for an *ndt80* null allele, and thus will not progress beyond the pachytene stage of meiotic prophase. For each strain, the fraction of total Ecm11-MYC found in the mono-SUMOylated (open bar) and poly-SUMOylated (shaded bar) forms at three sporulation time points is plotted in the graph below.

**Fig 4:**
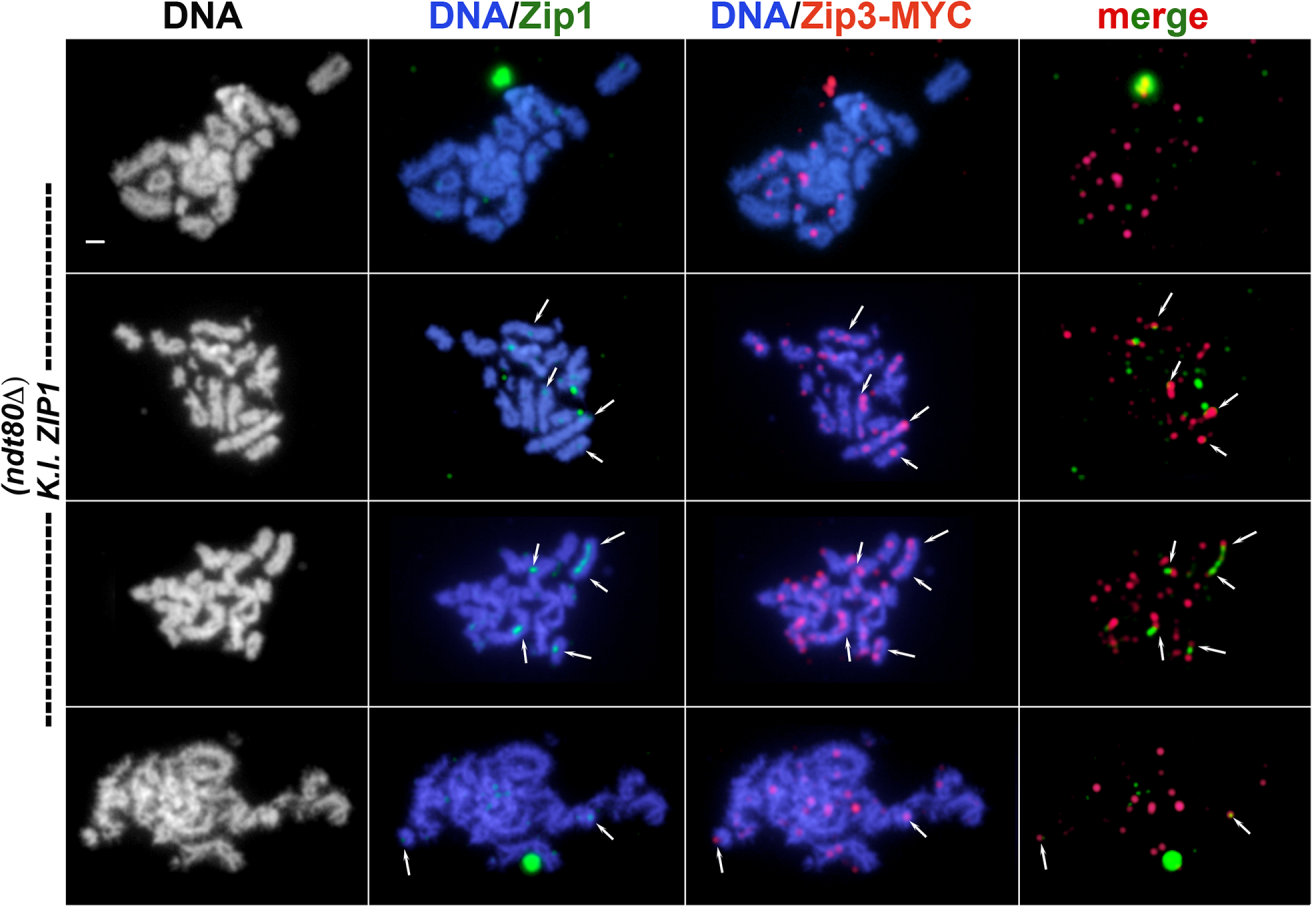
Relative distribution of Zip3-MYC and *K*. *l*. Zip1 on meiotic chromosomes in *K*. *l*. *ZIP1*-expressing cells. *S*. *cerevisiae* meiotic cells carrying one copy of *ZIP3-MYC* and expressing *K*. *l*. *ZIP1* (K375) were surface-spread at 2 hour intervals during sporulation, beginning at 12 hours after entry into sporulation medium and ending at 24 hours. These strains are homozygous for an *ndt80* null allele, and thus will not progress beyond the pachytene stage of meiotic prophase. Immunofluorescence was used to label *K*. *l*. Zip1 (green) and Zip3-MYC (red) on meiotic chromosomes (labeled with DAPI, white in first column and blue in second and third columns). Note in the top row images, the polycomplex aggregate of *K*. *l*. Zip1 overlaps an aggregate of Zip3-MYC. Arrows point to a subset of apparent co-localization or adjacency events between *K*. *l*. Zip1 foci and Zip3-MYC. Scale, 1 micron.

At the 22 and 24 hour time points, a majority (> 80%) of chromosome spreads from *ndt80Δ* mutants appeared to be at the pachytene stage where homologous chromosomes are aligned and exhibit nearly full synapsis (Figs 2 and 3A). The DAPI-stained DNA morphology of surface-spread pachytene chromosomes from wild-type *S*. *cerevisiae* reveals distinct, individualized chromosome pairs with Zip1, Ecm11-MYC, and SUMO coinciding as linear structures at the interface of each chromosome pair (Figs 2 and 3A, [11–13]). In contrast, while the DAPI-stained DNA morphology of our *K*.*l*. *ZIP1* meiotic time course nuclei suggested normal progression into the pachytene stage, none of the meiotic chromosome spreads from cells expressing *K*. *l*. *ZIP1* at any of the seven time points (n = 560) exhibited full-length linear structures of Zip1, SUMO, or Ecm11-MYC. Across time points, the vast majority of nuclei displayed either no detectable *K*. *l*. Zip1 or a handful of *K*. *l*. Zip1 foci dispersed along meiotic chromosomes (Figs 2 and 3), accompanied by a punctate distribution of SUMO and Ecm11 on chromosomes. The number of *K*. *l*. Zip1 chromosome-associated foci exhibited by these nuclei ranged from 1–36, with an average of 11 *K*. *l*. Zip1 foci per nucleus. The most prominent *K*.*l*. Zip1 structure found associated with *S*. *cerevisiae* meiotic nuclei was an aggregate of *K*. *l*. Zip1, Ecm11-MYC and SUMO proteins (examples in Figs 2 and 3A and S1). *K*. *l*. Zip1 polycomplexes also contained the SIC protein, Zip3 (Fig 4). Such “polycomplex” aggregates of synapsis proteins are a characteristic feature of meiotic nuclei in *S*. *cerevisiae* mutants that fail to assemble SC [12,15,18,20]. *K*. *l*. Zip1 polycomplexes were exhibited by over half (358/560) of the surface-spread nuclei, and were observed at both early and later meiotic time points, regardless of whether they displayed detectable chromosomal *K*. *l*. Zip1 foci.

An additional Zip1 staining pattern was rarely observed, in which a single or a small number of short linear Zip1 structures appear on chromosomes (examples in Figs 2 and 3). Such short linear structures may result from *bona fide* but aborted elaborations of an SC precursor, or could be the result of several *K*. *l*. Zip1 foci assembled side-by-side on the chromosome. Interestingly, especially in those nuclei that showed robust Zip1 foci or short linear stretches, Ecm11 and SUMO often appeared as short linear assemblies that encompass but surpass the Zip1 structures in length (Figs 2 and 3). Short linear Zip1, Ecm11 and/or SUMO assemblies were rarely found in any nuclei among all time points examined, indicating that these structures are not stable; we observed an apparently linear Zip1, Ecm11 or SUMO structure in 0/75 nuclei at 12 hours, 3/91 nuclei at 14 hours, 4/92 nuclei at 16 hours, 10/85 nuclei at 18 hours, 6/89 nuclei at 20 hours, 1/84 nuclei at 22 hours and 3/44 nuclei at 24 hours. Taken together, our data for three readouts of SC structure (Zip1, Ecm11, SUMO), across a 12-hour meiotic prophase time course, indicate that *K*. *l*. Zip1 fails to assemble mature SC in *S*. *cerevisiae* cells.

To guard against the possibility that our antibody recognizes only a subset of potentially detectable *K*. *l*. Zip1 protein, we examined the distribution of an epitope-tagged version of *K*. *l*. Zip1, which retains function. Insertion of a V5 epitope tag just after asparagine at position 647 in the *K*. *l*. Zip1 protein failed to rescue the spore viability defect of *zip1* null *S*. *cerevisiae* cells, despite the fact that YFP, inserted at the equivalent position (amino acid 700) of *S*. *c*. Zip1, creates a functional *S*. *c*. Zip1-YFP protein [60]. However, insertion of the V5 epitope tag just after arginine at position 472 generated a *K*. *l*. Zip1 protein that rescues the spore viability defect of *S*. *c*. *zip1* null diploids to the same extent as untagged *K*. *l*. Zip1: Diploids carrying the *K*. *l*. *ZIP1-V5* cassette in place of the *S*. *c*. *ZIP1* ORF exhibited 20.6% sporulation efficiency and the spore products exhibited 79.9% viability (307 viable out of 384 spores dissected). Immunolocalization of the V5 tag in *S*. *cerevisiae* meiotic nuclei expressing *K*. *l*. *ZIP1-V5* in conjunction with *ECM11-MYC* revealed a distribution of *K*. *l*. Zip1 on *S*. *cerevisiae* meiotic chromosomes indistinguishable from that observed using anti-Zip1 antisera (S1 Fig). Importantly, linear V5 structures were never observed among the meiotic pachytene nuclei we screened. Instead, V5 staining most often appeared as a small polycomplex structure, which typically contained Ecm11-MYC (S1 Fig). Occasionally, meiotic chromosomes displayed a limited number of faint *K*. *l*. Zip1-V5 foci on chromosomes; these V5 foci often, but not always, co-localized with Ecm11-MYC foci.

**Fig 5:**
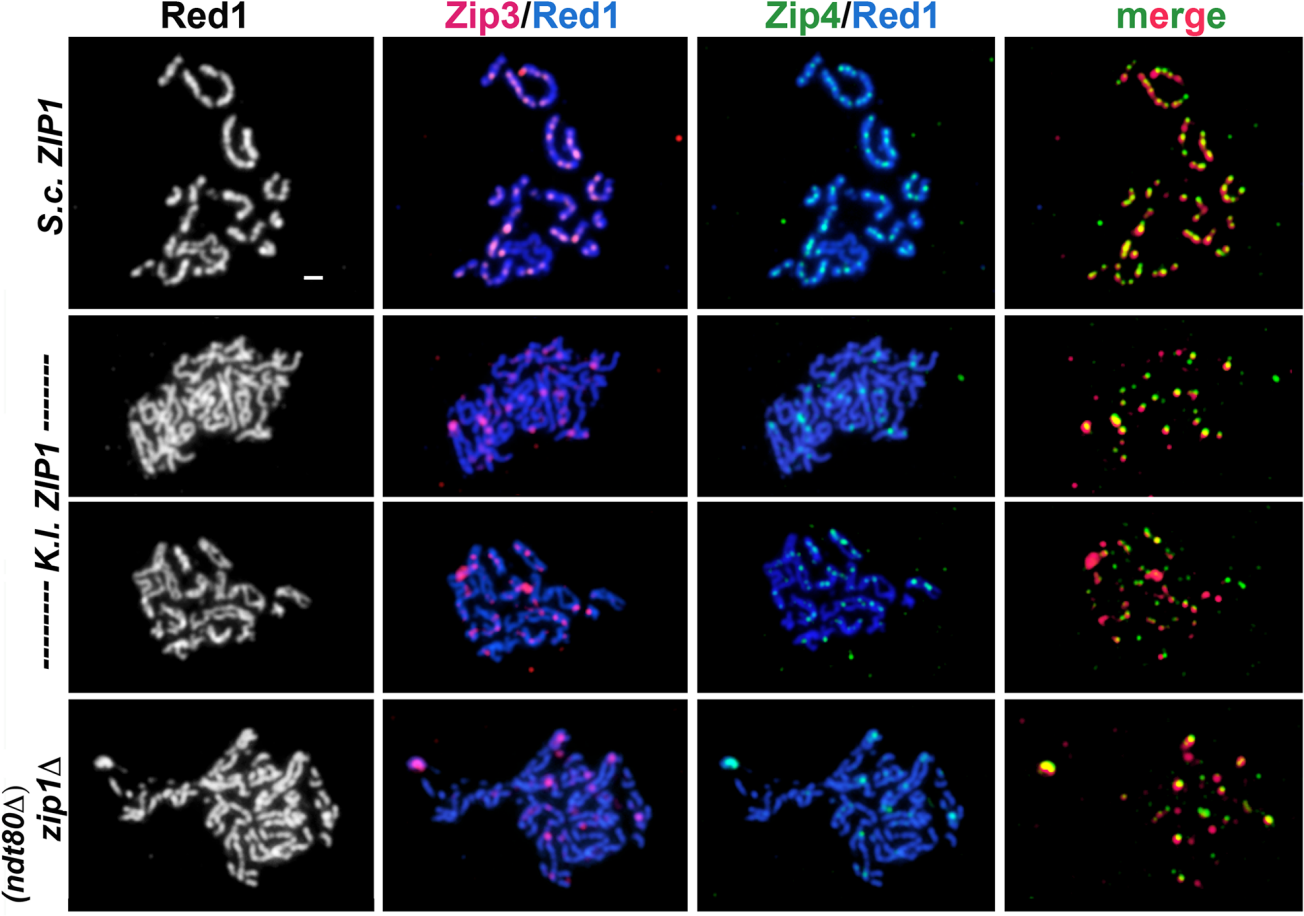
Zip3-MYC and Zip4-HA levels exhibited by *K*. *l*. *ZIP1*-expressing cells resemble *zip1* null levels. Sporulating cultures of *S*. *cerevisiae* meiotic cells carrying one copy of *ZIP3-MYC* and *ZIP4-HA* and expressing *S*. *c*. *ZIP1* (AM3362), a *zip1* null allele (AM3363) or *K*. *l*. *ZIP1* (AM3361) were surface-spread on glass slides at 24 hours. AM3362 and AM3361 strains are homozygous for an *ndt80* null allele, and thus will not progress beyond the pachytene stage of meiotic prophase. AM3363 sporulating cultures are enriched for pachytene owing to the fact that *zip1*null meiotic cells trigger the meiotic prophase checkpoint. Immunolocalization was used to label the chromosomal axis protein Red1 (white, blue), Zip3-MYC (red) and Zip4-HA (green) on surface-spread meiotic chromosomes. See (S2 Fig) for quantification of Zip3-MYC and Zip4-HA foci and frequency of co-localization. Scale, 1 micron.

Further support for the conclusion that *K*. *l*. Zip1 fails to assemble SC in *S*. *cerevisiae* came from staining of the axial element protein, Red1, on surface-spread meiotic chromosomes. Red1 labels the axes of meiotic prophase chromosomes [61]; because the SC structure brings homolog axes into intimate alignment along their entire lengths, the closely apposed Red1-labeled axes of partner homologs in wild-type meiotic pachytene bivalents appear as a single linear structure along their full-lengths (Fig 5, top left). In contrast, meiotic pachytene chromosomes from *zip1* null cells exhibit loosely-associated chromosome axes labeled by Red1 (Fig 5, bottom left) [10]. The Red1-labeled “loops” apparent in such synapsis-defective mutants correspond to homolog axes joined in intimate alignment only at sporadic positions along the chromosomes (these “axial associations” are presumably where a crossover event has been established) [62]. The Red1-stained chromosome axis patterns exhibited by surface-spread meiotic chromosomes from *K*. *l*. *ZIP1*-expressing *S*. *cerevisiae* cells appeared indistinguishable from those seen in *zip1* null cells, consistent with an absence of mature SC structure (Fig 5, middle left).

### The relationship between *K*. *lactis* Zip1 and *S*. *cerevisiae* synapsis proteins

As described above, proteins that appear to have a structural role in building SC (such as Ecm11 and SUMO) fail to assemble normal linear structures in *S*. *cerevisiae* cells expressing *K*. *l*. *ZIP1*. However, evidence that *K*. *l*. Zip1 is able to interface, at least to some extent, with components of the SC in *S*. *cerevisiae* cells was revealed by an examination of SUMOylated forms of Ecm11-MYC in wild-type, *zip1* null, and *K*. *l*. *ZIP1*-expressing cells. Humphryes et al. reported that the SUMOylation of Ecm11-MYC during meiosis is largely dependent on Zip1 [12]. Consistent with their report, we found that levels of mono- and poly-SUMOylated forms of Ecm11-MYC were severely diminished in meiotic cell extracts from *zip1* null cells, relative to wild-type meiotic cell extracts (Fig 3B). In meiotic cell extracts from *S*. *cerevisiae* cells expressing *K*. *l*. *ZIP1*, mono- and poly-SUMOylated Ecm11-MYC rose to near wild-type levels between the 0 and 12 hour time points, and appeared intermediate between wild-type and the *zip1* null at the 18 and 24 hour time points (Fig 3B). These data demonstrate that *K*. *l*. Zip1 can support partial levels of Ecm11-MYC SUMOylation in *S*. *cerevisiae* meiotic cells. *K*. *l*. Zip1 might promote the SUMOylation of Ecm11 within complexes on chromosomes and/or within the polycomplex structure (where *K*. *l*. Zip1, Ecm11-MYC, and SUMO co-localize) [11,12].

We also examined the distribution of SIC proteins on meiotic chromosomes in *K*.*l*. *ZIP1*-expressing cells. SIC proteins, such as Zip2, Zip3 and Zip4, are required for SC assembly, but localize as multiple foci along the length of SCs instead of displaying a linear, Zip1-like distribution ([14–16,18] and Figs 4 and 5). We first examined Zip3-MYC and Zip1 on surface-spread meiotic chromosomes from *S*. *cerevisiae* cells expressing *K*. *l*. *ZIP1* (Fig 4). Nuclei were harvested every two hours from 12 to 24 hours after transfer to sporulation medium. At each time point, the number of Zip3-MYC foci on surface-spread chromosomes from *K*.*l*. *ZIP1* expressing cells ranged from ~10–40, which is diminished relative to the range of foci (50–70) observed on wild-type pachytene chromosomes (S2 Fig). A fraction of *K*. *l*. Zip1 foci in each nucleus (arrows in Fig 4) appeared to overlap or localize adjacent to a Zip3-MYC focus. Taking nuclei from all time points into account, 50% (1059/2108, n = 184 nuclei) of Zip1 foci overlapped or localized adjacent to a Zip3-MYC focus. However, the low number of Zip1 relative to Zip3-MYC foci exhibited by each nucleus prevents a rigorous assessment of whether the apparent adjacency events are significantly different from what one would observe from a random distribution of Zip3-MYC and *K*. *l*. Zip1.

Next we analyzed the co-localization of Zip3-MYC and Zip4-HA on pachytene stage meiotic chromosomes from *S*. *cerevisiae* cells expressing *S*. *c*. *ZIP1* or *K*.*l*. *ZIP1*, or in cells missing *ZIP1* altogether (Fig 5). As has been previously reported [14–16,18,58], we observed that the number of Zip3 and Zip4 foci on wild-type pachytene chromosomes ranged between 50–70, and well over 90% of Zip3 and Zip4 foci co-localize with one another (Figs 5 and S2). The number of Zip3-MYC and Zip4-HA foci observed on pachytene chromosomes from *zip1* null cells was diminished, relative to wild type, ranging from 8–31 with an average of 19 +/- 0.95 Zip3-MYC and from 4–35 with an average of 16 +/- 1.00 Zip4-HA foci per nucleus (n = 39 nuclei). This observation is in contrast to a prior report stating that normal numbers of Zip3 foci are observed on meiotic chromosomes in *zip1* null cells [15] but is consistent with the lower number of Zip3 foci that were observed on meiotic pachytene chromosomes from *zip1* null cells in other studies [63,64]. The diminished number of SIC foci on chromosomes from *zip1* null cells indicates a role for the SC or Zip1 in either the formation or persistence of SIC complexes during meiotic prophase.

As previously reported [18], the co-localization between Zip3-MYC and Zip4-HA on meiotic chromosomes from *zip1* null strains is high, although in our experiments not as high as that observed on wild-type pachytene chromosomes (S2B Fig). In the 39 *zip1* null pachytene chromosome spreads examined, the number of Zip3-Zip4 coincident localization events ranged from 38%-100% with an average of 70 +/- 3% (S2 Fig).

We found that the number of Zip3-MYC and Zip4-HA foci observed on surface-spread meiotic chromosomes from cells expressing *K*. *l*. Zip1 resembled the levels observed on pachytene-stage chromosomes from *zip1* null cells. We counted between 5–40, with a mean of 16 +/- 0.83 Zip3-MYC foci, and between 3–35, with a mean of 15 +/- 0.90 Zip4-HA foci on meiotic pachytene stage chromosomes from *K*. *l*. *ZIP1*-expressing cells (n = 47). Apparent co-localization events observed between Zip3-MYC and Zip4-HA on meiotic chromosomes from *S*. *cerevisiae* expressing *K*. *l*. Zip1 ranged from 33%-100% with an average of 63 +/- 3% (S2B Fig). The percent Zip3-Zip4 co-localization values for *zip1* null and for *K*. *l*. *ZIP1*-expressing cells are not significantly different from one another, as evaluated by an unpaired t test using Welch’s correction (two-tailed P = 0.1). Our data indicate that expression of *K*. *l*. *ZIP1* is not sufficient to restore a wild-type number of cytologically-detectable SIC foci to pachytene chromosomes in *S*. *cerevisiae* meiotic cells missing *S*. *c*. *ZIP1*.

### *K*. *lactis* Zip1 provides partial function at centromeres

Zip1 has been found to associate with the centromere regions of meiotic prophase chromosomes and centromeres mark sites where many of the earliest SC assembly events occur in *S*. *cerevisiae* [63,65]. Furthermore, *S*. *c*. Zip1 promotes pairwise associations between centromeres outside of the context of the SC, during early and late meiotic prophase [65–67]. To investigate whether *K*. *l*. Zip1 plays a role at centromeres in *S*. *cerevisiae* meiotic nuclei, we monitored an epitope-tagged version of the kinetochore protein, Ctf19-MYC, which localizes to the centromere regions on meiotic prophase chromosomes [65,68].

In order to assess co-localization between *K*. *l*. Zip1 and meiotic centromeres, we harvested sporulating cells at two-hour intervals that spanned 12 to 24 hours following transfer to sporulation medium. Across all time points, surface-spread meiotic chromosomes from cells expressing *K*. *l*. *ZIP1* and *CTF19-MYC* exhibited an average of 10 *K*. *l*. Zip1 foci and 22 Ctf19-MYC foci (n = 120 nuclei). Despite the fact that centromere foci typically far outnumbered detectable *K*.*l*. Zip1 foci, *K*. *l*. Zip1 foci appeared co-localized or adjacent to Ctf19-MYC foci only 46% of the time (539/1163 Zip1 foci) (S3 Fig). From an analysis of exclusively pachytene stage nuclei (classified based on DAPI-stained DNA morphology) we measured an average of eight *K*. *l*. Zip1 foci and 21 Ctf19-MYC foci (n = 68 nuclei); in this subgroup, *K*. *l*. Zip1 foci appeared co-localized or adjacent to Ctf19-MYC foci 59% of the time (325/555 Zip1 foci). Thus, while *K*. *l*. Zip1 and centromeres do not exhibit a strong co-localization pattern, these data do not rule out the possibility that *K*. *l*. Zip1 may have some preferential affinity for centromere sites on *S*. *cerevisiae* meiotic chromosomes.

We additionally explored the relationship between *K*. *l*. Zip1 and centromeres in *S*. *cerevisiae* meiotic cells through a functional assay. Zip1 facilitates two-by-two associations between meiotic prophase centromeres, independent of SC formation [65–67]. For example, *spo11* mutant meiotic cells fail to initiate recombination and also fail to assemble SC, but centromeres nevertheless tend to associate in pairs. Thus, surface-spread meiotic prophase nuclei from *spo11* strains exhibit fewer than 32, and often an average of 16, centromere groups. In contrast, surface spread nuclei from *spo11* meiotic cells that are also missing *ZIP1* exhibit closer to 32 centromere foci, demonstrating that Zip1 is required for the observed Spo11-independent centromere associations. Zip1-dependent centromere associations can also be observed outside of the context of SC, in haploid cells capable of entry into meiosis. In the haploid cell context, Zip1-dependent centromere associations are found in both *spo11* null and *SPO11* contexts (neither of which supports extensive SC formation); the mechanisms used for Zip1-dependent centromere associations in *spo11* null versus *SPO11* cells may involve distinct (yet overlapping) mechanisms since only the latter is dependent on the Pph3 phosphatase [65–67].

We assessed the capacity of *K*. *l*. Zip1 to facilitate centromere associations in both diploid and haploid *spo11* null meiotic cells as well as in *SPO11* haploid meiotic cells expressing *CTF19-MYC*. Haploids capable of progressing through meiotic prophase were created by targeting a *MAT* **a** locus cassette to an ectopic location in the genome (the *THR1* locus) in *MATα* haploids [69]. We compared the number of Ctf19-MYC foci observed on surface-spread meiotic chromosomes when such strains carried *S*. *c*. *ZIP1*, *K*. *l*. *ZIP1*, or the *zip1* null genotype.

Consistent with the observations described in the initial report on “centromere coupling” [65], *spo11* diploid meiotic cells expressing *S*. *c*. *ZIP1* exhibited a variable number of Ctf19-MYC foci per nucleus, ranging from 4–27 with an average of 17 (n = 245 total nuclei over 5 experiments; S4 Fig), while *spo11* haploid meiotic cells exhibited from 4–14, with an average of 9 Ctf19-MYC foci per nucleus (n = 143 total nuclei over 3 experiments). In contrast, *spo11* diploid cells missing *ZIP1* exhibited between 16–35 with an average of 26 Ctf19-MYC foci (n = 258 total nuclei over 5 experiments), and *spo11* haploid cells missing *ZIP1* exhibited between 9–22 with an average of 15 Ctf19-MYC foci (n = 168 total nuclei over 3 experiments; S4 Fig).

In *spo11* diploid meiotic cells expressing *K*. *l*. *ZIP1*, we observed between 5–36 with an average of 20 Ctf19-MYC foci (n = 288 total nuclei over 5 experiments, S4 Fig), suggesting that *K*. *l*. Zip1 may weakly restore the centromere association function of *S*. *c*. Zip1 in the context of a diploid *spo11* cell. In contrast, however, *spo11* null haploid meiotic cells expressing *K*. *l*. *ZIP1* displayed no capacity for centromere association: *spo11* null haploid meiotic cells expressing *K*. *l*. *ZIP1* exhibited an average of 15 Ctf19-MYC foci (n = 152 total nuclei over 3 experiments).

As reported in [66], *SPO11* haploid meiotic cells exhibited between 6–12 with an average of 8 Ctf19-MYC foci, while *SPO11 zip1* null haploid meiotic cells exhibited between 8–22 with an average of 14 Ctf19-MYC foci. We found that *SPO11 K*. *l*. *ZIP1*-expressing haploid meiotic cells exhibited between 7–22 with an average of 14 Ctf19-MYC foci.

Taken together, our findings suggest that while *K*. *l*. Zip1 may maintain a weak capacity to mediate *SPO11*-independent centromere associations in diploid *spo11* meiotic cells, *K*. *l*. Zip1 fails to facilitate persistent centromere associations in a haploid meiotic cell context (with or without Spo11 activity). The basis for the difference observed between diploid and haploid cell contexts may reflect a sensitivity (on the part of centromeres) to the dosage of *K*. *l*. Zip1.

### *K*. *lactis* Zip1 promotes a wild-type level of crossing over in a subset of *S*. *cerevisiae* meiotic cells

**Fig 6:**
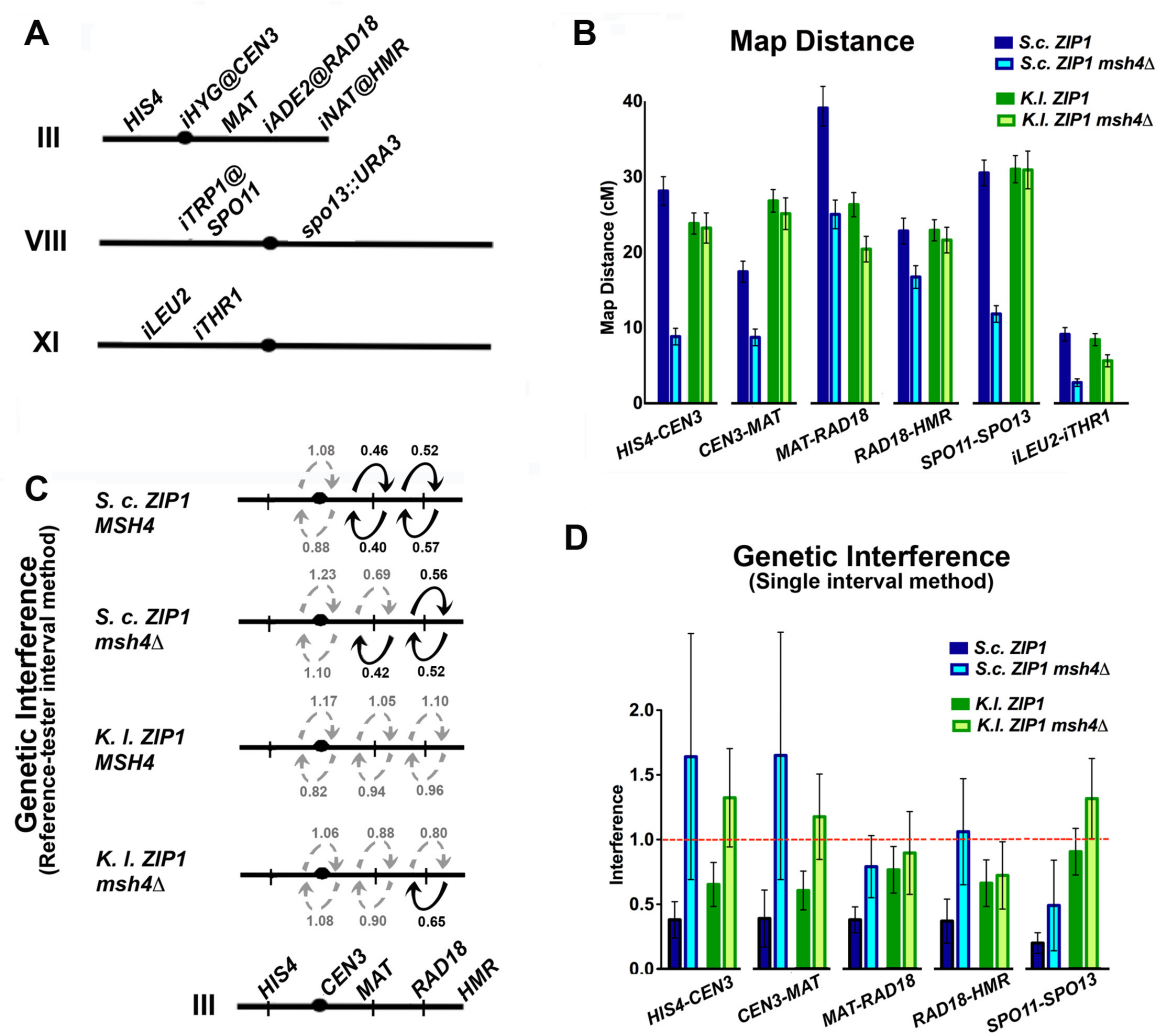
Four-spore viable tetrads from *K*. *l*. *ZIP1* meioses exhibit wild-type crossover levels, largely independent of Msh4, with diminished interference. Cartoon in (A) displays the markers used to define six genetic intervals in which crossing over was assessed in spores from *S*. *c*. *ZIP1* and *K*. *l*. *ZIP1*-expressing *S*. *cerevisiae* strains (YT131, YT125, AM3313 and YT152). Graph in (B) plots the map distances (+/- S. E.) for each of the six intervals (labeled on the *x* axis) that were calculated from linkage analysis in 4-spore viable tetrads from each of the four strains analyzed (*S*. *c*. *ZIP1 +/- MSH4* are indicated in darker and lighter blue, respectively while *K*. *l*. *ZIP1* +/- *MSH4* are indicated in darker and lighter green.). The specific values for map distance are listed in Table 2. Cartoon in (C) depicts observable (solid dark arrow) or undetectable (gray, dotted arrow) interference acting between adjacent genetic intervals on chromosome III, as measured by the “interference ratio” method (see Text for details) [74,75]. Strain genotypes are indicated at left. The interference ratio gives an estimate of the strength of interference; *P* values from chi-square analysis of the distribution of tetrad types derived from recombinant versus non-recombinant groups (Instat, Graphpad.com; S5 Table), as well as statistical analysis of the significance of differences between map lengths calculated by tetrad types (Stahl Online Tools; S5 Table) were used to determine whether adjacent intervals exhibited interference. Graph in (D) shows the genetic interference values obtained for each genetic interval (labeled on the *x* axis) when the number of four-chromatid double crossovers observed (NPDs) are compared to the number expected if there was no interference [76]. The red line marks an interference value of 1, which is equal to an absence of positive interference. The specific values for both map distance and interference measured in this manner are listed in Table 2.

Since crossover recombination events are critical for the formation of the stable connections between homologs that ensure proper chromosome disjunction at meiosis I, it is reasonable to speculate that the basis for the diminished viability of spore products from *K*. *l*. Zip1-expressing *S*. *cerevisiae* strains lies in a failure of *K*. *l*. Zip1 to rescue *S*. *c*. Zip1’s crossover function. We therefore assessed crossover formation in four consecutive intervals on chromosome III, one interval on chromosome VIII and one interval on chromosome XI in *S*. *cerevisiae* cells expressing *K*. *l*. *ZIP1* (Fig 6B and Table 2).

**Table 2:** Map distances from 4 spore-viable tetrads carrying *S*. *cerevisiae* or *K*. *lactis ZIP1*. Map distances were calculated using tetrad analysis (as indicated in Methods), in *S*. *c*. *ZIP1*- expressing and *K*. *l*. *ZIP1*-expressing strains (YT131, YT125, AM3313 and YT152). Tetrad analysis to generate genetic distances, interference (and standard error (S.E.) values using tetrad data were calculated using the Stahl lab online tools: http://molbio.uoregon.edu/~fstahl/. Interference was measured by calculating the ratio of Non Parental Ditype tetrads (NPDs) observed over the NPDs expected (values less than one reflect positive interference). NPDs expected for each interval were calculated using the fraction of tetratypes (TT) observed in each dataset according to the formula: NPD^exp^ = 1/2(1-*f*TT-[1-*3fTT*/2]^2/3^ (where *f*TT = fraction of tetratypes) [76].

Strain	Interval (chromosome)	PD	TT	NPD	Total	cM	%WT	NPD^obs^/NPD^exp^ (+/- SE)
*S*.*c*. *ZIP1 MSH4*	*HIS4-CEN3 (III)*	257	231	8	496	**28.1**	**100**	0.38 (0.14)
	*CEN3-MAT (III)*	340	155	3	498	**17.4**	**100**	0.39 (0.22)*
	*MAT-RAD18 (III)*	187	288	16	491	**39.1**	**100**	0.38 (0.11)
	*RAD18-HMR (III)*	295	196	5	496	**22.8**	**100**	0.37 (0.17)
	*SPO11-SPO13 (VIII)*	219	260	6	485	**30.5**	**100**	0.20 (0.08)
	*iLEU2- iTHR1 (XI)*	403	90	0	493	**9.1**	**100**	n.d.*
*S*.*c*. *ZIP1 msh4Δ*	*HIS4-CEN3 (III)*	521	90	3	614	**8.8**	**31**	1.64 (0.95)*
	*CEN3-MAT (III)*	526	90	3	619	**8.7**	**50**	1.65 (0.96)*
	*MAT-RAD18 (III)*	362	230	12	604	**25**	**64**	0.79 (0.24)
	*RAD18-HMR (III)*	441	162	7	610	**16.7**	**73**	1.1 (0.41)
	*SPO11-SPO13 (VIII)*	465	129	2	596	**11.8**	**39**	0.49 (0.35)*
	*iLEU2- iTHR1 (XI)*	587	33	0	620	**2.7**	**30**	n.d.*
*K*.*l*. *ZIP1 MSH4*	*HIS4-CEN3 (III)*	561	351	15	927	**23.8**	**85**	0.65 (0.17)
	*CEN3-MAT (III)*	525	393	18	936	**26.8**	**154**	0.60 (0.15)
	*MAT-RAD18 (III)*	519	355	19	893	**26.3**	**67**	0.76 (0.18)
	*RAD18-HMR (III)*	570	337	14	921	**22.9**	**100**	0.66 (0.18)
	*SPO11-SPO13 (VIII)*	497	401	29	927	**31**	**102**	0.90 (0.18)
	*iLEU2- iTHR1 (XI)*	793	140	3	936	**8.4**	**92**	1.03 (0.60)*
*K*.*l*. *ZIP1 msh4Δ*	*HIS4-CEN3 (III)*	368	184	13	565	**23.2**	**83**	1.32 (0.38)
	*CEN3-MAT (III)*	352	200	14	566	**25.1**	**144**	1.17 (0.33)
	*MAT-RAD18 (III)*	363	174	8	545	**20.4**	**52**	0.89 (0.32)
	*RAD18-HMR (III)*	358	193	8	559	**21.6**	**95**	0.72 (0.26)
	*SPO11-SPO13 (VIII)*	308	216	20	544	**30.9**	**101**	1.31 (0.31)
	*iLEU2- iTHR1 (XI)*	490	55	1	546	**5.6**	**62**	1.35 (1.35)*

* For the intervals marked with an *, interference measurements are not robust due to low numbers of NPD tetrads.

Gene conversion events in these tetrads are given in S4 Table.

To our surprise, crossover recombination levels measured using genetic marker segregation analysis on spores from *S*. *cerevisiae* cells expressing *K*. *l*. *ZIP1* were nearly indistinguishable from wild-type levels (Table 2). Crossovers are typically reduced by 30–60% in mutant budding yeast strains that are missing a “class I” crossover pathway protein [20,53]. Accordingly, in our experiments cells missing the MutS component, Msh4, displayed 30%-73% (depending on the interval) of the wild-type level of crossovers (Fig 6B and Table 2). On the other hand, the map distances derived from four-spore viable tetrads of *K*.*l*. *ZIP1*-expressing strains were found to be within 90–105% of wild-type values. Two exceptions to this general finding existed in a pair of adjacent intervals on chromosome III: one of the two intervals exhibited 147% of the wild type map distance and the adjacent interval showed 69% of the wild-type map distance (Fig 6B and Table 2). The addition of these exceptional intervals thus gives a map distance that is 108% of our control (*S*.*c*. *ZIP1*-expressing) meiotic cells. Overall our data indicate that, for meioses resulting in four-spore viable tetrads (~8%, n = 7714, Table 1) the crossover recombination deficit of a *zip1* null [55,70] is completely rescued by expression of *K*. *l*. *ZIP1*.

To ask whether the rescue in crossover formation observed for *K*.*l*. *ZIP1*-expressing cells is specific to four-spore viable tetrads, we used random spore analysis to assess crossing over in the three-spore viable, two-spore viable, and one-spore viable tetrads that arose in the same crossover experiment described above. Like the four-spore viable tetrads, analysis of spores from three-, two- and one-spore viable *K*. *l*. *ZIP1*-expressing cells gave wild-type map distances (S1 Table). Furthermore, the frequency of chromosomes III displaying zero, single, double, triple and quadruple crossovers is similar between meiotic cells expressing *S*. *c*. *ZIP1* and cells expressing *K*. *l*. *ZIP1* (S2 Table). Thus, in meioses that are productive for spore formation, regardless of whether four-spore viable tetrads are produced, *K*. *l*. Zip1 rescues the crossover function of *S*. *c*. Zip1.

A question that our genetic data raises is why meioses in *K*. *l*. *ZIP1*-expressing cells with a wild-type crossover map (at least in the intervals measured) nevertheless result in reduced spore viability (Tables 1 and S3). One explanation for reduced spore viability despite wild-type crossover levels in *K*. *l*. *ZIP1*-expressing *S*. *cerevisiae* cells is that the *K*. *l*. Zip1 protein fails to provide a function at centromeres that normally supports proper MI segregation; such a function could provide centromere associations between the rare chromosome pairs that fail to sustain a crossover, or alternatively could ensure that crossover events do not occur within centromeric regions [67,71,72]. An additional or alternative possibility involves the distribution of crossovers on meiotic chromosomes, which normally exhibits measureable positive interference. The interfering distribution displayed by meiotic crossovers in wild type means that two crossover events are less likely to occur close to one another than expected from a random distribution of crossover events. In the case of weakened interference, some chromosomes (especially small chromosomes) will more frequently fail to establish stable chiasmata, relative to when strong interference is imposed [73]. Consistent with reduced interference, *K*. *l*. *ZIP1*-expressing strains exhibited a significantly elevated frequency of viable spores carrying a chromosome III with zero interhomolog crossovers among the intervals measured (*P* = 0.0004*)* (S2 Table).

We assessed interference among the crossovers detected in *S*. *c*. *ZIP1* and *K*. *l*. *ZIP1*-expressing strains in two distinct ways. First, we measured an “interference ratio” [74,75] by comparing the map distances of an interval when an adjacent interval had, or had not, experienced crossover recombination. To do this for intervals along chromosome III, we parsed tetrads that showed no evidence of recombination in a “reference” interval (Parental Ditype (PD) tetrads) from those tetrads containing a single or double crossover in that reference interval (Tetratype (TT) and Non-Parental Ditype (NPD) tetrads). Next we compared the distributions of tetrad types and map distances for an adjacent, “test” interval between the parsed groups—those associated with a non-recombinant reference interval versus those associated with a recombinant reference interval. The “interference ratio” is derived from the ratio of two map distances associated with the same test interval: the map distance calculated from tetrads in which the adjacent reference interval is recombinant (contains NPD or TTs) divided by the map distance calculated from tetrads that are PD for the reference interval. Since the two map distance values should approximate 1 in the case that a recombination event in an adjacent reference interval has no interfering effect on the frequency of crossing over in a test interval, the interference ratio gives an estimate of the strength of interference; a ratio of less than one can signify positive interference. The significance of differences between map lengths calculated for an interval in either the case of the recombinant or the non-recombinant reference interval was determined using Stahl Online Tools (http://molbio.uoregon.edu/~fstahl/), and a chi-square test was employed to determine if the distribution of tetrad types is considered significantly different in test intervals associated with the recombinant versus the non-recombinant reference interval (S5 Table). When both 1) the *P* value associated with comparing the distribution of tetrad types between test intervals and 2) the difference in the calculated map lengths were found to reflect statistical significance, we associated the interference ratio with positive interference (dark arrows in Fig 6C).

By this “interference ratio” method, positive interference was observed between two sets of genetic intervals on the right arm of chromosome III in wild-type strains (Fig 6C, top row, S5 Table). In contrast to previously obtained measurements of interference for *msh4Δ* mutant strains [25,30], this method did not indicate a strong diminishment in interference over these intervals in *msh4Δ* mutant strains. However, the method identified a uniform loss in positive interference for the two intervals examined in *S*. *cerevisiae* cells expressing *K*. *l*. *ZIP1* (Fig 6C, third line, S5 Table). The interference ratio values associated with *K*. *l*. *ZIP1*-expressing *msh4Δ* cells (Fig 6C, fourth line, S5 Table) appeared broadly similar to *K*. *l*. *ZIP1*-expressing, *MSH4* cells. Thus, according to this method for estimating the strength of interference, the wild-type levels of Msh4-independent crossovers promoted by *K*. *l*. Zip1 exhibit little interference, while (unexpectedly) the Msh4-independent crossovers observed in *S*. *c*. *ZIP1*-expressing meiotic cells exhibit significant levels of positive interference. The reason that interference among crossovers in *S*. *c*. *ZIP1 msh4Δ* strains was detected by the “interference ratio” method is unknown.

Interference can also be detected by a lower-than-expected incidence of NPDs, which normally arise from a double crossover within a single interval. The observed number of NPDs is compared to the number expected in the case of a random distribution of crossovers (i.e. no interference), using the equation of Papazian (1952) [76]. Using this latter method for analyzing interference we found that, compared with *MSH4 S*. *c*. *ZIP1*-expressing strains, crossover interference in *msh4Δ* mutants is nearly ablated in all intervals assessed, while crossover interference appears reduced (although not ablated) for every interval assessed in *S*. *cerevisiae* cells expressing *K*. *l*. *ZIP1* (Table 2 and Fig 6D).

In summary, both measurements of interference identified a defect in crossover patterning in *K*. *l*. *ZIP1*-expressing cells. The basis for why the interference defect (for both *msh4Δ* mutant, and *K*. *l*. *ZIP1*-expressing cells) appears stronger in one versus the other measurement remain unclear.

Our genetic analysis of interhomolog recombination in spores from *K*. *l*. *ZIP1*-expressing cells uncovered one additional deviation from wild-type: The frequency of gene conversion events in *K*. *l*. *ZIP1*-expressing *S*. *cerevisiae* meiotic cells that were productive in spore formation was elevated at eight out of nine loci (S4 Table). This result, in conjunction with the absence of SCs in *K*. *l*. *ZIP1*-expressing cells, is consistent with the idea that the SC structure prevents additional interhomolog recombination events in budding yeast, perhaps through a mechanism involving a downregulation of DSBs [77]. It is also possible that an altered gene conversion tract length for *K*. *l*. Zip1-mediated recombination events contributes to the elevated gene conversion frequency observed. We note that the 2–3 fold elevated gene conversion frequencies in *K*. *l*. *ZIP1*-expressing cells is not accompanied by an increase in the frequency interhomolog crossover events (over wild-type levels).

### *K*. *lactis* Zip1-promoted crossovers form largely independently of the MutSγ component, Msh4

The MutSγ heterodimer Msh4/Msh5 is required for the class I crossovers mediated by *S*. *c*. Zip1 [27,30,34,53,78]. Consistent with prior reports, we observed that the loss of *MSH4* in wild-type cells resulted in 30–70% reductions in crossover levels (Table 2). In contrast, our genetic analysis revealed that the bulk of the crossovers mediated by *K*. *l*. Zip1 in *S*. *cerevisiae* cells occur in a Msh4-independent manner. In *K*. *l*. *ZIP1 msh4Δ* strains, map distances are reduced, relative to *K*. *l*. *ZIP1 MSH4* strains, by less than seven percent in every interval measured with two exceptions: a 22% reduction in the *MAT-RAD18* interval in chromosome III and a 33% reduction in the *iLEU2-iTHR1* interval on chromosome XI (Fig 6 and Table 2). Overall, the crossover reductions observed when Msh4 is removed from *K*. *l*. *ZIP1*-expressing strains are dramatically less pronounced than the crossover reductions resulting from the removal of Msh4 in *S*. *c*. *ZIP1*–expressing strains. These data indicate that *K*. *l*. Zip1 rescues crossover formation in *S*. *cerevisiae* cells through a mechanism that does not rely heavily on the MutSγ component, Msh4.

Perhaps not surprisingly given its dispensability in crossover formation, the abundance of Msh4 on mid-meiotic prophase chromosomes in *K*. *l*. *ZIP1*-expressing cells is severely diminished relative to Msh4’s abundance on meiotic chromosomes in *S*. *c*. *ZIP1*-expressing cells (S5 Fig). Consistent with previous reports, we observed ~40–65 Msh4-HA foci co-localized with Zip3-MYC protein on mid-meiotic prophase chromosomes from *S*. *c*. *ZIP1*-expressing meiotic cells (at a stage when chromosomes normally exhibit full-length SC). In contrast, only 0–20 Msh4-HA foci were observed on similarly staged meiotic chromosomes from *K*. *l*. *ZIP1*-expressing cells; such low levels of Msh4 on meiotic chromosomes resembled the level detected in a *zip1* null mutant (S5 Fig).

### Msh4-independent *K*. *lactis* Zip1-mediated crossovers in *S*. *cerevisiae* cells rely on SIC proteins and Mlh3

In order to measure crossover recombination among all meiotic cells regardless of their capacity to successfully form spores, we turned to a physical assay for recombination on chromosome III. In this “circle-linear” assay, meiotic nuclei harboring one linear and one circular chromosome III are subjected to pulsed-field gel electrophoresis followed by a Southern blot to detect the position of chromosome III on the gel [79]. The circular chromosome III fails to enter the gel and thus is not detectable. However, the non-recombinant and recombinant forms of linear chromosome III fall into three size categories that are detectable on these gels: A single crossover between linear and circular chromosomes III runs at twice the molecular weight of the parental linear chromosome III, whereas a double crossover involving three chromatids runs at three times the molecular weight of the parental chromosome III. The proportion of trimer and dimer chromatids relative to the total (detectable) chromatids can be used to generate a relative measure of crossing over on chromosomes III in the population.

**Fig 7:**
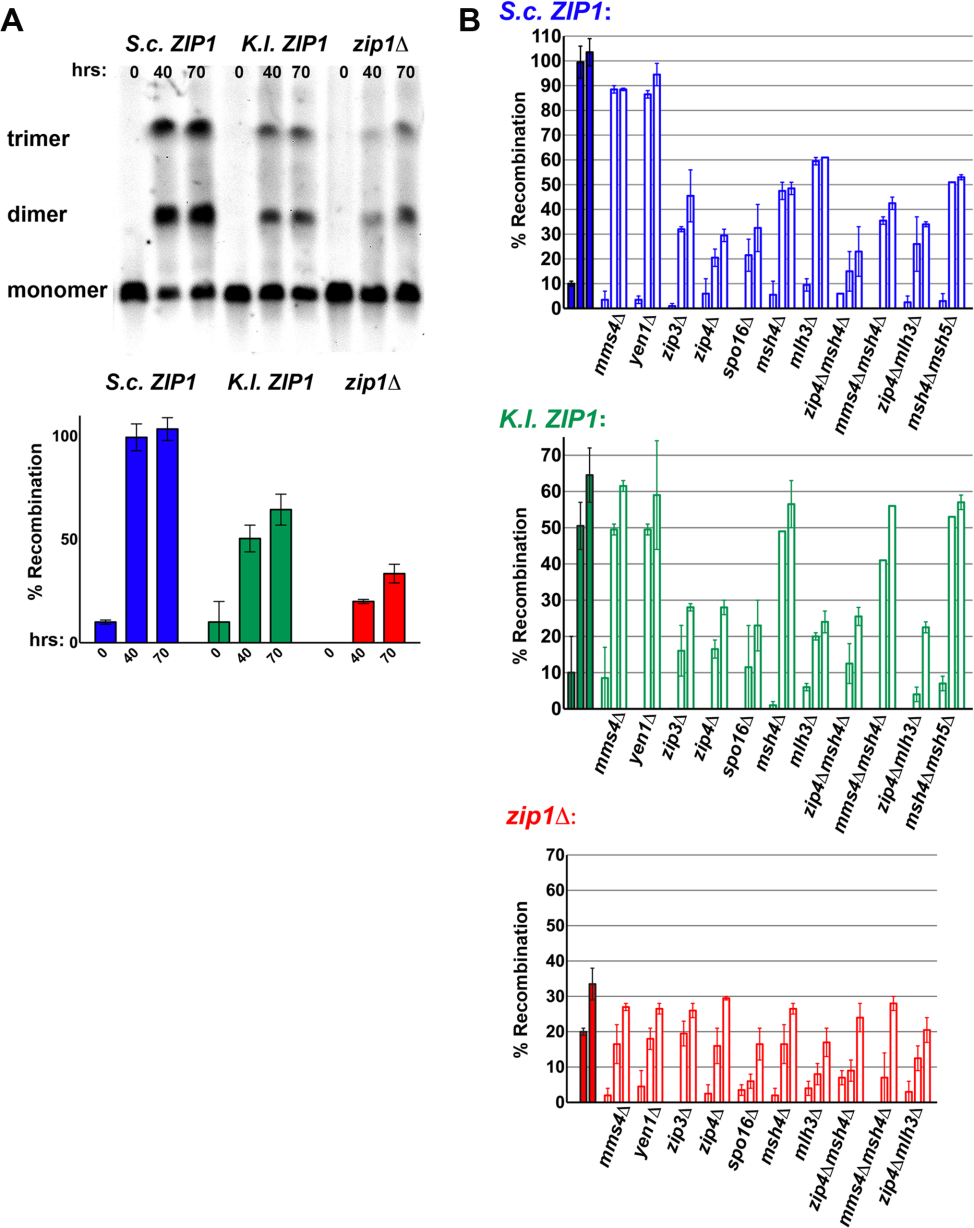
Crossovers mediated by *K*. *lactis* Zip1 are dependent on Zip3, Zip4, Spo16 and Mlh3 but independent of Msh4, Msh5 and non-MutSγ-MutLγ crossover pathway components. Sporulating cultures of *S*. *cerevisiae* strains carrying one linear and one circular chromosome III and carrying either *S*. *c*. *ZIP1* (K479), *K*. *l*. *ZIP1* (K457) or a *zip1* null (TY521, [18]) allele were embedded in agarose plugs, processed, run on a pulsed-field gel and analyzed by southern blot using a probe to chromosome III sequences (see Methods). Aliquots of sporulating cells were taken at 0, 40, and 70 hours after placement in sporulation medium. (A) shows an example blot that displays bands corresponding to different sized versions of linear chromosome III for the three strains indicated above. Circular chromosomes III present in these strains do not enter the gel. The lowest molecular weight band represents the size of endogenous (linear) III, while the middle and upper bands represent crossover products between linear and circular III; a single crossover event results in a linear chromatid III that runs at the size of the middle band (“dimer”) while a double crossover event involving 3 sister chromatids (of which 2 are circular) produces a “trimer” chromatid III which migrates at the position of the upper band. Plotted on the bar graph below is a value estimating % recombination observed on chromosome III (see Methods) for *S*. *c*. *ZIP1* (blue), *K*. *l*. *ZIP1* (green) or *zip1* null (red) strains at each meiotic time point (see Methods). Open bars displayed by the three graphs in (B) plot the relative % recombination measured for *S*. *c*. *ZIP1* (blue, top), *K*. *l*. *ZIP1* (green, middle) or *zip1* null (red, bottom) strains that are additionally missing the function of a class I or class II meiotic crossover pathway gene (listed on *x* axis). Each set of three adjacent bars represents samples harvested at 0, 40 and 70 hours (left to right) after placement in sporulation medium. Solid bars at far left of graphs in (B) are the % recombination values for *S*. *c*. *ZIP1*, *K*. *l*. *ZIP1* or *zip1* null strains from (A). Graphs plot an average and range for data from at least two independent experiments.

In wild-type strains, crossover recombination values estimated using this physical assay for crossovers on chromosome III were at nearly 100% by 40 and 70 hours of sporulation (Fig 7A). In *zip1* null mutants, on the other hand, approximately 20% and 30% recombination was measured at 40 and 70 hours after transfer to sporulation medium, respectively. In strains expressing *K*.*l*. *ZIP1*, approximately 50% and 65% crossover recombination was measured at 40 and 70 hours of sporulation, respectively (Fig 7A). Because *K*. *l*. *ZIP1*-expressing meiocytes that go on to form spores display wild-type levels of crossing over on chromosome III, the intermediate level of crossing over measured by this physical assay indicates that *K*. *l*. *ZIP1*-expressing meiocytes that fail to form spores are crossover-deficient.

We used this assay to explore whether *K*.*l*. Zip1-mediated crossovers are dependent on synapsis-associated proteins and MutLγ, or on the so-called “class II” crossover pathway components (Fig 7B). In control strains expressing *S*.*c*. *ZIP1*, removal of *MMS4* or *YEN1* (which encode proteins that have been genetically linked to the “class II” crossover pathway) resulted in a modest decrease (~10%) in the percentage of recombinant chromosomes III. In contrast, the individual removal of *ZIP3*, *ZIP4*, *SPO16*, *MSH4* or *MLH3* (each encoding a protein that has been linked to a discrete “class I” pathway for meiotic crossovers) resulted in a larger (50%-70%) reduction in crossover formation on chromosome III. *zip1* null strains missing *MMS4*, *YEN1*, or any of the “class I” crossover genes tested displayed similarly low levels of crossover recombination on chromosome III.

Analysis of *K*. *l*. *ZIP1*-expressing strains missing these crossover-associated genes revealed strong evidence that *K*. *l*. Zip1 functionally interfaces with a canonical Zip1/SC–associated crossover pathway in *S*. *cerevisiae* cells. Crossover recombination in *K*.*l*. *ZIP1*-expressing strains is strongly reduced (to nearly *zip1* null levels) in the absence of *ZIP3*, *ZIP4*, *SPO16*, or the MutLγ protein-encoding gene, *MLH3* (Fig 7B). On the other hand, crossover recombination on chromosome III was reduced only modestly (by ~10%) in *K*.*l*. *ZIP1*-expressing strains missing either *MMS4* or *YEN1* (Fig 7B), indicating that these DNA repair-associated factors are dispensable for the bulk of meiotic interhomolog crossovers in both wild-type and *K*. *l*. *ZIP1*-expressing strains.

Consistent with our genetic analysis, recombination on chromosome III was reduced only modestly (by ~10%) in *K*. *l*. *ZIP1*-expressing strains missing *MSH4*, and a similar result was obtained for *K*. *l*. *ZIP1*-expressing strains missing both *MSH4* and *MSH5* (Fig 7B). We did not find evidence that class II crossover pathway components rescue Msh4 function when it is absent from *K*. *l*. *ZIP1*-expressing cells, as the small reduction in crossovers on chromosome III measured in *K*.*l*. *ZIP1*-expressing strains missing both *MSH4* and *MMS4* was similar to that observed in either the *msh4Δ* or *mms4Δ* single mutant (Fig 7B).

Taken together, our data clearly indicate that *K*.*l*. Zip1, like *S*.*c*. Zip1, functionally interfaces with other synapsis-associated proteins in order to facilitate the maturation of MutLγ-associated crossovers in budding yeast, but that *K*. *l*. Zip1-mediated crossovers can largely bypass a requirement for MutSγ.

### *S*. *c*. Zip1 and *K*. *l*. Zip1 promote Msh4-independent joint molecule formation in *S*. *cerevisiae* meiotic cells

**Fig 8:**
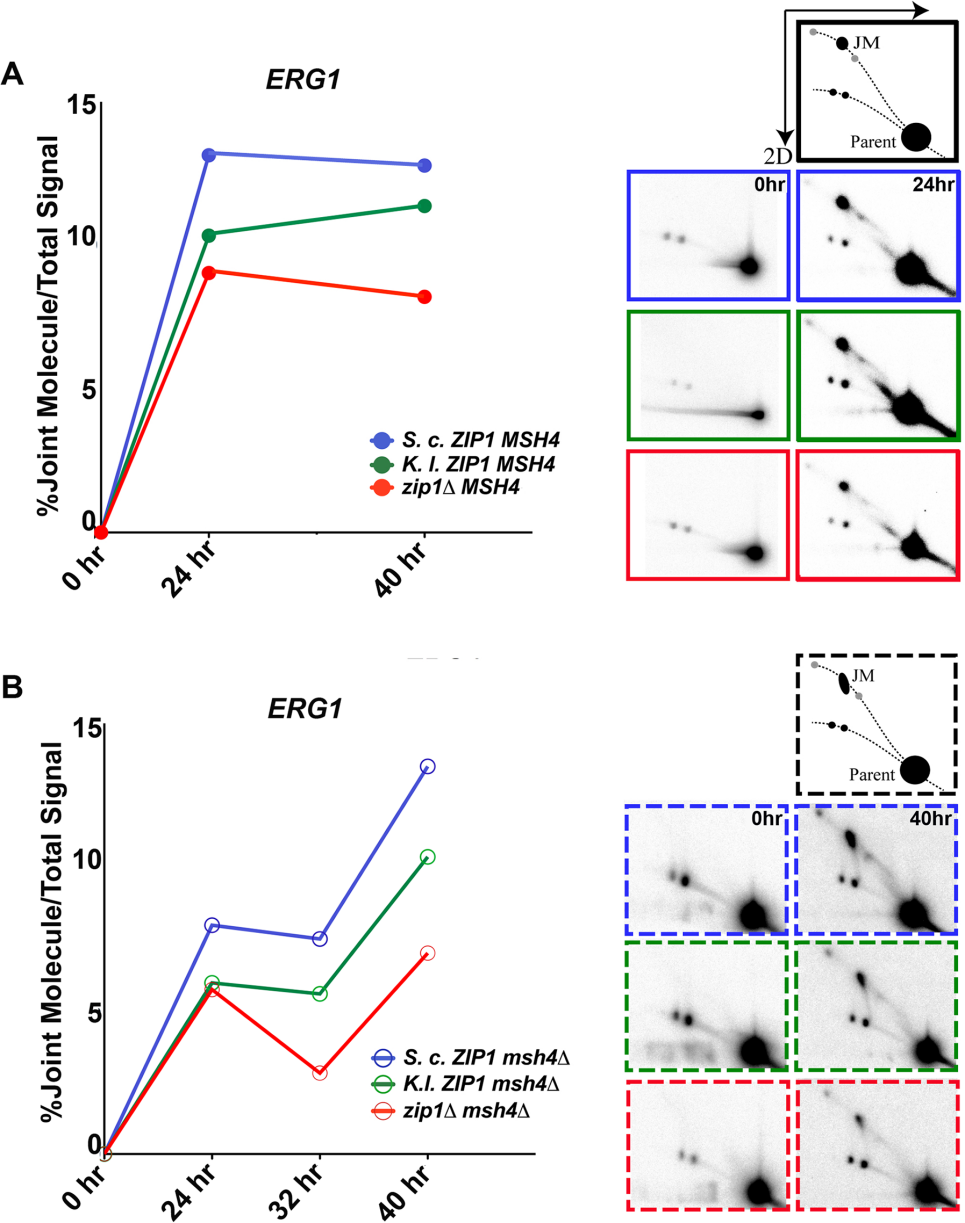
*S*. *c*. Zip1 and *K*. *l*. Zip1 promote Msh4-independent JM formation in *S*. *cerevisiae* meiotic cells. Sporulating cultures of *S*. *cerevisiae* strains carrying either *S*. *c*. *ZIP1* (K663), *K*. *l*. *ZIP1* (K666) or a *zip1* null (K669) allele in the *MSH4* background or *S*. *c*. *ZIP1* (K672), *K*. *l*. *ZIP1* (K675) or a *zip1* null (K678) allele in the *msh4* background were subject to psoralen crosslinking to preserve recombination intermediates (JMs; see Methods). Aliquots of sporulating cells were taken at 0, 24, 32, and 40 hours after placement in sporulation medium and crosslinked DNA was separated by 2D gel electrophoresis. In this assay, the linear DNA travels as an arc while branched recombination intermediates (including JMs) are slower migrating and are retarded from the arc of linear fragmented, crosslinked DNA. These molecules can be detected by Southern hybridization as shown in the schematic on the right and images below. The line graphs (A and B) are from a representative time course experiment and plot the percentage of JM/total DNA exhibited by each strain at the *ERG1* locus as a function of time. For any given time point, all three strains were analyzed on the same blot; time course experiments were analyzed at least twice with similar trends observed in each experiment. We note that although we do not know the true molecular nature of these JMs, the position of the strong signal–black intermediate labeled “JM” in the schematic—is consistent with that of the dHJ, while the faster migrating signal on the arc of branched molecules (lower grey spot in the schematic) may reflect single-end invasions (SEIs), and the slower migrating signal (higher grey spot in the schematic) could be from multi-chromatid JMs [23,41].

We examined the capacity for *K*. *l*. Zip1 to facilitate MutSγ-independent recombination in greater detail by asking whether *K*. *l*. Zip1 can rescue the JM deficit reported for cells missing MutSγ complex function [27]. We analyzed six strains, each carrying *S*.*c*. *ZIP1*, *K*.*l*. *ZIP1* or a *zip1* null allele, in either a *MSH4* or a *msh4* null background. As our strains (BR1919-8B-derived [69]) progress through meiosis in an asynchronous manner, we reasoned that we would be more likely to detect JMs if we prevent their resolution. Thus, each of our strains is also missing *NDT80* activity, which is normally required to promote the molecular pathways that resolve JMs into crossovers in *S*. *cerevisiae* [3,19], and is indeed required for crossover formation in *K*. *l*. *ZIP1*-expressing cells (S6 Fig). Cells were harvested at 0, 24, 32 and 40 hours after being introduced into sporulation medium, then subjected to psoralen crosslinking to preserve JM structures. Crosslinked DNA was extracted, digested with HindIII, and DNA fragments were separated by two-dimensional (2D) electrophoresis. The branched nature of crosslinked JMs causes them to migrate to a position on the 2D gel which is displaced from the arc of the bulk of crosslinked genomic DNA [5,35] (see cartoon in Fig 8). The positions of all DNA fragments that correspond to the *ERG1* and *YCR047c* loci, which are associated with DSB hotspots [35,77,80,81], were analyzed by Southern blot hybridization.

Signals representing JM structures were undetectable at the *t* = 0 time points in any of our strains. However, JMs were detectable at both *ERG1* and *YCR047c* sites in all strains at 24 hours after introduction into sporulation medium (Figs 8 and S7). Quantification of the percentage of DNA that was present in the JM spot in either *MSH4* or *msh4* strains is shown for the *ERG1* locus in Fig 8. In *MSH4* strains, we observed that both *K*. *l*. *ZIP1*-expressing samples and *zip1* null samples exhibited a diminished JM signal relative to *S*. *c*. *ZIP1* samples at the 24 hour time point. However, at the 40 hour time point the JM signal in *K*. *l*. *ZIP1* samples appeared closer to that of *S*. *c*. *ZIP1*, and elevated above the JM level exhibited by the *zip1* null. These data are consistent with the crossover data we obtained with the circle-linear chromosome III assay (Fig 7) and suggest that *K*. *l*. Zip1 has an (albeit diminished) capacity to facilitate stable JM formation, and that *K*. *l*. Zip1-dependent JMs accumulate over time in an *ndt80Δ* mutant background.

Support for the idea that MutSγ is critical for the bulk of JM formation in *S*. *cerevisiae* meiosis comes from the observation of strongly diminished JMs at the *HIS4-LEU2* artificial hotspot in *msh5* mutants (using the SK1 strain background) [27]. As Mlh3-dependent crossovers form in *K*. *l*. *ZIP1*-expressing cells despite the absence of Msh4, we wondered whether *K*. *l*. Zip1 rescues the deficit in JM formation presumed to occur in the absence of Msh4. We asked this question by analyzing JM formation in *S*. *c*. *ZIP1*, *K*.*l*. *ZIP1* and *zip1* null strains that were also missing *MSH4*.

In light of the strong reduction in JMs at the *HIS4-LEU2* artificial hotspot in *msh5* mutants, we were surprised to observe a robust JM signal at both *ERG1* and *YCR047c* in our *S*. *c*. *ZIP1 msh4Δ ndt80Δ* strains (Fig 8B, blue line, and S7 Fig). Furthermore, the JM signal at the *ERG1* site appeared to increase between 24 and 40 hours of sporulation in the *ndt80*-arrested, *msh4* mutants. We presume that the extensive period of late prophase arrest performed for our analysis facilitated the slow but steady accumulation of JMs even in the absence of Msh4. Consistent with this possibility, a prior report demonstrated that *msh5Δ ndt80Δ* mutants of the SK1 background exhibit JM accumulation over time, ultimately achieving ~1/3 of the peak wild-type JM level by a late (8 hour) time point [41].

Our analysis thus reveals the existence of Msh4-independent JMs that accumulate in an *ndt80Δ*, meiotic prophase-arrested cell population at *ERG1* and *YCR047c* sites in the BR1919 strain. Interestingly, we observed that a substantial fraction of the Msh4-independent JM signal at *ERG1* and *YCR047c* sites in *S*. *cerevisiae* is dependent on Zip1. In *zip1 msh4* double mutants (Fig 8B, red line), JMs do not accumulate to the same high levels as seen in the *ZIP1 msh4* strain. Thus, our data indicate that *S*. *c*. Zip1 can promote Msh4-independent JM formation.

Since *msh4* mutants are missing the same set of crossovers as *zip1* mutants [30], the bulk of the Msh4-independent JMs promoted by *S*. *c*. Zip1 (the set of JMs present in *S*. *c*. *ZIP1*-expressing cells but not present in *zip1* null cells) are not likely resolved to form interhomolog crossovers. This observation raises the possibility that the crossover defects of *S*. *c*. *ZIP1 msh4* mutants may not be solely the result of a deficit in JM formation *per se*, but rather could be in part the result of a function for Msh4 in channeling SIC protein-associated recombination intermediates into an interhomolog JM pathway that is resolved by Mlh1/Mlh3.

Finally, our analysis revealed that, like *S*. *c*. Zip1, *K*. *l*. Zip1 promotes Msh4-independent JM formation in *S*. *cerevisiae* cells, albeit with a reduced capacity relative to *S*. *c*. Zip1 (Fig 8B, green line, and S7 Fig). While the Msh4-independent JMs in *S*. *c*. *ZIP1*-expressing cells presumably do not resolve to give interhomolog crossovers, in light of our genetic and physical crossover data (Figs 6 and 7) we propose that a substantial fraction of the Msh4-independent JMs in *K*. *l*. *ZIP1*-expressing cells are successfully resolved into interhomolog crossovers via an Mlh3-dependent mechanism.

Although experiments aimed at understanding the molecular nature of Msh4-independent JMs are outside the scope of the current work and will be the subject of a future study, we note that the shape of the JM signal in *msh4* mutants appears elongated relative to that of the JMs observed in *MSH4* strains (illustrations in Fig 8A and 8B). The elongated shape of the observed signal for Msh4-independent JMs in our strains suggest the presence of JM species with a similar molecular mass but different branched pattern, possibly the result of an altered dHJ structure or perhaps from junction migration in the *msh4* mutants. An alteration in the structure of dHJs in the absence of Msh4 is consistent with the finding that hMSH4-hMSH5 recognizes Holliday Junctions and can potentially form a clamp which “embraces” partner DNA molecules of homologous chromosomes [36,82].

## Discussion

### A version of Zip1 that promotes Mlh3-dependent crossovers but not SC assembly

Here we report on the capacity of the *Kluyveromyces lactis* Zip1 protein to carry out *S*. *cerevisiae* Zip1 functions in the *S*. *cerevisiae* meiotic cell context. *Kluyveromyces lactis* and *S*. *cerevisiae* last shared a common ancestor well over 100 million years ago, prior to the fungal lineage’s whole genome duplication event [83]. The *K*. *lactis* genome encodes apparent homologs of most if not all synapsis-related proteins that have been thus far characterized in *S*. *cerevisiae* (including SUMO, Hop1, Red1, Ecm11, Gmc2, Zip2, Zip3, Zip4, Spo16, and Pch2), as well as the Msh4, Msh5, Mlh1 and Mlh3 proteins (http://www.genome.jp/kegg-bin/show_organism?org=kla). Whether *K*. *lactis* meiotic cells assemble an SC is unknown.

*K*. *l*. Zip1 exhibits ~ 40% overall homology with *S*. *c*. Zip1 at the primary amino acid level, and *K*. *l*. Zip1 and *S*. *c*. Zip1 share predicted structural characteristics. In particular, both *K*. *l*. Zip1 and *S*. *c*. Zip1 have a ~550 residue, centrally located group of amino acids that have a high probability of forming coiled-coil. The N- and C- terminal, non-coiled-coil regions of *S*. *c*. Zip1 are ~30–40% larger than the corresponding regions of *K*. *l*. Zip1. Several ~5–20 residue blocks of conserved sequence identity exist between the two ancestrally related proteins (Fig 1).

*K*. *l*. Zip1 fails to assemble mature SC structures in *S*. *cerevisiae* cells, as indicated by the absence of full-length linear Zip1, Ecm11 or SUMO assemblies on meiotic prophase chromosomes at any time point during meiotic prophase, and by the asynapsis phenotype of Red1-labeled chromosome axes (Figs 2, 3 and 5). Apart from a distinct polycomplex aggregate, little *K*. *l*. Zip1 was detectable on *S*. *cerevisiae* meiotic prophase chromosomes in our experiments, including those that assessed the distribution of an epitope-tagged version of *K*. *l*. Zip1 (Figs 2–5 and S1). Moreover, levels of the SC- and/or crossover-associated Zip3, Zip4, and Msh4 proteins on *S*. *cerevisiae* prophase chromosomes appeared similar to the levels of these proteins in *zip1* null cells (Figs 4 and S2 and S5).

However, evidence that *K*. *l*. Zip1 can interface, at least to some extent, with *S*. *cerevisiae* SC-associated proteins stems from the observation that *K*. *l*. Zip1 polycomplex structures are decorated by *S*. *c*. Ecm11, SUMO and Zip3 proteins (Figs 2–5 and S1). Furthermore, *K*. *l*. Zip1 promotes SUMOylation of the *S*. *cerevisiae* Ecm11 protein (Fig 3), an activity that normally also largely relies on the function of synapsis proteins Zip2 and Zip4 [12]. Finally, the interhomolog crossover events that are promoted by *K*. *l*. Zip1 in *S*. *cerevisiae* cells are dependent on other so-called SIC proteins, namely Zip3, Zip4 and Spo16 (Fig 7). These observations suggest that at least some molecular features of *S*. *c*. Zip1 responsible for interfacing with SC-associated proteins are preserved in the *K*. *l*. Zip1 protein. Such molecular features could be represented by the short segments of identical sequence shared by the two Zip1 proteins, and/or may be based in a shared secondary structure.

The sparse distribution of detectable *K*. *l*. Zip1 and the reduced number of Zip3 and Zip4 proteins observed on meiotic prophase chromosomes in *S*. *cerevisiae* expressing *K*. *l*. *ZIP1* suggests that SC precursor structures and/or their associations with chromosomes are unstable in this context. Because *zip1* loss-of-function mutants and other *S*. *cerevisiae* mutant meiotic cells with such a dramatic asynapsis phenotype typically also exhibit a deficit in crossovers [16,18,27,70], we were surprised to measure wild-type levels of crossover recombination in spores derived from *K*. *l*. *ZIP1*-expressing *S*. *cerevisiae* meiotic cells (Fig 6). A combination of genetic and physical assays to measure crossing over revealed that *K*. *l*. Zip1-mediated crossovers are dependent on the SC-associated proteins Zip3, Zip4 and Spo16, and are dependent on the MutLγ protein Mlh3, but are relatively unaffected by the loss of Mms4 and Yen1, which is as expected for Zip1-mediated (SC-associated) crossover events (Fig 7). Furthermore, the resolution of *K*. *l*. Zip1-mediated repair intermediates into crossovers is, like most if not all meiotic crossovers in *S*. *cerevisiae*, dependent on the Ndt80 transcription factor (S6 Fig). Our data strengthen the notion that at least one pro-crossover function of Zip1 is separate from its role in assembling SC, a possibility previously raised by an analysis of the *red1* mutant in the presence and absence of Zip1 and to a certain extent by analysis of the recombination phenotype of *zip1* mutants [27,55]. *K*. *l*. Zip1’s behavior in *S*. *cerevisiae* cells demonstrates that these independent activities of Zip1 can be uncoupled at the protein level.

If *K*. *l*. *ZIP1*-expressing cells rely on other SC-associated proteins to promote crossing over, why is the level of Zip3 and Zip4 on meiotic chromosomes in *K*. *l*. *ZIP1*-expressing cells at the low level seen in the *zip1* null? One possibility is that nascent SC-initiation structures are dynamic in the absence of elaborated SC, and thus only a subset of the so-called SIC complexes are detectable on meiotic chromosomes at a given time in the *zip1* null or the *K*. *l*. *ZIP1* context. The discrepancy between the low observed level of *K*. *l*. Zip1 protein on *S*. *cerevisiae* meiotic chromosomes and the high level of crossovers observed in at least a subset of *S*. *cerevisiae* meiotic cells expressing *K*. *l*. *ZIP1* raises the important point that the abundance and spatial distribution of a protein that is minimally sufficient to provide crossover function may not necessarily be detectable by immunostaining.

### Mlh3-dependent crossover levels, independent of the SC, are tightly correlated with overcoming a checkpoint-imposed block to spore formation

A discrepancy exists between our genetic and physical analyses of crossing over in *S*. *cerevisiae* cells expressing *K*. *l*. *ZIP1*. When measured genetically in spores, *K*.*l*. *ZIP1*-expressing strains exhibit wild-type map distances within intervals across chromosome III, and within intervals on two additional chromosomes (VIII and XI; Fig 6 and Table 2). On the other hand, by our physical assay we observed an intermediate crossover level across chromosome III in strains expressing *K*.*l*. *ZIP1*, relative to the levels exhibited by *S*.*c*. *ZIP1* and *zip1* null strains (Fig 7). Similarly, *K*.*l*. *ZIP1 msh4Δ* double mutants exhibit significantly higher crossover levels relative to *S*.*c*. *ZIP1 msh4Δ* strains when measured genetically, but crossover levels across chromosome III are at comparable levels in *K*.*l*. *ZIP1 msh4Δ* and *S*.*c*. *ZIP1 msh4Δ* strains by our physical assessment.

The discrepancy between crossover levels measured genetically versus a physical assay is likely due to a Pch2-mediated, prophase surveillance system that blocks spore formation in the majority of *K*. *l*. *ZIP1*-expressing meiotic cells. The triggers that activate a Pch2-mediated checkpoint have been associated with defects in both synapsis and in DSB repair, and can be modulated by environmental factors in budding yeast [27,57,84–87]. Our data suggest that this meiotic prophase checkpoint activity is more robust in *K*.*l*. *ZIP1 msh4Δ* than in *S*. *c*. *ZIP1 msh4Δ* cells as the sporulation efficiency of *K*.*l*. *ZIP1 msh4* strains is lower than the sporulation efficiency of *S*.*c*. *ZIP1 msh4* strains (16.6% for *K*.*l*. *ZIP1 msh4* versus 30.0% for *S*.*c*. *ZIP1 msh4*; n > 1000).

Overcoming a block to meiotic progression could occur either by removing or by bypassing the insult that triggered the checkpoint. The phenotype observed in *K*.*l*. *ZIP1*-expressing, *S*. *cerevisiae* strains, where only those meiocytes with a nearly wild-type interhomolog crossover level progress to form spores, appears to underscore the strong influence that SC protein-associated, MutLγ-mediated recombination can have on overcoming the Pch2-associated checkpoint (regardless of how the checkpoint is triggered in these cells). Our data indicate that a capacity to overcome the prophase checkpoint in *K*. *l*. *ZIP1*-expressing cells tightly correlates with crossover recombination outcomes: Wild-type crossover levels are measured for *K*. *l*. *ZIP1*-expressing meiotic nuclei that succeed in forming spores, but crossover recombination is lower (intermediate between the levels exhibited by *zip1* null and wild-type) when examined by a physical assay, an analysis that includes meiotic cells that are blocked from progressing to form spores. Since mature SC is absent in *S*. *cerevisiae* cells expressing *K*. *l*. *ZIP1*, these data suggest that crossover levels alone, independent of the SC, may be sufficient to overcome the Pch2-mediated prophase checkpoint block to spore formation.

On the other hand, the *S*. *c*. *ZIP1 msh4* mutant phenotype is difficult to explain with the simple model that crossover levels overcome the prophase checkpoint to allow spore formation, since *msh4* meiotic cells with strongly diminished crossovers can nevertheless successfully form spores. Perhaps an absence of MutLγ-associated recombination intermediates in *S*. *c*. *ZIP1 msh4* cells results in a less stringent checkpoint activity than that observed in *K*. *l*. *ZIP1*-expressing cells. Alternatively, perhaps the SC structure is capable of modulating the prophase checkpoint [21,88]. Previous reports indicate that SC formation is delayed but ultimately occurs to some extent in *msh4* mutants [17,25,30]; thus the increased capacity of cells deficient in MutSγ-associated crossovers to complete spore formation could be a consequence of signals from the SC structure itself that overcome the checkpoint.

### The relationship between Zip1, the SC, and MutLγ crossover formation

While prior studies indicated that Zip1 might play a role in recombination separate from its role in SC assembly, the question remained whether the SC structure has a mechanistic role in the formation of a set of crossovers that are normally associated with synapsis. The presence of *K*. *l*. Zip1 as the sole source of Zip1 in *S*. *cerevisiae* cells fails to support SC assembly but promotes the formation of a set of crossovers that are Mlh3-dependent. Thus, this unique separation-of-function version of Zip1 demonstrates that an elaborated SC structure is not required *per se* for the formation of Mlh3-dependent crossovers in *S*. *cerevisiae*.

By what mechanism is Zip1 (including *K*. *l*. Zip1) involved in MutLγ-associated crossover recombination? The notion that Zip1 acts early in the pathway leading to stable crossover recombination intermediates could account for Zip1’s entire role in promoting MutLγ-dependent events, via a function in shaping or stabilizing proper JM structures that are recognizable and/or accessible to MutLγ and its companion resolvase-promoting factors. On the other hand, current data does not rule out the idea that Zip1 protein acts at later stages in the JM maturation process to facilitate the targeting of MutLγ proteins to MutSγ-associated crossover intermediates. Our analysis of JM formation in *S*. *c*. *ZIP1*, *K*. *l*. *ZIP1* and *zip1* null cells (Figs 8 and S7) supports either model: *K*.*l*. Zip1 appears to promote some stable JM formation above the level seen in the *zip1* null, consistent with the idea that *K*. *l*. Zip1 might act early to promote the formation of a stable JM structure. Our observation of *S*. *c*. or *K*. *l*. Zip1-dependent JMs in *msh4* mutants also supports a role for Zip1 in establishing a stable JM structure. On the other hand, the fact that (in the *S*. *c*. *ZIP1* context) *S*. *c*. Zip1-dependent JMs form in the absence of Msh4 but do not resolve properly highlights the possibility that an *S*. *c*. Zip1-mediated constraint linking MutLγ resolvase activity to MutSγ-associated recombination intermediates may act downstream of or in parallel to stable JM formation, (see below).

Importantly, while spores from *K*. *l*. *ZIP1*-expressing cells exhibit nearly wild-type crossover levels over multiple genetic intervals, this result is influenced heavily by a stringent meiotic checkpoint; it is certainly not the case that all meiotic cells enjoy wild-type crossover levels in *K*. *l*. *ZIP1*-expressing *S*. *cerevisiae* strains (as indicated by our physical assays of crossover recombination on chromosome III). We imagine that the capacity of *K*. *l*. Zip1 to promote crossover recombination in *S*. *cerevisiae* cells is diminished relative to *S*. *c*. Zip1 because of suboptimal protein function and/or diminished protein levels.

Our physical and genetic crossover analyses indicate that a small subset of *S*. *cerevisiae* meiotic cells exhibit nearly wild-type levels of crossing over on chromosome III. If *K*. *l*. Zip1 only partially rescues *S*. *c*. Zip1’s crossover function, why do some cells experience a wild-type level of crossing over in the context of *K*. *l*. Zip1? Thacker et al. (2014) reported that *zip1* mutants fail to properly down-regulate DSBs at later meiotic prophase stages [77]. With the idea in mind that the presence of SC could participate in down-regulating recombination-based interhomolog interactions, we propose that ongoing DSB-initiated interhomolog interactions allowed because of the absence of SC in *K*. *l*. *ZIP1*-expressing cells may be critical for the gradual establishment of a class of *K*. *l*. *ZIP1*-expressing cells that achieve wild-type interhomolog crossover levels.

### *K*. *l*. Zip1 bypasses the requirement for Msh4/Msh5 in Mlh3-dependent crossover formation

Data presented in this study indicate that *K*. *l*. Zip1 retains a robust pro-crossover activity that functionally interfaces with several canonical Zip1 crossover pathway factors, including the so-called SIC proteins Zip3, Zip4, Spo16, and the MutLγ protein Mlh3 in *S*. *cerevisiae* cells. However, *K*. *l*. Zip1 crossovers in the context of the *S*. *cerevisiae* cell are different from *S*. *c*. Zip1-associated crossovers in two ways: First, *K*. *l*. Zip1 crossovers are unassociated with SC formation. Second, in the context of *K*. *l*. Zip1, MutLγ-mediated crossovers bypass a requirement for the MutSγ complex.

In *K*. *l*. *ZIP1*-expressing cells, Mlh3 promotes the resolution of recombination intermediates into crossovers even in the absence of MutSγ complex proteins Msh4/Msh5. While evidence from both *Tetrahymena* and *C*. *elegans* indicates that eukaryotic versions of bacterial MutS may not always be functionally linked to MutL proteins [48–50], *K*. *l*. *ZIP1*-expressing *S*. *cerevisiae* meiotic cells reflect the first example, to the authors’ knowledge, of MutLγ-dependent crossover formation that does not rely on MutSγ. The result indicates that MutLγ is not intrinsically constrained to act on MutSγ-associated DNA structures in *S*. *cerevisiae* nuclei, but that a constraint is normally active in the context of *S*. *cerevisiae ZIP1* that normally couples MutLγ to MutSγ-associated recombination intermediates. Our study furthermore demonstrates that *K*. *l*. Zip1 can bypass this constraint. Understanding how *K*. *l*. Zip1 bypasses the requirement for Msh4/Msh5 in generating MutLγ-associated crossovers will provide a useful framework for understanding the molecular mechanism normally used by budding yeast to couple MutLγ-associated resolvase activity to MutSγ-associated intermediates.

It is noteworthy that Zip1 appears to be central to the mechanism that normally links MutLγ-associated resolvase activity to MutSγ-associated intermediates in budding yeast. As raised in the Introduction, one explanation for the role of SC structural proteins such as Zip1 in meiotic interhomolog recombination is that SC proteins or the SC itself act as a recruitment platform upon which specialized recombination enzymes can dock. While the data presented here do not rule out this model, the fact that an alternate version of Zip1 can bypass the requirement for MutSγ in MutLγ-mediated crossover formation raises the possibility that Zip1 may play a more specialized role in the processing of joint molecule intermediates.

Does *K*. *l*. Zip1 promote Msh4-independent crossovers by replacing Msh4/Msh5’s function in JM formation? The Msh4/Msh5 heterodimer is thought to form a sliding clamp on DNA and thus could recognize and stabilize both SEI and dHJ structures [27,36] in order to protect them from disassembly by helicases [40] and/or to facilitate their resolution by MutLγ-Exo1 [23,33,38,40–42]. Interestingly, the pro-crossover function(s) of Msh4/Msh5 in stabilizing meiotic crossover recombination intermediates are replaced by novel minichromosome maintenance protein complex in *Drosophila* [52]. Furthermore, proteins that promote SC formation have been implicated in antagonizing the anti-crossover activity of the Sgs1 helicase, consistent with an the idea that these proteins may share functionality with Msh4-5 in protecting JM structures from dissolution by helicases [40]. Prior observations of the DNA intermediates that accumulate at the *HIS4-LEU2* artificial DSB hotspot in *msh5* mutant strains (of the SK1 background) are consistent with the idea that Msh4/Msh5 activity is required for the accumulation of the bulk of stable dHJ recombination intermediates [27,41], although Msh5-independent JMs were found to accumulate over time in an *ndt80Δ* mutant background [41]. If *K*. *l*. Zip1 can bypass the need for Msh4 through rescuing a function of Msh4 in promoting stable JMs, we expected to observe a larger abundance of JMs in *K*. *l*. *ZIP1 msh4* mutants, relative to *S*. *c*. *ZIP1 msh4*. Surprisingly, our analysis of JM formation at two natural hotspots (*ERG1* and *YCR047c*) in *MSH4* and *msh4* mutant strains of the BR1919 background indicate that *S*. *c*. Zip1 and *K*. *l*. Zip1 (to a lesser extent) both promote the formation of a population of Msh4-independent JMs, although the elongated shape of the observed signal suggests the possibility that JMs that form in the absence of Msh4 in our strains have altered structure.

While these data reveal the interesting result that Msh4 and Zip1 may indeed share overlapping roles upstream of JM formation, JM formation activity *per se* is not likely to be the reason that Msh4 is dispensable for MutLγ-dependent crossovers in *K*. *l*. *ZIP1*-expressing strains, since both *S*. *c*. Zip1 and *K*. *l*. Zip1 promote Msh4-independent JM formation. We presume that only in the context of *K*. *l*. *ZIP1* can such Msh4-independent JMs resolve via MutLγ.

Perhaps the critical crossover function of MutSγ in *S*. *cerevisiae*, instead of JM formation *per se*, is in ensuring that MutLγ-associated resolvase activity is successfully targeted to SC-associated JMs. When *K*. *l*. Zip1 is present, MutLγ is targeted to SIC protein-dependent crossover intermediates independently of MutSγ. Evidence that mammalian MutSγ and MutLγ components can directly interact [89] raises the possibility that Msh4/Msh5 might directly recruit Mlh1/Mlh3 complexes to JM structures. If the major mechanism for targeting MutLγ to MutSγ-associated JMs involves a direct protein-protein interaction between MutSγ and MutLγ components, one might propose that *K*. *l*. Zip1 bypasses the normal requirement for Msh4/Msh5 via a capacity to directly interact with Mlh1 or Mlh3 in *S*. *cerevisiae* cells. On the other hand, *S*. *cerevisiae* MutLγ complex can recognize and bind preferentially to JM structures *in vitro* [38]. Thus, perhaps the critical role of Msh4/Msh5 in coupling SC-associated recombination intermediates with MutLγ-associated resolvase activity is not through its potential capacity to interact directly with Mlh1/Mlh3, but through a capacity to promote the formation of a JM structure that is recognizable and/or accessible to the *S*. *cerevisiae* MutLγ complex. In this case, a simple explanation for the bypass of MutSγ provided by *K*. *l*. Zip1 is that *K*. *l*. Zip1 activity is functionally redundant with Msh4/Msh5 in *S*. *cerevisiae* meiotic nuclei (this idea is reminiscent of the functional redundancy with MutSγ proposed for the minichromosome maintenance protein complex in *Drosophila* [52]) and can facilitate the processing of JM intermediates in a manner that allows their resolution by a MutLγ-mediated mechanism.

**Fig 9:**
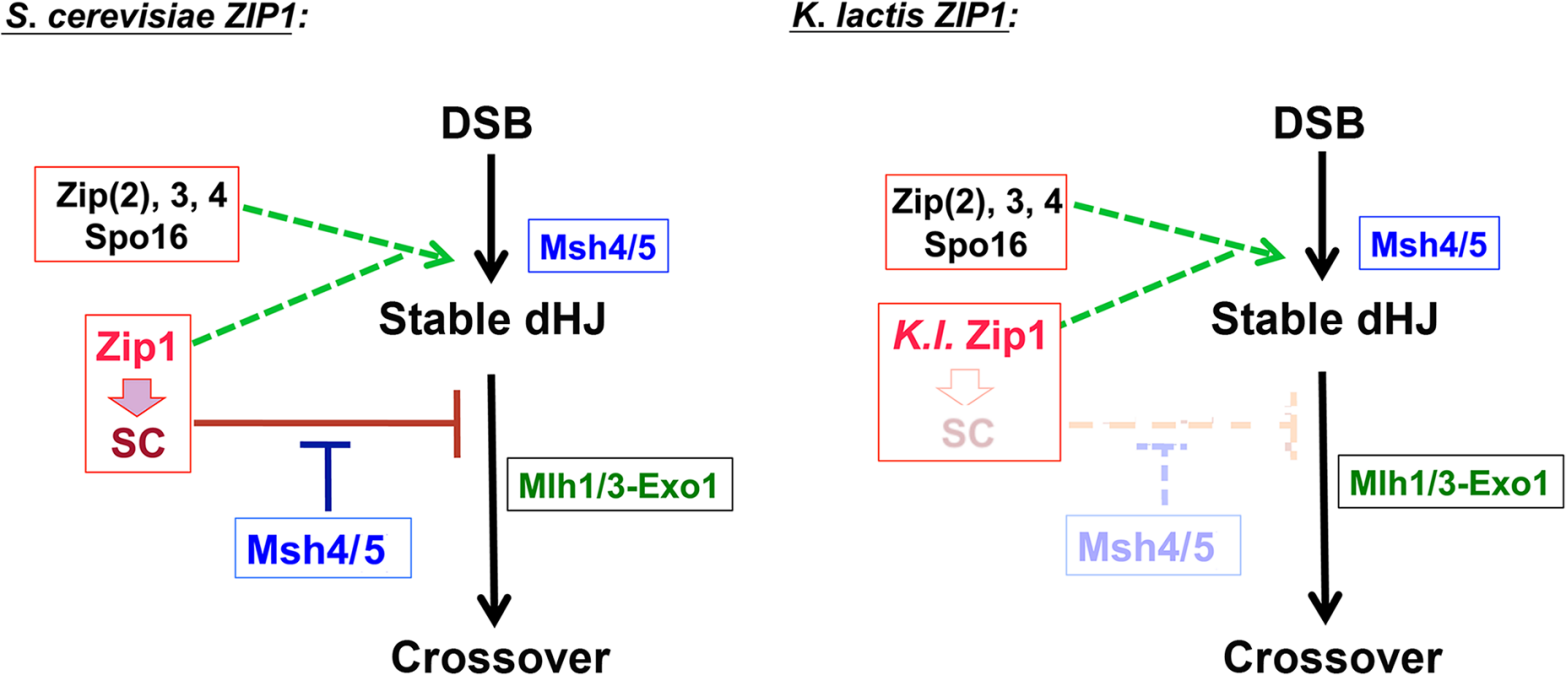
One model to explain the constrained relationship between MutSγ, SIC/Zip1-associated JMs, and MutLγ proteins and how *K*. *l*. Zip1 might bypass the constraint. Cartoons depict potential pathways for SC protein-MutSγ-MutLγ associated crossovers in budding yeast. At left is depicted *S*. *cerevisiae* expressing *S*. *c*. *ZIP1*, while at right is depicted *S*. *cerevisiae* expressing *K*. *l*. *ZIP1*. In these models, Zip1 maintains a pro-crossover function along with other SIC proteins and Msh4/Msh5, upstream of stabilized dHJs. Both *S*. *c*. Zip1 and *K*. *l*. Zip1 collaborate with other SIC proteins and Msh4/Msh5 to promote the establishment of stable JMs. In this model, *S*. *c*. Zip1 also has an anti-crossover activity that Msh4/Msh5 normally counters (left cartoon). This anti-crossover activity might be an intrinsic feature of the Zip1 protein or perhaps is an activity coming from SC formation. An antagonistic relationship between Msh4/Msh5 and *S*. *c*. Zip1 constrains Zip1-mediated, MutLγ-associated resolvase activity to act exclusively on MutSγ-associated recombination intermediates. In the context of *S*. *cerevisiae* cells expressing *K*. *l*. *ZIP1* (right cartoon), *K*. *l*. Zip1 retains the pro-crossover activity but lacks the anti-crossover activity of *S*. *c*. Zip1, rendering MutSγ dispensable for MutLγ-dependent crossover recombination. Since *K*. *l*. Zip1 uncouples Mlh3-mediated crossover formation from both SC assembly and from a reliance on Msh4, we are drawn to the possibility that SC assembly normally constrains Zip1 crossovers–ensuring that they occur successfully only on Msh4-associated intermediates. We emphasize that the anti-crossover aspects of Zip1 activity could either be an action that destabilizes JM structures or one that prevents the accessibility of JM structures to MutLγ and other resolvase proteins.

On the other hand, Fig 9 presents an alternative model to explain both the mechanism that normally constrains MutLγ activity to target MutSγ-associated recombination intermediates in *S*. *cerevisiae* and how *K*. *l*. Zip1 bypasses this constraint. In our alternative model, we propose that *S*. *c*. Zip1 is normally associated with both pro-crossover and anti-crossover activities, and that Msh4/Msh5 counters the anti-crossover activity of Zip1 at JMs. A possible anti-crossover aspect of Zip1 activity could be an action that destabilizes dHJ structures, or one that prevents the accessibility of dHJ structures to MutLγ-associated resolvase activity. The presence of MutSγ at JMs might protect them or directly counter Zip1’s anti-crossover activity. In the context of *S*. *cerevisiae* cells expressing *K*. *l*. *ZIP1*, *K*. *l*. Zip1 retains *S*. *c*. Zip1’s pro-crossover activity but lacks its anti-crossover activity, thus rendering MutSγ dispensable for the MutLγ-mediated resolution of Zip1/SC protein-associated recombination intermediates.

The proposed antagonistic relationship between Msh4/Msh5 and *S*. *c*. Zip1 that this model proposes would effectively constrain MutLγ-mediated resolvase activity to act exclusively on MutSγ-associated recombination intermediates: Although *S*. *c*. Zip1 may be able to promote the formation of JM structures in the absence of MutSγ, only MutSγ-associated crossover intermediates are processed during meiosis in a manner that protects them from *S*. *c*. Zip1’s anti-crossover activity and allows their resolution by a MutLγ-mediated mechanism.

It is tantalizing to suggest that the putative “crossover constraining” activity of *S*. *c*. Zip1 proposed by this model is the process of SC assembly or the assembled SC itself. Perhaps the Msh4/Msh5 complex is required to protect the integrity of SEI and dHJ recombination intermediates during the process of SC elaboration, or to maintain the accessibility of such intermediates to resolvases in the context of full length SC later on. Under this scenario, the absence of SC in *K*. *l*. *ZIP1*-expressing cells renders MutSγ complexes dispensable for MutLγ-dependent crossover formation.

### A role for Zip1 or the SC in crossover patterning?

In budding yeast, Zip1, Msh4/Msh5, and Mlh1/Mlh3 are associated with the successful resolution of crossover recombination intermediates that exhibit interference [25,27,30,34,70,90]. However, SC proteins likely play no role in the initial establishment of an interfering distribution pattern of SC-associated (MutSγ-MutLγ-mediated) crossover recombination events. Such a conclusion is supported by the fact that early crossover-correlated recombination intermediates form at a stage of meiotic prophase that is prior to when full-length SCs are present [27]. Furthermore, Zip2 and Zip3 chromosomal foci, which co-localize at SIC structures and are presumably cytological manifestations of SC-associated crossovers, exhibit an interfering distribution on meiotic pachytene chromosomes even when Zip1 is absent [14,91]. However, while the initial establishment of interfering crossover events may not require SC-associated proteins, the fact that Mlh3-dependent crossovers exhibit diminished interference in *K*. *l*. *ZIP1*-expressing *S*. *cerevisiae* cells indicates that SC protein activity may well influence not only the resolution of, but the ultimate pattern of MutLγ-associated crossover events.

Indeed, taken to the extreme, a model postulating that SC proteins and/or SC structures play no role in crossover interference predicts that if MutSγ-MutLγ-dependent crossovers could successfully mature in the absence of SC, these crossovers would exhibit normal interference. Our data, however, show that *K*. *l*. Zip1 can promote wild-type levels of Mlh3-dependent crossovers in a subset of *S*. *cerevisiae* meiotic cells, but these crossovers exhibit a substantially weakened interference pattern (Table 2 and Fig 6). As the cytological manifestation of MutLγ-associated crossovers (Zip2 or Zip3 foci) exhibit an interfering distribution pattern even in strains missing Zip1 altogether [14,91], we assume that *K*. *l*. Zip1-mediated crossovers are designated with proper interference in *K*.*l*. *ZIP1*–expressing *S*. *cerevisiae* meiotic cells. If the designation of interfering crossovers is intact in strains expressing *K*. *l*. *ZIP1*, then the preservation of an interfering distribution pattern of crossover-designated recombination events apparently requires a Zip1 activity that is separable from its crossover promoting function *per se*.

The influence of Zip1 on crossover patterning could be explained if an interfering pattern of designated crossover events is different depending on the stage in meiotic prophase when those events are initiated. Perhaps the presence of *S*. *c*. Zip1 (and/or the SC) preserves the integrity of a discrete set of “earliest designated” crossover intermediates, which exhibit a robust interference pattern. When *K*. *l*. Zip1 is present, perhaps fewer of these earliest-designated intermediates undergo successful maturation into stable JM intermediates. Since the SC structure may possibly be a barrier to the formation of ongoing interhomolog recombination-based interactions [77], it is reasonable to speculate that SC protein-mediated interhomolog recombination events may initiate in an ongoing manner and occur at later meiotic prophase stages in a *K*. *l*. *ZIP1* (synapsis-defective) context, relative to normal *S*. *cerevisiae* meiosis. Thus, that subset of *K*. *l*. *ZIP1*-expressing meiotic cells carrying a wild-type crossover level may ultimately exhibit a crossover landscape that includes both early- and late-designated crossover intermediates. If the distribution of later prophase-designated recombination intermediates is less subject to interference, the result would be an overall weakening of the interfering distribution pattern of Mlh3-resolved crossovers in *K*. *l*. *ZIP1*-expressing cells.

## Methods

### Strains

All strains used in this study (S6 Table) are isogenic to BR1919-8B [69]. Strain variants were created by standard genetic crosses and transformation procedures. Every strain in which the *K*. *l*. *ZIP1* open reading frame replaced the *S*. *c*. *ZIP1* open reading frame was derived from the same parent, CO1. CO1 was created by first inserting *URA3* in place of *S*. *c*. *ZIP1* open reading frame sequences. Next, a PCR product containing the *K*. *l*. *ZIP1* open reading frame (amplified off of genomic DNA extracted from *K*. *lactis* cells) flanked by ~50 bp of homology to the 5’ and 3’ sequences of the *S*. *c*. *ZIP1* endogenous locus was transformed into the *zip1*::*URA3* strain, in order to replace the *URA3* sequences at the *ZIP1* locus with *K*. *l*. *ZIP1* sequences. Primers used for this step were: 5’TTCTTTGAGATTCGGAAGTAAAATACCCTCGGCGGCTAAATTTTTAGAGAATGTCTAACTTCTTCAGAGACAACTCG 3’ and 5’ACAAAATGAAATGTATTCGCACAAAACGATTTCAAATTTTCCATTATCCTTTATCTGAATCTTTTGGTCTTTTTTAATCGAGG 3’ (underlined regions correspond to *K*. *l*. *ZIP1* sequences). Counterselection against Ura+ was carried out using 5-FOA medium.

The *K*.*lactis ZIP1*-V5 fusion cassette was created by first inserting *URA3* between the codons for amino acids 472 and 473 of *K*. *lactis* Zip1. Next, a PCR product with flanking homology to *K*.*lactis ZIP1* but carrying an in-frame V5 sequence was used to counterselect against Ura+ cells on 5-FOA medium. DNA sequencing confirmed the position of V5 coding sequences in frame with the codon for amino acid 472 in an otherwise complete *K*.*lactis ZIP1* gene.

To construct a haploid strain capable of sporulation, *MATa* was integrated at the *THR1* locus in a haploid *MATα* strain, using the B211 plasmid from Beth Rockmill [69].

Strains used for crossover analysis in spores carry a *hphMX* cassette inserted near the chromosome III centromere, *ADE2* inserted upstream of the *RAD18* locus, a *natMX* cassette inserted near the *HMR* locus, *TRP1MX4* was inserted just downstream of the *SPO11* locus, and *LEU2* and *THR1* were inserted on chromosome XI at 152kb, and at 193,424bp, respectively.

Chromosome III circular *MATα* strains as well as TY521 and TY522 [18] were received from the Roeder lab.

### Cytological analysis and imaging

Meiotic nuclei were surface spread on glass slides and imaged as described in [13]. The following antibodies were used: rabbit anti-Zip1 (created as described in [10]), rabbit anti-Red1 [61], guinea pig anti-SUMO [11], chicken anti-HA (Abcam), mouse anti-MYC (clone 9E10, Invitrogen), rabbit anti-V5 (Abcam). Secondary antibodies conjugated with Alexa Fluor dyes were purchased from Life Technologies and used at a 1:200 dilution.

### Calculations and statistical analysis

Genetic crossover data was compiled and processed using an Excel Linkage Macro program, created by Jonathan Greene (Rhona Borts, pers. comm.) and donated by Eva Hoffmann (University of Sussex, UK). Final crossover and interference values (and their standard errors) were obtained using the Stahl lab online tools (http://molbio.uoregon.edu/~fstahl/), with the method of Perkins [92]. All other statistical analyses were carried out using Graphpad Prism or Graphpad InStat (www.graphpad.com).

### Pulsed field gel electrophoresis, Southern blotting, western blot

Agarose plugs were prepared from meiotic cultures at 0, 40 and 70 hours of sporulation and subjected to pulsed-field gel analysis [18,79]. For Southern blotting, a 1 kb probe from the *THR4* region of chromosome III was prepared using a DIG High Prime DNA Labeling and Detection Kit (Roche). A Syngene “G:Box” was used to detect chemiluminescence and the Syngene “Gene-Tools” program was used to analyze the data. A value for % recombination (Fig 7) was calculated by summing twice the intensity of the trimer band (a double crossover product) plus the dimer band (product of a single crossover) over the total intensity of the three bands (trimer, dimer and monomer). Note that circular chromosome III chromatids do not enter the gel, and thus are not included in the calculation to estimate recombination. The average of two experiments is presented.

Western blotting was performed as described previously [13].

### 2D gel electrophoresis and JM analysis

2D gel electrophoresis followed by Southern analysis to assay JMs was performed as previously described [35,66,93] Probes for detection of JMs at the *ERG1* locus [77,81] were amplified from yeast genomic DNA with primers- 5’-GGCAGCAACATATCTCAAGGCC-3’ and 5’-TCAATGTAGCCTGAGATTGTGGCG-3’. Probes for detection of JMs at *YCR047c* [35,80] were amplified from yeast genomic DNA using primers 5’-GGAATTCCGAGAGAATCGACTTGCTAA-3’ and 5’-GGAATTCCAGCCACCAGTGGGCTTTTC-3’. Hybridization signal was detected and quantified using a Typhoon FLA 9000 (GE) and the ImageJ software (http://imagej.nih.gov/ij/).

## Supporting Information

S1 Fig *K*. *l*. Zip1-V5 forms polycomplex and foci on *S. cerevisiae* meiotic chromosomes. (Related to Figs 2 and 3) Cartoon in (A) shows the V5-tag inserted after arginine 472. The V5-tagged *K*. *l*. Zip1 protein rescues the sporulation efficiency and spore viability defects of *S*. *c*. *zip1* null diploids to the same extent as untagged *K*. *l*. Zip1, as shown in (B). Images in (C) show examples of surface-spread meiotic pachytene nuclei from *S*. *cerevisiae* cells expressing *K*. *l*. *ZIP1-V5* and carrying one copy of *ECM11-MYC* (AM3356). Pachytene nuclei were harvested and surface-spread 24 hours after placement in sporulation medium. AM3356 cells are homozygous for an *ndt80* null allele, and thus will not progress beyond the pachytene stage of meiotic prophase. Immunolocalization with anti-V5 and anti-MYC antibodies was used to label *K*. *l*. Zip1-V5 (green) and Ecm11-MYC (red) on meiotic chromosomes (labeled with DAPI, white in first column and blue in second and third columns). In any given nucleus, a subset of the sparse *K*. *l*. Zip1 foci appeared overlapping with or adjacent to a fraction of Ecm11-MYC foci, and overlapping *K*. *l*. Zip1-V5 and Ecm11-MYC is frequently found at polycomplex structures, such as the one visible in the lower panels. Scale, 1 micron.
(TIF)

S2 Fig *K*. *l*. *ZIP1*-expressing and *zip1* null cells display fewer co-localized Zip3-MYC and Zip4-HA foci, relative to *S*. *c*. *ZIP1* cells. (Related to Fig 5.) The scatterplot in (A) shows the number of Zip3-MYC (red dots) and Zip4-HA (green dots) foci counted per nucleus in *S*. *c*. *ZIP1*-expressing (AM3362), *K*. *l*. *ZIP1*-expressing (AM3361), or *zip1* null (AM3363) strains. Each circle in the scatterplot in (B) represents a percent co-localization value for Zip3-MYC and Zip4-HA foci per nucleus. While the total numbers of Zip3-MYC and Zip4-HA foci measured in any particular nucleus were similar, they were not always precisely the same. To arrive at a % co-localization value in cases where the total number of Zip3-MYC and Zip4-HA were different from one another, the denominator used corresponded to the protein (Zip3-MYC or Zip4-HA) that displayed the fewest total foci in a given nucleus.
(TIF)

S3 Fig *K*. *l*. Zip1 sometimes localizes to the centromeres of *S*. *cerevisiae* meiotic chromosomes. *S*. *cerevisiae* meiotic cells expressing *K*. *l*. *ZIP1* (CO58) were surface-spread at 2 hour intervals during sporulation, beginning at 12 hours after entry into sporulation medium and ending at 24 hours. Immunolocalization was used to label *K*. *l*. Zip1 (green) and Ctf19-MYC (red) on meiotic chromosomes (labeled with DAPI, white in first column and blue in second and third columns). In any given nucleus, sparse *K*. *l*. Zip1 foci appeared overlapping with or adjacent to a fraction of Ctf19-MYC foci (white arrows in merged panels). Table below the images displays quantification of co-localization data. Scale, 1 micron.
(TIF)

S4 Fig SC independent centromere associations in *K*. *l*. *ZIP1*-expressing cells. The *y*-axis of the top row graphs indicates the number of nuclei in an observed diploid population that exhibited a given number of Ctf19-MYC foci (indicated on the *x* axis). Values are from five independent experiments, with 50 nuclei recorded for each of three strains: Diploid *S*. *cerevisiae* cells carrying a *spo11* null allele, and carrying either *S*. *c*. *ZIP1* (YT15), *zip1* null (YT21) or *K*. *l*. *ZIP1* (YT14) alleles. Images show surface-spread nuclei from the strains indicated in the top row graphs, labeled with Ctf19-MYC (white at top and red below) and DAPI (blue). Graphs in the middle row are analogous to the graphs above, except these data were calculated for haploid *spo11* null meiotic cells carrying either *S*. *c*. *ZIP1* (YT24), *zip1* null (YT25) or *K*. *l*. *ZIP1* (YT23) alleles. Values are from three independent experiments, with 50 nuclei recorded for each of the haploid strains. Bottom graphs indicate the frequency of nuclei exhibiting various numbers of Ctf19-MYC foci in *SPO11* haploid meiotic cells carrying either *S*. *c*. *ZIP1* (YAM538), *zip1* null (AM2841) or *K*. *l*. *ZIP1 (*AM2840) alleles. The red box on the *x*-axis of each graph indicates the expected number of Ctf19-MYC foci in each cellular context if centromere pairwise associations are robust.
(TIF)

S5 Fig Msh4-HA levels are diminished on mid-meiotic prophase chromosomes from *K*. *l*. *ZIP1*-expressing cells. (Related to Figs 6 and 7 and S2). Images in (A) show examples of surface-spread meiotic pachytene nuclei from *S*. *cerevisiae* cells expressing *MSH4-HA* as well as *ZIP3-MYC* (AM3411, AM3412, AM3413). Pachytene nuclei were harvested and surface-spread 24 hours after placement in sporulation medium. Cells from all strains are homozygous for an *ndt80* null allele, and thus will not progress beyond the pachytene stage of meiotic prophase. Immunolocalization with anti-HA and anti-MYC antibodies was used to label Msh4-HA and Zip3-MYC on meiotic chromosomes (labeled with DAPI, white in first column and blue in second and third columns). The scatterplot in (B) shows the number of Zip3-MYC (red dots) and Msh4-HA (green dots) foci counted per nucleus in *S*. *c*. *ZIP1*-expressing (AM3412), *K*. *l*. *ZIP1*-expressing (AM3411), or *zip1* null (AM3413) strains. Each circle represents a nucleus.
(TIF)

S6 Fig The formation of *K*. *l*. Zip1-promoted crossovers is dependent on *NDT80*. (Related to Fig 7.) Three independent sporulating cultures (A, B, C) of *S*. *cerevisiae ndt80Δ* strains carrying one linear and one circular chromosome III and carrying either *S*. *c*. *ZIP1* (K663), *K*. *l*. *ZIP1* (K666) or a *zip1* null (K669) allele were embedded in agarose plugs, processed, run on a pulsed-field gel, and analyzed by Southern blot using a probe to chromosome III sequences (see Methods). In addition, an analogous strain but expressing *NDT80* and *K*. *l*. *ZIP1* was processed as a control (far right). Aliquots of sporulating cells were taken at 0, 40, and 70 hours after placement in sporulation medium, but only the 70 hour time points are shown on this blot. The lowest band represents the size of endogenous (linear) III, while the middle and upper bands (seen in the *NDT80* strain) represent the product of crossing over between the linear and the circular III (see Fig 7). In contrast to *NDT80* strains (far right and Fig 7), no evidence of recombinant chromosome III is detected at the 70 hour time point for any the *ndt80Δ* strain replicates.
(TIF)

S7 Fig *S*. *c*. Zip1 and *K*. *l*. Zip1 promote Msh4-independent JM formation in *S*. *cerevisiae* meiotic cells. (Related to Fig 8.) Sporulating cultures of *S*. *cerevisiae* strains carrying either *S*. *c*. *ZIP1* (K663), *K*. *l*. *ZIP1* (K666) or a *zip1* null (K669) allele in the *MSH4* background (top half) or *S*. *c*. *ZIP1* (K672), *K*. *l*. *ZIP1* (K675) or a *zip1* null (K678) allele in the *msh4* background (bottom half) were subject to psoralen crosslinking to preserve recombination intermediates (JMs; see Methods). Aliquots of sporulating cells were taken at 0 and 32 hours after placement in sporulation medium and crosslinked DNA was separated by 2D gel electrophoresis. In this assay, the linear DNA (including non-JM parental DNA) travels as an arc while branched recombination intermediates (including JMs) are slower migrating and are retarded from the linear arc. These molecules can be detected by Southern hybridization as shown in the schematic in Fig 8. The percentage of JM/total DNA exhibited by each strain at the *YCR047c* locus in a representative time course experiment is given next to each box. Time course experiments were analyzed at least twice with similar trends observed in each experiment.
(TIF)

S1 Table Map distances measured in spores from *non*-4 spore-viable tetrads. Random spore analysis was used to calculate map distances and standard errors between four intervals on chromosome III, one interval on VIII and one interval on XI in *S*. *c*. *ZIP1*- expressing and *K*. *l*. *ZIP1*-expressing strains (YT131, YT125, AM3313 and YT152). cM = (# recombinant spores for the interval /total spores examined) x100. Standard Error (S. E.) values were calculated according to the formula: 100x [(r/t) (1-(r/t))/t], where r = the number of recombinant spores and t = the total number of spores examined.
(PDF)

S2 Table Distribution of crossover types among all spores. Spores from 1-, 2-, 3- or 4- spore viable tetrads from sporulated YT131, YT125, AM3313 and YT152 strains were classified based on the allele configurations that they displayed for each of the chromosome III loci used in the crossover recombination analysis (Table 2). A similar fraction of single, double, triple and quadruple crossovers were formed on chromosomes III in *K*. *l*. *ZIP1*-expressing and *S*. *c*. *ZIP1 cells*. The absence of Msh4 caused a substantial reduction in double and triple crossover-chromosomes III in *S*. *c*. *ZIP1*-expressing cells, but only caused a small reduction in double and triple crossover chromosomes III in *K*. *l*. *ZIP1*-expressing cells.
(PDF)

S3 Table Spore viability of *S*. *cerevisiae* or *K*. *lactis ZIP1* crossover strains. Presented in this table is the distribution of tetrad types and total % of viable spores that were examined from *S*. *c*. *ZIP1*- expressing and *K*. *l*. *ZIP1*-expressing strains (YT131, YT125, AM3313 and YT152) for the crossover recombination analysis presented in Table 2.
(PDF)

S4 Table Gene conversion events per locus; measured in 4-spore viable tetrads. Cases of non-mendelian segregation events (non-2:2 segregation of alleles) at the indicated genetic loci in 4-spore viable tetrads derived from *S*. *c*. *ZIP1*- expressing and *K*. *l*. *ZIP1*-expressing strains (YT131, YT125, AM3313 and YT152). These data were extracted from the datasets gathered for the crossover recombination analysis presented in Table 2.
(PDF)

S5 Table “Interference ratio”: Measure the influence of recombination in one interval on the map distance of an adjacent interval on chromosome III. Data in this table is related to the “interference ratio” [63, 64] calculated for several intervals on chromosome III in the strains indicated at left. Two map distances (cM+/- S. E.) are generated for a given “test” interval, indicated in row two. The first indicated map distance is calculated using a set of tetrads in which an adjacent “reference” interval (indicated in the top row of the table) is non-recombinant according to genetic data (all of the tetrads in this group are Parental Ditype (PD or P) for the reference interval) whereas the second indicated map distance is derived from tetrads in which the reference interval exhibits crossover recombination (are Non-Parental Ditype (NPD or N) or Tetratype (TT or T) for the reference interval). The ratio for each test interval is the map distance obtained for an interval from tetrads associated with recombinant reference interval divided by the map distance obtained for the test interval from tetrads in which no recombination event is detected in the adjacent reference interval. *P* values were calculated from chi-square analysis (Instat, Graphpad.com) of the distribution of tetrad types derived from recombinant versus non-recombinant reference interval groups. In addition, statistical analysis of the significance of differences between map lengths calculated using tetrad types (performed using Stahl Online Tools) were used to determine whether adjacent intervals exhibited interference. Interference ratios for which a *P* value associated with significance and a significant difference between map lengths were calculated are considered to reflect positive interference between the reference interval and the test interval, and are indicated in bold.
(PDF)

S6 Table Strains used in this study. (PDF)
