# Supplementary figures and images for: Separable Crossover-Promoting and Crossover-Constraining Aspects of Zip1 Activity during Budding Yeast Meiosis
Source: PLoS Genet. 2015 Jun 26;11(6):e1005335. doi: 10.1371/journal.pgen.1005335 (PMC4482702; doi:10.1371/journal.pgen.1005335)

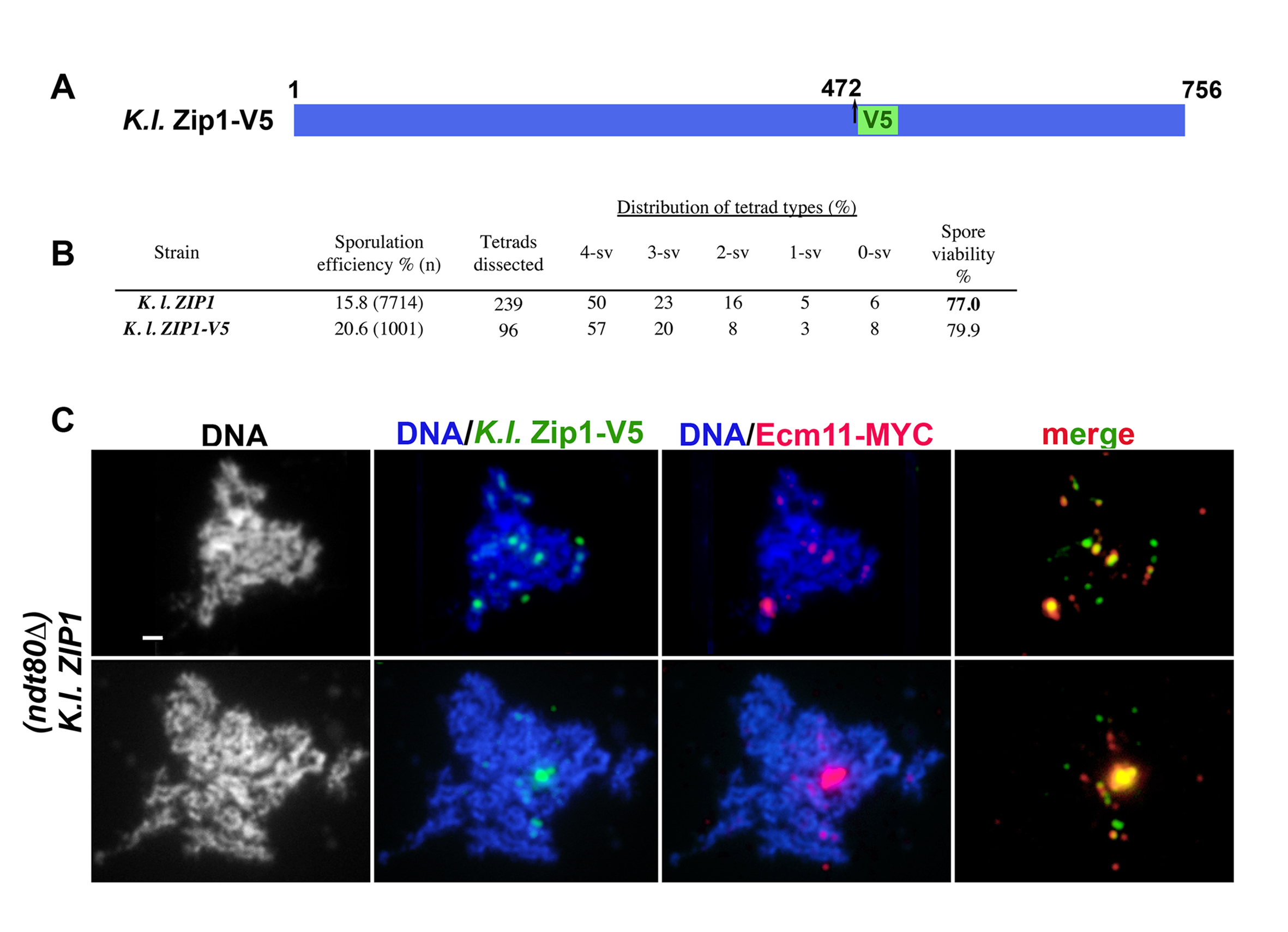

Supplement: S1 Fig — (Related to Figs 2 and 3) Cartoon in (A) shows the V5-tag inserted after arginine 472. The V5-tagged K. l. Zip1 protein rescues the sporulation efficiency and spore viability defects of S. c. zip1 null diploids to the same extent as untagged K. l. Zip1, as shown in (B). Images in (C) show examples of surface-spread meiotic pachytene nuclei from S. cerevisiae cells expressing K. l. ZIP1-V5 and carrying one copy of ECM11-MYC (AM3356). Pachytene nuclei were harvested and surface-spread 24 hours after placement in sporulation medium. AM3356 cells are homozygous for an ndt80 null allele, and thus will not progress beyond the pachytene stage of meiotic prophase. Immunolocalization with anti-V5 and anti-MYC antibodies was used to label K. l. Zip1-V5 (green) and Ecm11-MYC (red) on meiotic chromosomes (labeled with DAPI, white in first column and blue in second and third columns). In any given nucleus, a subset of the sparse K. l. Zip1 foci appeared overlapping with or adjacent to a fraction of Ecm11-MYC foci, and overlapping K. l. Zip1-V5 and Ecm11-MYC is frequently found at polycomplex structures, such as the one visible in the lower panels. Scale, 1 micron. (TIF) [file pgen.1005335.s001.tif]

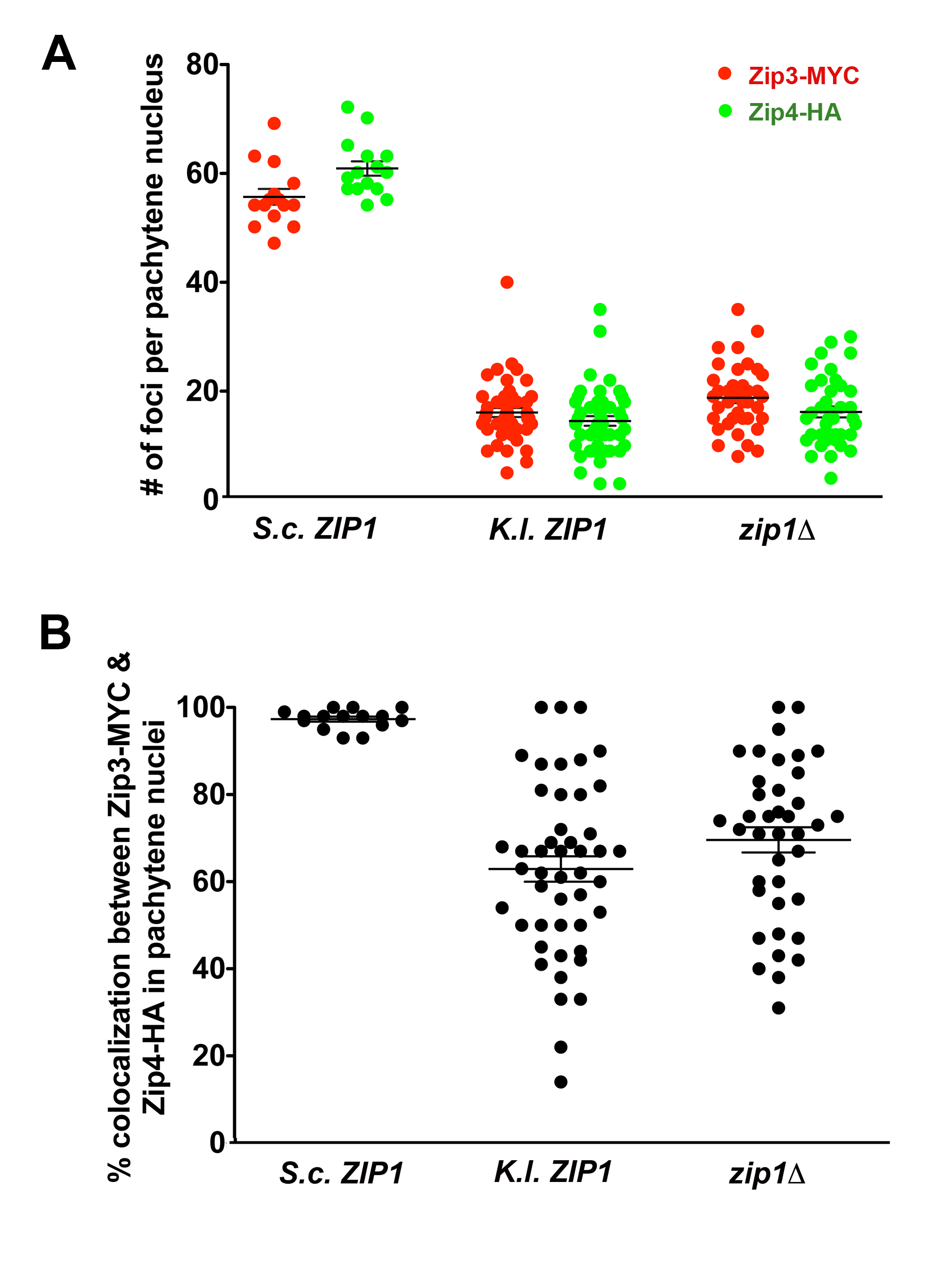

Supplement: S2 Fig — (Related to Fig 5.) The scatterplot in (A) shows the number of Zip3-MYC (red dots) and Zip4-HA (green dots) foci counted per nucleus in S. c. ZIP1-expressing (AM3362), K. l. ZIP1-expressing (AM3361), or zip1 null (AM3363) strains. Each circle in the scatterplot in (B) represents a percent co-localization value for Zip3-MYC and Zip4-HA foci per nucleus. While the total numbers of Zip3-MYC and Zip4-HA foci measured in any particular nucleus were similar, they were not always precisely the same. To arrive at a % co-localization value in cases where the total number of Zip3-MYC and Zip4-HA were different from one another, the denominator used corresponded to the protein (Zip3-MYC or Zip4-HA) that displayed the fewest total foci in a given nucleus. (TIF) [file pgen.1005335.s002.tif]

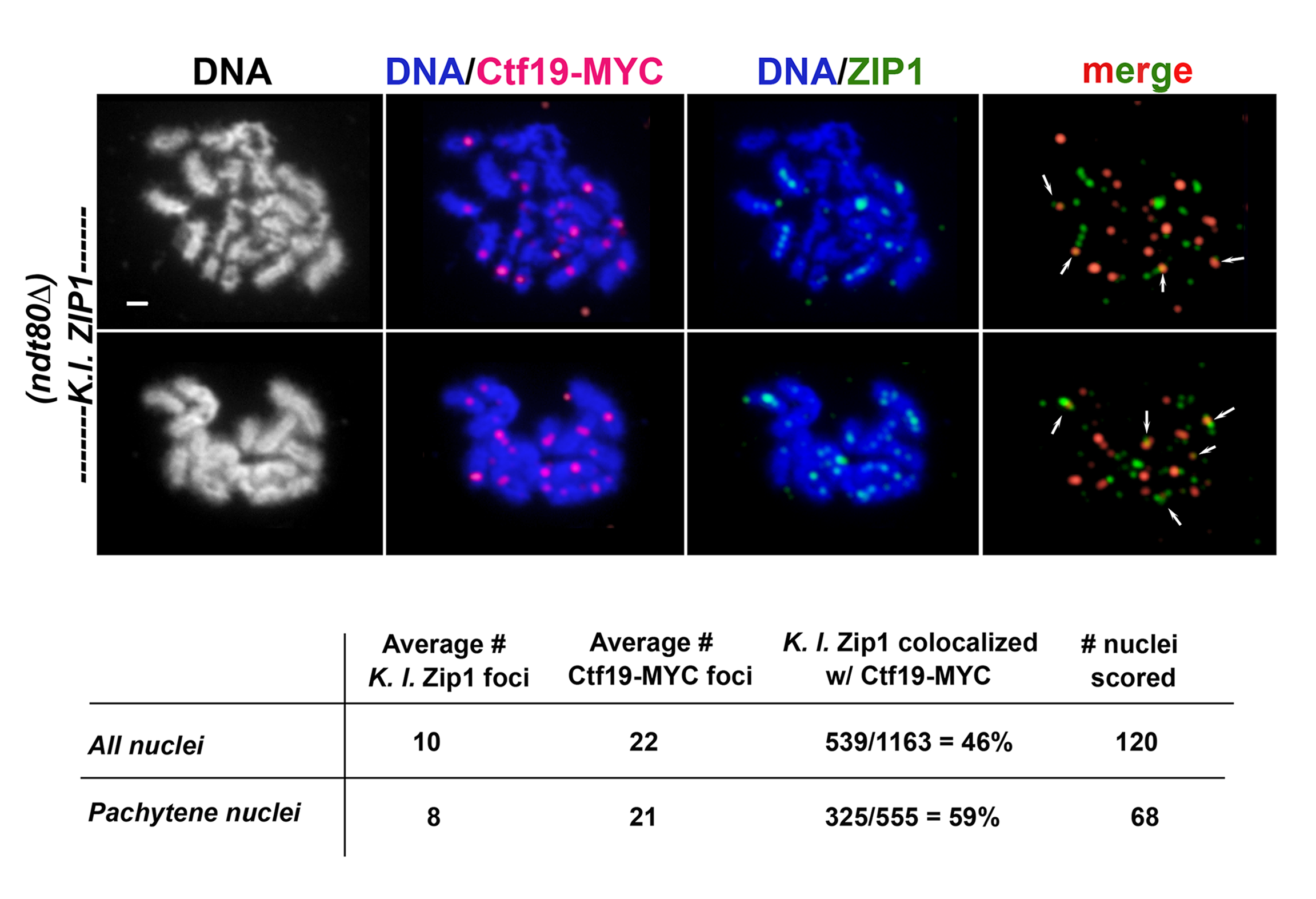

Supplement: S3 Fig — S. cerevisiae meiotic cells expressing K. l. ZIP1 (CO58) were surface-spread at 2 hour intervals during sporulation, beginning at 12 hours after entry into sporulation medium and ending at 24 hours. Immunolocalization was used to label K. l. Zip1 (green) and Ctf19-MYC (red) on meiotic chromosomes (labeled with DAPI, white in first column and blue in second and third columns). In any given nucleus, sparse K. l. Zip1 foci appeared overlapping with or adjacent to a fraction of Ctf19-MYC foci (white arrows in merged panels). Table below the images displays quantification of co-localization data. Scale, 1 micron. (TIF) [file pgen.1005335.s003.tif]

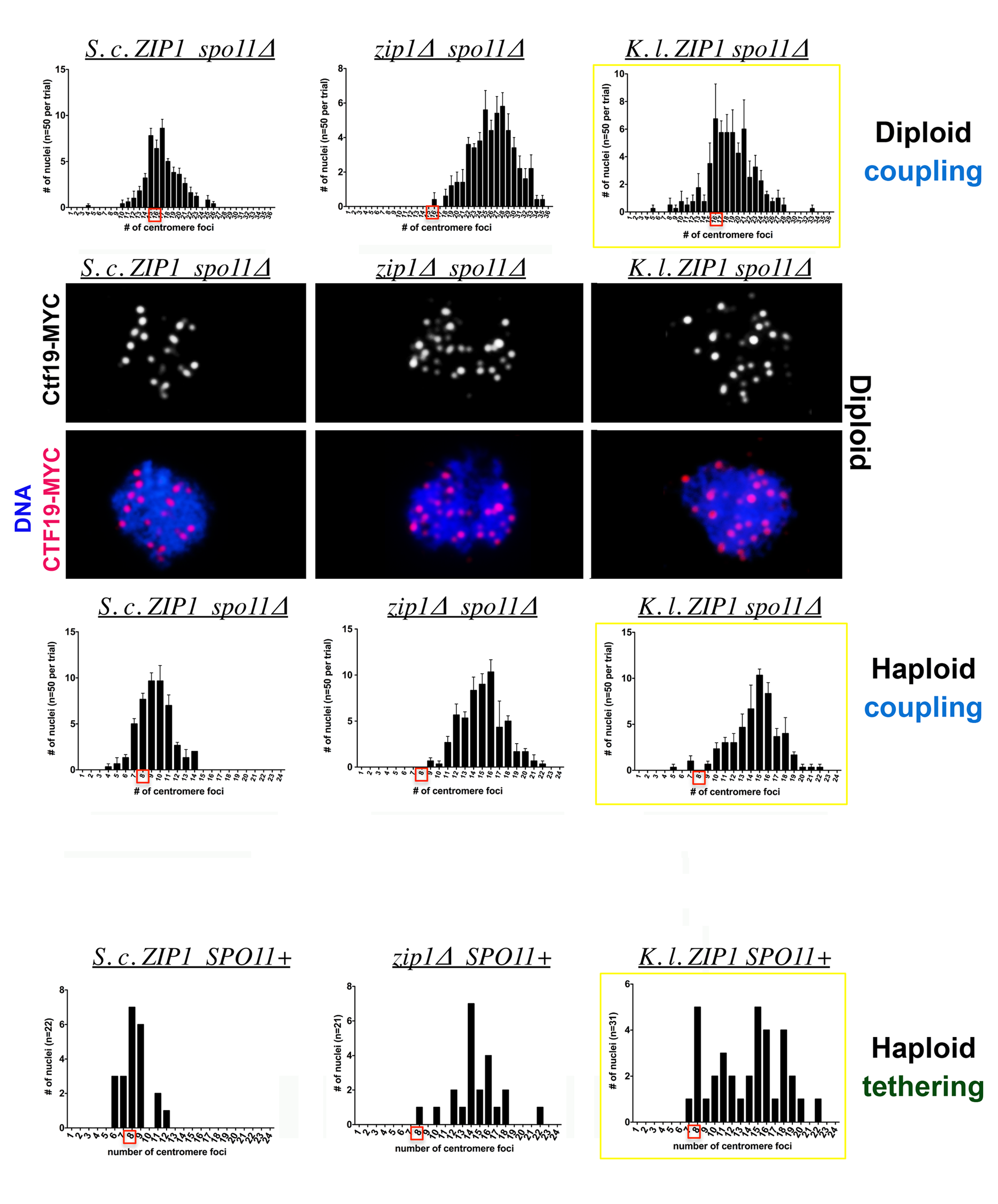

Supplement: S4 Fig — The y-axis of the top row graphs indicates the number of nuclei in an observed diploid population that exhibited a given number of Ctf19-MYC foci (indicated on the x axis). Values are from five independent experiments, with 50 nuclei recorded for each of three strains: Diploid S. cerevisiae cells carrying a spo11 null allele, and carrying either S. c. ZIP1 (YT15), zip1 null (YT21) or K. l. ZIP1 (YT14) alleles. Images show surface-spread nuclei from the strains indicated in the top row graphs, labeled with Ctf19-MYC (white at top and red below) and DAPI (blue). Graphs in the middle row are analogous to the graphs above, except these data were calculated for haploid spo11 null meiotic cells carrying either S. c. ZIP1 (YT24), zip1 null (YT25) or K. l. ZIP1 (YT23) alleles. Values are from three independent experiments, with 50 nuclei recorded for each of the haploid strains. Bottom graphs indicate the frequency of nuclei exhibiting various numbers of Ctf19-MYC foci in SPO11 haploid meiotic cells carrying either S. c. ZIP1 (YAM538), zip1 null (AM2841) or K. l. ZIP1 (AM2840) alleles. The red box on the x-axis of each graph indicates the expected number of Ctf19-MYC foci in each cellular context if centromere pairwise associations are robust. (TIF) [file pgen.1005335.s004.tif]

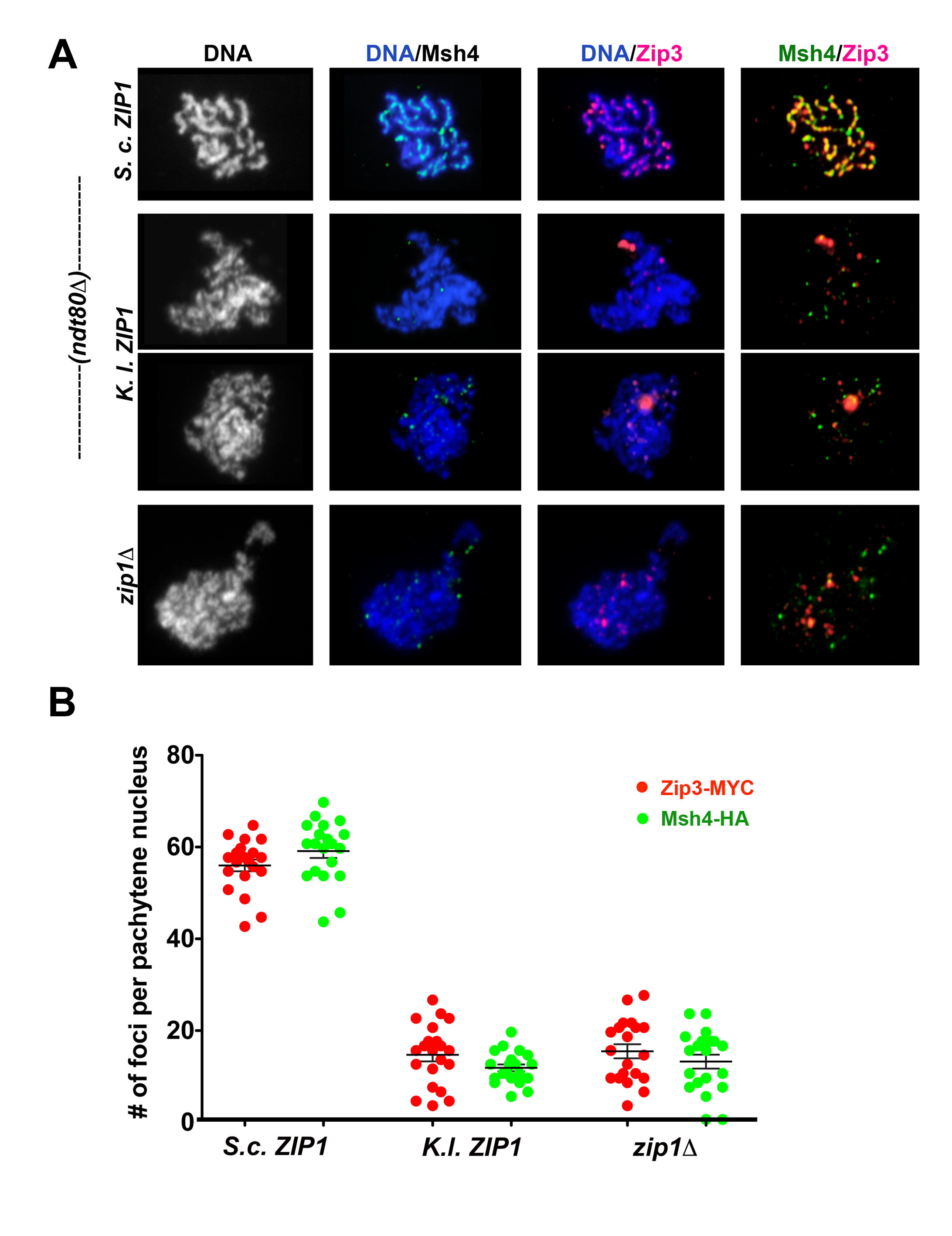

Supplement: S5 Fig — (Related to Figs 6 and 7 and S2). Images in (A) show examples of surface-spread meiotic pachytene nuclei from S. cerevisiae cells expressing MSH4-HA as well as ZIP3-MYC (AM3411, AM3412, AM3413). Pachytene nuclei were harvested and surface-spread 24 hours after placement in sporulation medium. Cells from all strains are homozygous for an ndt80 null allele, and thus will not progress beyond the pachytene stage of meiotic prophase. Immunolocalization with anti-HA and anti-MYC antibodies was used to label Msh4-HA and Zip3-MYC on meiotic chromosomes (labeled with DAPI, white in first column and blue in second and third columns). The scatterplot in (B) shows the number of Zip3-MYC (red dots) and Msh4-HA (green dots) foci counted per nucleus in S. c. ZIP1-expressing (AM3412), K. l. ZIP1-expressing (AM3411), or zip1 null (AM3413) strains. Each circle represents a nucleus. (TIF) [file pgen.1005335.s005.tif]

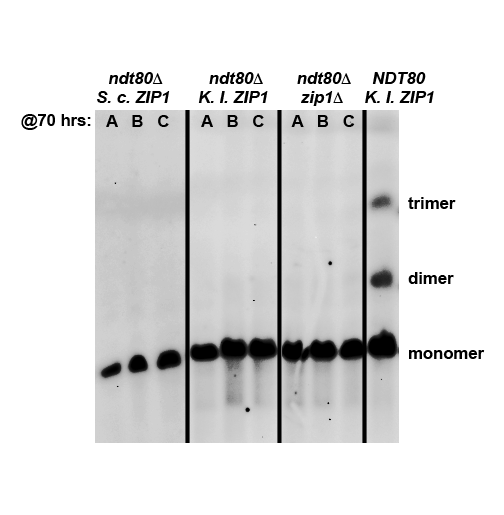

Supplement: S6 Fig — (Related to Fig 7.) Three independent sporulating cultures (A, B, C) of S. cerevisiae ndt80Δ strains carrying one linear and one circular chromosome III and carrying either S. c. ZIP1 (K663), K. l. ZIP1 (K666) or a zip1 null (K669) allele were embedded in agarose plugs, processed, run on a pulsed-field gel, and analyzed by Southern blot using a probe to chromosome III sequences (see Methods). In addition, an analogous strain but expressing NDT80 and K. l. ZIP1 was processed as a control (far right). Aliquots of sporulating cells were taken at 0, 40, and 70 hours after placement in sporulation medium, but only the 70 hour time points are shown on this blot. The lowest band represents the size of endogenous (linear) III, while the middle and upper bands (seen in the NDT80 strain) represent the product of crossing over between the linear and the circular III (see Fig 7). In contrast to NDT80 strains (far right and Fig 7), no evidence of recombinant chromosome III is detected at the 70 hour time point for any the ndt80Δ strain replicates. (TIF) [file pgen.1005335.s006.tif]

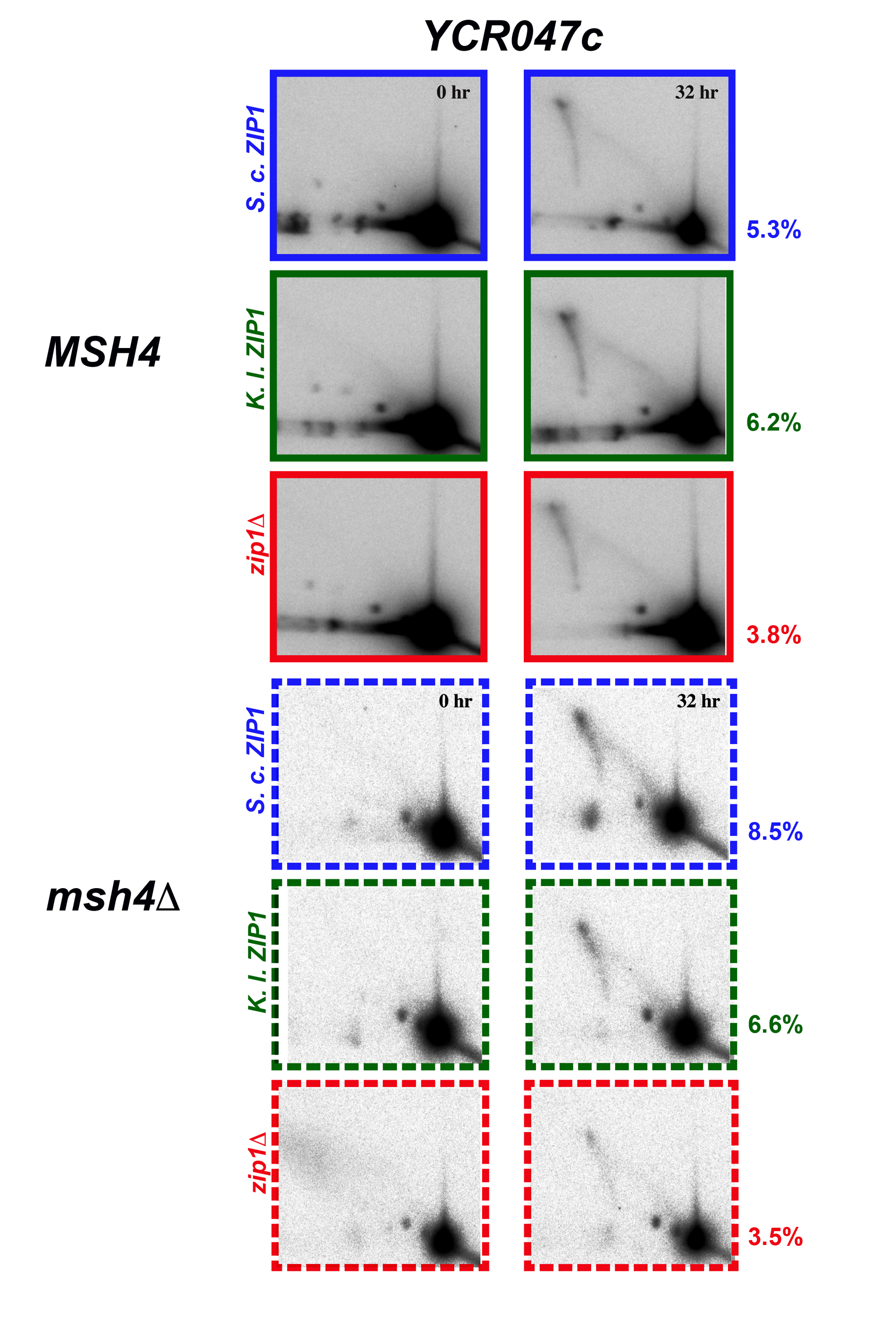

Supplement: S7 Fig — (Related to Fig 8.) Sporulating cultures of S. cerevisiae strains carrying either S. c. ZIP1 (K663), K. l. ZIP1 (K666) or a zip1 null (K669) allele in the MSH4 background (top half) or S. c. ZIP1 (K672), K. l. ZIP1 (K675) or a zip1 null (K678) allele in the msh4 background (bottom half) were subject to psoralen crosslinking to preserve recombination intermediates (JMs; see Methods). Aliquots of sporulating cells were taken at 0 and 32 hours after placement in sporulation medium and crosslinked DNA was separated by 2D gel electrophoresis. In this assay, the linear DNA (including non-JM parental DNA) travels as an arc while branched recombination intermediates (including JMs) are slower migrating and are retarded from the linear arc. These molecules can be detected by Southern hybridization as shown in the schematic in Fig 8. The percentage of JM/total DNA exhibited by each strain at the YCR047c locus in a representative time course experiment is given next to each box. Time course experiments were analyzed at least twice with similar trends observed in each experiment. (TIF) [file pgen.1005335.s007.tif]
