# Supplementary material for: Separable Crossover-Promoting and Crossover-Constraining Aspects of Zip1 Activity during Budding Yeast Meiosis
Source: PLoS Genet. 2015 Jun 26;11(6):e1005335. doi: 10.1371/journal.pgen.1005335 (PMC4482702; doi:10.1371/journal.pgen.1005335)
Supplement: S1 Table — Random spore analysis was used to calculate map distances and standard errors between four intervals on chromosome III, one interval on VIII and one interval on XI in S. c. ZIP1- expressing and K. l. ZIP1-expressing strains (YT131, YT125, AM3313 and YT152). cM = (# recombinant spores for the interval /total spores examined) x100. Standard Error (S. E.) values were calculated according to the formula: 100x [(r/t) (1-(r/t))/t], where r = the number of recombinant spores and t = the total number of spores examined. (PDF) [file pgen.1005335.s008.pdf]

**S1 Table. Map distances measured in spores from *non*-4 spore-viable tetrads**

| Strain | Interval (chromosome) | # Recombinant Spores | Total Spores | **cM** (S.E.) | **%WT** |
|---|---|---|---|---|---|
| *S.c. ZIP1 MSH4* | *HIS4-CEN3 (III)* | 67 | 274 | **24** (.03) | **100** |
| | *CEN3-MAT (III)* | 58 | 274 | **21** (.02) | **100** |
| | *MAT-RAD18 (III)* | 92 | 274 | **34** (.03) | **100** |
| | *RAD18-HMR (III)* | 48 | 274 | **18** (.02) | **100** |
| | *SPO11-SPO13 (VIII)* | 72 | 274 | **26** (.03) | **100** |
| | *iLEU2- iTHR1 (XI)* | 21 | 274 | **8** (.02) | **100** |
| *S.c. ZIP1 msh4Δ* | *HIS4-CEN3 (III)* | 70 | 1066 | **7** (.01) | **29** |
| | *CEN3-MAT (III)* | 75 | 1066 | **7** (.01) | **33** |
| | *MAT-RAD18 (III)* | 168 | 1066 | **16** (.01) | **47** |
| | *RAD18-HMR (III)* | 115 | 1066 | **11** (.01) | **61** |
| | *SPO11-SPO13 (VIII)* | 131 | 1066 | **12** (.01) | **46** |
| | *iLEU2- iTHR1 (XI)* | 59 | 1066 | **6** (.01) | **75** |
| *K.l. ZIP1 MSH4* | *HIS4-CEN3 (III)* | 348 | 1498 | **23** (.01) | **96** |
| | *CEN3-MAT (III)* | 371 | 1498 | **25** (.01) | **119** |
| | *MAT-RAD18 (III)* | 334 | 1498 | **22** (.01) | **65** |
| | *RAD18-HMR (III)* | 298 | 1498 | **20** (.01) | **111** |
| | *SPO11-SPO13 (VIII)* | 420 | 1498 | **28** (.01) | **108** |
| | *iLEU2- iTHR1 (XI)* | 135 | 1498 | **9** (.01) | **113** |
| *K.l. ZIP1 msh4Δ* | *HIS4-CEN3 (III)* | 344 | 1804 | **19** (.01) | **79** |
| | *CEN3-MAT (III)* | 336 | 1804 | **19** (.01) | **90** |
| | *MAT-RAD18 (III)* | 368 | 1804 | **20** (.01) | **59** |
| | *RAD18-HMR (III)* | 297 | 1804 | **16** (.01) | **89** |
| | *SPO11-SPO13 (VIII)* | 492 | 1804 | **27** (.01) | **104** |
| | *iLEU2- iTHR1 (XI)* | 167 | 1804 | **9** (.01) | **113** |
