# Supplementary material for: Separable Crossover-Promoting and Crossover-Constraining Aspects of Zip1 Activity during Budding Yeast Meiosis
Source: PLoS Genet. 2015 Jun 26;11(6):e1005335. doi: 10.1371/journal.pgen.1005335 (PMC4482702; doi:10.1371/journal.pgen.1005335)
Supplement: S2 Table — Spores from 1-, 2-, 3- or 4- spore viable tetrads from sporulated YT131, YT125, AM3313 and YT152 strains were classified based on the allele configurations that they displayed for each of the chromosome III loci used in the crossover recombination analysis (Table 2). A similar fraction of single, double, triple and quadruple crossovers were formed on chromosomes III in K. l. ZIP1-expressing and S. c. ZIP1 cells. The absence of Msh4 caused a substantial reduction in double and triple crossover-chromosomes III in S. c. ZIP1-expressing cells, but only caused a small reduction in double and triple crossover chromosomes III in K. l. ZIP1-expressing cells. (PDF) [file pgen.1005335.s009.pdf]

S2 Table. Distribution of crossover types among all spores

Chromosome III	*S. c. ZIP1*		*K.l. ZIP1*		*S. c. ZIP1 msh4Δ*		*K.l. ZIP1 msh4Δ*	
**genotype**	# spores	**% total**	# spores	**% total**	# spores	**% total**	# spores	**% total**
**Total number of spores**	**2341**		**3249**		**3636**		**4383**	
**Parental**	730	**31.2**	1162	**35.8**	2051	**56.4**	1883	**43.0**
**Single CO**								
HIS4-CEN3	230	9.8	334	10.3	191	5.3	344	7.8
CEN3-MAT	168	7.2	389	12.0	186	5.1	492	11.2
MAT-RAD18	450	19.2	351	10.8	604	16.6	471	10.7
RAD18-HMR	225	9.6	326	10.0	384	10.6	437	10.0
**total % single CO**		**45.8**		**43.1**		**37.5**		**39.8**
**Double CO**								
HIS4-CEN3; MAT-RAD	127	5.4	95	2.9	47	1.3	118	2.7
HIS4-CEN3; RAD18-HMR	83	3.5	68	2.1	22	0.6	108	2.5
HIS4-CEN3; CEN3-MAT	55	2.3	113	3.5	28	0.8	114	2.6
CEN3-MAT; MAT-RAD18	58	2.5	115	3.5	25	0.7	105	2.4
CEN3-MAT; RAD18-HMR	51	2.2	99	3.0	26	0.7	125	2.9
MAT-RAD18; RAD18-HMR	62	2.6	79	2.4	52	1.4	77	1.8
HIS4-CEN3; MAT-RAD18	12	0.5	4	0.1	1	0.0	0	0.0
**total % double CO**		**19.1**		**17.6**		**5.5**		**14.8**
**Triple CO**								
HIS4-CEN3-MAT-RAD18	34	1.5	23	0.7	3	0.1	25	0.6
CEN3-MAT-RAD18-HMR	12	0.5	23	0.7	4	0.1	22	0.5
HIS4-CEN3-MAT; RAD18-HMR	17	0.7	24	0.7	7	0.2	28	0.6
HIS4-CEN3; MAT-RAD18-HMR	24	1.0	36	1.1	3	0.1	28	0.6
**total % triple CO**		**3.7**		**3.3**		**0.5**		**2.3**
**Quadruple CO**								
HIS4-CEN3-MAT-RAD18-HMR	3	**0.1**	8	**0.2**	2	**0.1**	**6**	**0.1**
