# Supplementary material for: Separable Crossover-Promoting and Crossover-Constraining Aspects of Zip1 Activity during Budding Yeast Meiosis
Source: PLoS Genet. 2015 Jun 26;11(6):e1005335. doi: 10.1371/journal.pgen.1005335 (PMC4482702; doi:10.1371/journal.pgen.1005335)
Supplement: S3 Table — Presented in this table is the distribution of tetrad types and total % of viable spores that were examined from S. c. ZIP1- expressing and K. l. ZIP1-expressing strains (YT131, YT125, AM3313 and YT152) for the crossover recombination analysis presented in Table 2. (PDF) [file pgen.1005335.s010.pdf]

S3 Table. Spore viability of *S. cerevisiae* or *K. lactis ZIP1* crossover strains

Strain	Tetrads dissected	4 spore viable	3 spore viable	2 spore viable	1 spore viable	0 spore viable	**Spore viability %**
S.c. ZIP1 MSH4	616	528	54	29	5	0	**95**
S.c. ZIP1msh4Δ	1650	640	284	328	194	204	**65**
K.l. ZIP1 MSH4	2443	1065	537	485	201	155	**72**
K.l. ZIP1 msh4Δ	1596	629	314	331	175	147	**67**
