# Supplementary material for: Separable Crossover-Promoting and Crossover-Constraining Aspects of Zip1 Activity during Budding Yeast Meiosis
Source: PLoS Genet. 2015 Jun 26;11(6):e1005335. doi: 10.1371/journal.pgen.1005335 (PMC4482702; doi:10.1371/journal.pgen.1005335)
Supplement: S4 Table — Cases of non-mendelian segregation events (non-2:2 segregation of alleles) at the indicated genetic loci in 4-spore viable tetrads derived from S. c. ZIP1- expressing and K. l. ZIP1-expressing strains (YT131, YT125, AM3313 and YT152). These data were extracted from the datasets gathered for the crossover recombination analysis presented in Table 2. (PDF) [file pgen.1005335.s011.pdf]

S4 Table. Gene conversion events per locus; measured in 4-spore viable tetrads

		% non-mendelian segregation events								
Strain	# 4 spore viable tetrads analyzed	*HIS4*	*HYG@ CEN3*	*MAT*	*ADE2@ RAD18*	*natMX @ HMR*	*TRP1@ SPO11*	*spo13:: URA3*	*THR1@ chrm XI*	*LEU2@ chrm XI*
S. c. ZIP1 YT131	528	1.4	0	1	0.2	1.4	3.6	0	0.8	1.2
K. l. ZIP1 YT125	1065	4.7	1.1	3.8	3.1	0.8	4.1	1.6	2.5	2.4
S. c. ZIP1 msh4 AM3313	640	2.4	0.2	1.6	2.4	0.8	4.8	0.5	0.8	0.6
K. l. ZIP1 msh4 YT152	629	2.9	0.9	2.9	4.8	0.9	6.1	1.5	4.3	3.2
