# Supplementary material for: Separable Crossover-Promoting and Crossover-Constraining Aspects of Zip1 Activity during Budding Yeast Meiosis
Source: PLoS Genet. 2015 Jun 26;11(6):e1005335. doi: 10.1371/journal.pgen.1005335 (PMC4482702; doi:10.1371/journal.pgen.1005335)
Supplement: S5 Table — Data in this table is related to the “interference ratio” [63, 64] calculated for several intervals on chromosome III in the strains indicated at left. Two map distances (cM+/- S. E.) are generated for a given “test” interval, indicated in row two. The first indicated map distance is calculated using a set of tetrads in which an adjacent “reference” interval (indicated in the top row of the table) is non-recombinant according to genetic data (all of the tetrads in this group are Parental Ditype (PD or P) for the reference interval) whereas the second indicated map distance is derived from tetrads in which the reference interval exhibits crossover recombination (are Non-Parental Ditype (NPD or N) or Tetratype (TT or T) for the reference interval). The ratio for each test interval is the map distance obtained for an interval from tetrads associated with recombinant reference interval divided by the map distance obtained for the test interval from tetrads in which no recombination event is detected in the adjacent reference interval. P values were calculated from chi-square analysis (Instat, Graphpad.com) of the distribution of tetrad types derived from recombinant versus non-recombinant reference interval groups. In addition, statistical analysis of the significance of differences between map lengths calculated using tetrad types (performed using Stahl Online Tools) were used to determine whether adjacent intervals exhibited interference. Interference ratios for which a P value associated with significance and a significant difference between map lengths were calculated are considered to reflect positive interference between the reference interval and the test interval, and are indicated in bold. (PDF) [file pgen.1005335.s012.pdf]

S5 Table. "Interference ratio": Measure the influence of recombination in one interval on the map distance of an adjacent interval on chromosome III.

Genotype		Reference Interval	*HIS4- CEN3*		*CEN3-MAT*			*MAT-RAD18*			*RAD18-HMR*		
		**Test Interval**	*CEN3- MAT*	*MAT-RAD18*	*HIS4-CEN3*	*MAT-RAD18*	*RAD18-HMR*	*HIS4-CEN3*	*CEN3- MAT*	*RAD18-HMR*	*HIS4-CEN3*	*CEN3- MAT*	*MAT-RAD18*
***S. c.* ZIP1**													
YT131	*PD*	P:N:T	169:0:86	102:9:141	169:6:159	95:15:227	194:5:138	102:2:83	95:2:90	70:1:116	151:5:134	194:1:96	70:11:210
	**cM ± SE**		**16.9 ±1.5**	**38.7 ±3.5**	**29.2 ±2.4**	**47.0 ±3.2**	**24.9 ±2.3**	**25.4 ±2.8**	**27.3 ±2.8**	**32.6 ±2.3**	**28.3 ±2.6**	**17.5 ±1.7**	**47.4 ±3.2**
	TT+NPD	P:N:T	165:3:68	85:5:142	86:2;69	92:1:61	97:0:57	150:6:141	242:1:61	221:4:79	103:3:93	143:2:55	117:5:78
	**cM ± SE**		**18.2 ±2.6**	**37.1 ±3.0**	**25.8 ±3.2**	**21.8 ±2.7**	**18.5 ±2.0**	**29.8 ±2.7**	**11.0 ±1.5**	**16.9 ±2.3**	**27.9 ±3.0**	**16.8 ±2.6**	**27.0 ±3.5**
	**ratio**		1.08	0.96	0.88	**0.46**	0.74	1.17	**0.4**	**0.52**	0.99	0.96	**0.57**
X^2 P			0.11	0.39	0.66	**<0.0001**	0.2	0.54	**<0.0001**	**<0.0001**	0.98	0.30	**<0.0001**
Sig. SE (cM)			no	no	no	**yes**	yes	no	**yes**	**yes**	no	no	**yes**
S.c. ZIP1 msh4													
AM3313	*PD*	P:N:T	436:2:74	292:12:194	436:2:79	294:10:209	372:5:133	292:2:60	294:3:65	237:5:116	367:1:65	372:1:61	237:11:186
	**cM ± SE**		**8.4 ±1.1**	**26.7 ±2.2**	**8.8 ±1.1**	**26.2 ±2.0**	**16.0 ±1.6**	**10.2 ±1.5**	**11.5 ±1.7**	**20.4 ±2.2**	**8.2 ±1.1**	**7.7 ±1.1**	**29.0 ±2.4**
	TT+NPD	P:N:T	81:1:13	62:0:30	76:1:11	68:2:21	62:2:26	206:1:29	219:0:23	197:2:43	137:2:23	138:1:27	121:1:44
	**cM ± SE**		**10 ±3.5**	**16.3 ±2.4**	**9.7 ±3.8**	**18.1 ±5.0**	**21.1 ±5.0**	**7.4 ±1.6**	**4.8 ±0.9**	**11.4 ±2.1**	**10.8 ±2.9**	**9.9 ±2.3**	**15.1 ±2.4**
	**ratio**		1.23	0.61	1.1	0.69	1.32	0.73	**0.42**	**0.56**	1.32	1.29	**0.52**
X^2 P			0.69	0.13	0.53	0.01	0.49	0.29	**0.005**	**0.0002**	0.30	0.61	**0.0002**
Sig. SE (cM)			no	yes	no	no	no	no	**yes**	**yes**	no	no	**yes**
***K.l.* ZIP1**													
YT125	*PD*	P:N:T	164:3:119	151:3:111	164:7:102	159:4:102	154:4:104	151:1:106	159:7:102	164:3:103	163:7:118	154:7:118	164:4:115
	**cM ± SE**		**24.0 ±2.2**	**24.3 ±2.4**	**26.4 ±3.1**	**23.8 ±2.6**	**24.4 ±2.6**	**21.7 ±1.9**	**26.9 ±3.1**	**22.4 ±2.3**	**27.8 ±2.9**	**28.7 ±3.0**	**24.6 ±2.4**
	TT+NPD	P:N:T	109:5:78	107:3:72	122:1:82	109:2:87	125:3:69	114:5:70	106:2:87	119:4:72	110:1:72	108:2:70	106:2:74
	**cM ± SE**		**28.1 ±3.7**	**24.7 ±3.2**	**21.6 ±2.2**	**25.0 ±2.6**	**22.1 ±3.0**	**26.5 ±3.7**	**25.4 ±2.7**	**24.6 ±3.3**	**21.3 ±2.4**	**22.8 ±2.8**	**23.6 ±2.8**
	**ratio**		1.17	1.02	0.82	1.05	0.91	1.22	0.94	1.1	0.78	0.79	0.96
X^2 P			0.43	0.81	0.20	0.47	0.59	0.10	0.21	0.70	0.27	0.40	0.96
Sig. SE (cM)			no	no	no	no	no	no	no	no	no	no	no
K.l. ZIP1 msh4													
	PD	P:N:T	351:10:251	370:15:200	351:8:209	331:13:205	351:11:207	370:8:195	351:15:239	367:9:232	375:10:218	351:14:247	367:18:231
	**cM ± SE**		**25.4 ± 1.7**	**24.8 ± 2.1**	**22.6 ± 1.7**	**25.8 ± 2.1**	**24.0 ± 1.9**	**21.2 ± 1.7**	**27.2 ± 2.0**	**23.5 ± 1.7**	**23.1 ± 1.8**	**27.0 ± 2.0**	**27.5 ± 2.1**
	TT+NPD	P:N:T	217:11:122	202:5:122	261:12:121	254:8:131	261:2:128	215:7:121	218:7:132	249:5:105	225:5:112	218:8:122	241:3:107
	**cM ± SE**		**26.9 ± 2.9**	**23.1 ± 2.3**	**24.5 ± 2.7**	**22.8 ± 2.3**	**17.9 ± 1.6**	**23.8 ± 2.5**	**24.4 ± 2.4**	**18.8 ± 2.1**	**20.1 ± 2.2**	**24.4 ± 2.6**	**17.8 ± 1.9**
	**ratio**		1.06	0.93	1.08	0.88	0.75	1.12	0.9	0.8	0.87	0.9	**0.65**
X^2 P			0.07	0.43	0.04	0.40	0.07	0.68	0.61	0.02	0.54	0.26	**0.005**
Sig. SE (cM)			no	no	no	no	yes	no	no	no	no	no	**yes**
