# Supplementary material for: Separable Crossover-Promoting and Crossover-Constraining Aspects of Zip1 Activity during Budding Yeast Meiosis
Source: PLoS Genet. 2015 Jun 26;11(6):e1005335. doi: 10.1371/journal.pgen.1005335 (PMC4482702; doi:10.1371/journal.pgen.1005335)
Supplement: S6 Table — (PDF) [file pgen.1005335.s013.pdf]

S6 Table. Strains used in this study

	GENOTYPE
**YAM1252**	*lys2ΔNhe his4-260,519 leu2-3,112 MAT**α** trp1-289 S.c. ZIP1 ura3-1 thr1-4 ade2-1*<br>*lys2ΔNhe his4-260,519 leu2-3,112 MAT**a** trp1-289 S.c. ZIP1 ura3-1 thr1-4 ade2-1*
**CO9**	*lys2ΔNhe his4-260,519 leu2-3,112 MAT**α** trp1-289 K.l. ZIP1 ura3-1 thr1-4 ade2-1*<br>*lys2ΔNhe his4-260,519 leu2-3,112 MAT**a** trp1-289 K.l.ZIP1 ura3-1 thr1-4 ade2-1*<br><br>YAM1252 with *K. lactis ZIP1* ORF in place of the *S. cerevisiae ZIP1* ORF on chromosome IV.
**CO10**	YAM1252 homozygous *zip1::URA3*
**CO7**	YAM1252 *S. cerevisiae ZIP1/ zip1::URA3*
**CO11**	YAM1252 *K.lactis ZIP1/ zip1::URA3*
**CO33**	YAM1252 *S. cerevisiae ZIP1/ K.lactis ZIP1*
**AM2715**	YAM1252 homozygous *pch2:: TRP1*
**AM3340**	CO10 homozygous *pch2:: hphMX4*
**AM2713**	CO11 homozygous *pch2:: TRP1*
**K292**	YAM1252 *ndt80::LEU2 ECM11-cMYC::kanMX4 HIS4 hphMX4@CEN3 ADE2@RAD18*<br>*ndt80::LEU2 ECM11 his4-260,519 CEN3 RAD18*
**K268**	CO9 homozygous *ndt80::LEU2 and ECM11/ECM11-cMYC::kanMX4*
**AM3356**	CO9 *K.lactis ZIP1-V5* (tagged between R472 S473)
**AM2712**	YAM1252 *ECM11-cMYC::kanMX4 ndt80::LEU2*<br>*ECM11-cMYC::kanMX4 ndt80::LEU2*
**AM2711**	CO9 *ECM11-cMYC::kanMX4 ndt80::LEU2*<br>*ECM11-cMYC::kanMX4 ndt80::LEU2*
**AM2784**	CO10 *ECM11-cMYC::kanMX4 ndt80::LEU2*<br>*ECM11-cMYC::kanMX4 ndt80::LEU2*
**K375**	YAM1252 *ndt80::LEU2 ZIP3-cMYC HIS4 hphMX4@CEN3*<br>*ndt80::LEU2 ZIP3 his4-260,519 CEN3*
**YT12**	CO9 homozygous *ndt80::LEU2 and ZIP3/ZIP3-cMYC*
**AM3362**	YAM1252 *ZIP4-HA/ZIP4 ZIP3/ZIP3-cMYC*
**AM3361**	CO9 *ZIP4-HA/ZIP4 ZIP3/ZIP3-cMYC*
**AM3363**	CO10 *ZIP4-HA/ZIP4 ZIP3/ZIP3-cMYC ndt80::LEU2/NDT80*
**CO58**	CO9 homozygous *CTF19-cMYC::kanMX4*
**YT14**	CO9 homozygous *spo11::ADE2 CTF19-cMYC::kanMX4*
**YT15**	YAM1252 homozygous *spo11::ADE2 CTF19-cMYC::kanMX4*
**YT21**	CO10 homozygous *spo11::ADE2 CTF19-cMYC::kanMX4*
**YT24**	*lys2ΔNhe his4-260,519 leu2-3,112 MAT**α** trp1-289 ura3-1 spo11::ADE2*<br>***MATa**@THR1 ade2-1 CTF19-cMYC::kanMX4*
**YT23**	YT24 *K.lactis ZIP1*

**YT25**	YT24 *zip1::URA3*
**YAM538**	YT24 *SPO11*
**AM2840**	YT23 *SPO11*
**AM2841**	YAM538 *zip1::LYS2*
**K530**	YAM1252 *HIS4 leu2-Cla hphMX4@CEN3 MAT**α***<br>*his4-260,519 leu2-3,112 CEN3 MAT**a***<br><br>*CEN8 spo13::URA3 arg4-BglII thr1-4*<br>*TRP1@CEN8 SPO13 arg4-Nsp THR1*
**K531**	K530 homozygous *K.lactis ZIP1*
**K533**	K530 homozygous *zip1::URA3*
**YT131**	*HIS4 leu2-3,112 hphMX4@CEN3 MAT**a** ADE2@RAD18 natMX4@HMR trp1-289*<br>*his4-260,519 leu2-3,112 CEN3 MATα RAD18 HMR trp1-289*<br><br>*ura3-1 SPO11 spo13::URA3 thr1-4 LEU2@ChrmXI 152kb 192kb ade2-1*<br>*ura3-1 TRP1MX4@SPO11 SPO13 thr1-4 152kb THR1@193kb ade2-1*
**YT125**	YT131 homozygous *K.lactis ZIP1*
**AM3313**	YT131 homozygous *msh4::ADE2*
**YT152**	YT131 homozygous *K.lactis ZIP1 msh4::ADE2*
**K479**	YAM1252 *HIS4 leu2-CUP1 TRP1@CEN3 MAT**α** cup1*<br>*his4-260,519 leu2-3,112 CEN3 MAT**a** CUP1*<br>***MATα-**bearing chromosome III is circular*
**K457**	K479 homozygous *K.lactis ZIP1*
**TY521**	K479 homozygous *zip1::LEU2*
**K536**	K479 homozygous *mms4::natMX4*
**K521**	K457 homozygous *mms4::natMX4*
**K537**	K479 *zip1::LEU2/zip1::URA3* homozygous *mms4::natMX4*
**K542**	K479 homozygous *yen1::hphMX4*
**K545**	K457 homozygous *yen1::hphMX4*
**K548**	K479 *zip1::LEU2/zip1::URA3* homozygous *yen1::hphMX4*
**K573**	K479 homozygous *zip3::hphMX4*
**K576**	K457 *zip3::hphMX4/zip3::kanMX4*
**K579**	K479 *zip1::LEU2/zip1::URA3* homozygous *zip3::hphMX4*
**TY261**	K479 homozygous *zip4::kanMX4*
**K458**	K457 homozygous *zip4::kanMX4*
**TY522**	TY521 homozygous *zip4::kanMX4*

**K582**	K479 homozygous *spo16::hphMX4*
**K585**	K457 homozygous *spo16::hphMX4*
**K588**	K479 *zip1::LEU2/zip1::URA3* homozygous *spo16::hphMX4*
**K459**	K479 homozygous *msh4::ADE2*
**K486**	K457 homozygous *msh4::ADE2*
**K538**	K479 homozygous *zip1::URA3 msh4::ADE2*
**K551**	K479 homozygous *mlh3::hphMX4*
**K554**	K457 homozygous *mlh3::hphMX4*
**K557**	K479 *zip1::LEU2/zip1::URA3* homozygous *mlh3::hphMX4*
**K487**	K479 homozygous *zip4::kanMX4 msh4::ADE2*
**K488**	K457 homozygous *zip4::kanMX4 msh4::ADE2*
**K491**	K479 homozygous *zip1::LEU2 zip4::kanMX4 msh4::ADE2*
**K523**	K479 homozygous *mms4::natMX4 msh4::ADE2*
**K524**	K457 homozygous *mms4::natMX4 msh4::ADE2*
**K525**	K479 homozygous *zip1::URA3 mms4::natMX4 msh4::ADE2*
**K618**	K479 homozygous *zip4::kanMX4 mlh3::hphMX4*
**K621**	K457 homozygous *zip4::kanMX4 mlh3::hphMX4*
**K624**	K479 homozygous *zip1::LEU2 zip4::kanMX4 mlh3::hphMX4*
**AM3411**	CO9 homozygous *msh4::ADE2 MSH4-HA@URA3 ndt80::LEU2 ZIP3-MYC/ZIP3*
**AM3412**	YAM1252 homozygous *msh4::ADE2 MSH4-HA@URA3 ndt80::LEU2 ZIP3-MYC/ZIP3*
**AM3413**	CO10 homozygous *msh4::ADE2 MSH4-HA@URA3 ndt80::LEU2 ZIP3-MYC/ZIP3*
**K663**	K479 homozygous *ndt80::hphMX4*
**K666**	K457 homozygous *ndt80::hphMX4*
**K669**	K479 *zip1::LEU2/zip1::URA3* homozygous *ndt80::hphMX4*
**K672**	K459 homozygous *ndt80::hphMX4*
**K675**	K486 homozygous *ndt80::hphMX4*
**K678**	K538 homozygous *ndt80::hphMX4*
